# Bladder cancer in Africa, 2010–2023: burden, geographic heterogeneity, demographic drivers, pandemic-era temporal changes, and projections to 2030

**DOI:** 10.1186/s13027-026-00782-3

**Published:** 2026-07-23

**Authors:** Yongli Jin, Yan Jin, Yan Cui

**Affiliations:** 1 Department of Anesthesiology, The Affiliated Hospital of Yanbian University (Yanbian Hospital), Yanji, China; 2 Department of Hemodialysis Room, The Affiliated Hospital of Yanbian University (Yanbian Hospital), Yanji, China; 3 Department of Oncology, The Affiliated Hospital of Yanbian University (Yanbian Hospital), Yanji, China

**Keywords:** Bladder cancer, Global burden of disease, Trends, Das Gupta decomposition, COVID-19

## Abstract

**Purpose:**

To characterize the burden, temporal trends, geographic heterogeneity, demographic components, pandemic-era deviations, and projected age-standardized rates of bladder cancer across Africa.

**Methods:**

We conducted an ecological secondary analysis of modeled Global Burden of Disease Study 2023 estimates for the GBD aggregate labeled “African Union” and 54 African country locations from 2010 to 2023. Incidence, prevalence, deaths, and disability-adjusted life-years (DALYs) were assessed as counts and age-standardized rates (ASRs) per 100,000 population for both sexes combined. Trends were summarized using estimated annual percentage changes. Incidence trajectories were descriptively grouped using dynamic time warping, and changes in counts were partitioned into population-size, age-structure, and age-specific rate components using Das Gupta decomposition. Exploratory autoregressive integrated moving average models compared GBD-estimated rates in 2020–2023 with trajectories extrapolated from 2010 to 2019. Country-specific ASRs were projected through 2030 using log-linear models.

**Results:**

For the GBD aggregate labeled “African Union,” incident cases increased from 16,357 in 2010 to 31,503 in 2023, prevalent cases from 73,806 to 147,599, deaths from 9,261 to 16,467, and DALYs from 232,414 to 415,746. Corresponding ASRs increased from 3.5 to 4.4 for incidence, 13.9 to 18.3 for prevalence, 2.2 to 2.6 for deaths, and 48.1 to 56.1 for DALYs. The highest estimated incidence ASRs in 2023 occurred in Tunisia, Malawi, Libya, Egypt, and Mali, while several countries with low estimated baseline rates had rapid relative increases. Population-size change was the largest decomposition component, accounting for 39.8%–58.8% of the increase across outcomes, with additional contributions from age structure and age-specific rates. GBD-estimated incidence, mortality, and DALY rates during 2020–2023 were above the extrapolated point forecasts; however, this exploratory analysis did not establish a causal pandemic effect. The primary projection models yielded higher fitted 2030 ASRs in a majority of countries, although results varied across training windows.

**Conclusion:**

GBD 2023 estimates indicate an increasing but geographically heterogeneous bladder cancer burden across Africa. Population growth was the largest contributor to increasing counts, while age-structure and age-specific rate changes also contributed. Regionally tailored prevention, diagnostic and treatment capacity, and improved cancer surveillance are needed. Pandemic-era deviations and 2030 projections should be interpreted as model-dependent rather than causal or definitive forecasts.

**Supplementary Information:**

The online version contains supplementary material available at https://doi.org/10.1186/s13027-026-00782-3.

## Introduction

Bladder cancer remains a substantial global health problem. GLOBOCAN 2024 estimated 635,264 new cases and 227,626 deaths worldwide, ranking bladder cancer eighth in cancer incidence and thirteenth in cancer mortality [1]. The burden is strongly patterned by sex and age, occurring predominantly in men and older adults, and varies markedly across regions [1; 2]. Recent analyses of population-based cancer-registry and vital-statistics data indicate that incidence has stabilized or declined in many high-income populations but remains high or is increasing in some settings; elevated rates have also been reported in parts of Northern Africa [2; 3]. These geographic and temporal differences probably reflect the combined influence of population aging, tobacco exposure, occupational carcinogens, infection-related disease, diagnostic activity, and variation in cancer-registration practices [3; 4].

Risk-factor distributions are heterogeneous across Africa. Tobacco smoking is the leading established modifiable risk factor for bladder cancer, while occupational exposure to aromatic amines and polycyclic aromatic hydrocarbons represents another important external risk [4]. A GBD-based analysis estimated that, in 2019, the proportions of people aged 15 years or older who smoked were 18.1% in North Africa, 17.8% in Southern Africa, 10.5% in Central Africa, 8.8% in Eastern Africa, and 7.0% in Western Africa. Although these model-based proportions declined across all five subregions between 1990 and 2019, the estimated number of smokers in Africa increased from approximately 50 million to 90 million because of population growth [5]. Occupationally relevant exposures may occur in dye, rubber, painting, printing, and other industries involving aromatic amines or combustion-derived compounds [4]. Evidence specific to African settings remains limited; for example, a multicentre case–control study in Egypt found an association between agricultural work and bladder cancer among men, but agricultural work represented a composite exposure category rather than a specific identified carcinogen [6]. Consequently, occupational patterns across other African subregions remain insufficiently characterized.

Urogenital schistosomiasis constitutes a distinct, geographically focal pathway to bladder carcinogenesis. A GBD 2021 analysis estimated that the age-standardized prevalence of schistosomiasis from all species in Africa was 9,461.8 per 100,000 population in 2021 and identified a particularly high burden in West Africa [7]. However, this all-species estimate should not be equated directly with exposure to *Schistosoma haematobium* or with bladder cancer risk. Chronic *S. haematobium* infection is considered carcinogenic and is associated particularly with squamous cell carcinoma of the bladder [8]. Historical case–control evidence from Alexandria, Egypt, associated previous urinary schistosomiasis with increased bladder cancer risk after adjustment for smoking and occupational factors [8]. In a more recent hospital-based pathology series of 481 patients from Tanzania’s Lake Zone, squamous cell carcinoma accounted for 57.0% of bladder cancers, and *S. haematobium* eggs were detected in 25.2% of specimens and were associated with squamous histology [9]. These studies illustrate the continuing relevance of schistosomiasis in selected endemic freshwater communities but should not be extrapolated to contemporary national incidence or to all countries within a subregion.

Comparable epidemiological evidence across Africa nevertheless remains incomplete. An Africa-wide systematic review identified only 22 eligible incidence studies from 15 countries and reported very high between-study heterogeneity, underscoring the limited representativeness of available empirical data [10]. Cancer-surveillance capacity also varies substantially across sub-Saharan Africa and the Middle East and North Africa, with many settings lacking recent, nationally representative population-based cancer registration or complete vital-registration data [11; 12]. Low reported or modeled rates may therefore reflect genuinely lower disease occurrence, incomplete diagnosis or registration, greater reliance on modeled relationships, or a combination of these factors. The COVID-19 pandemic introduced an additional interpretive challenge. A systematic review documented interruptions and delays in cancer screening, diagnosis, and treatment in sub-Saharan Africa [13]; however, GBD 2023 cancer estimates did not comprehensively incorporate pandemic-related effects because sufficiently robust data were unavailable across locations and outcomes [14]. Analyses of 2020–2023 patterns should therefore be regarded as exploratory assessments of pandemic-era temporal deviations rather than causal estimates of COVID-19 effects.

To address these gaps, we analyzed modeled estimates from GBD 2023 for Africa overall and 54 African country locations from 2010 to 2023, with projections to 2030. We assessed counts and age-standardized rates of incidence, prevalence, deaths, and disability-adjusted life-years; characterized geographic and temporal heterogeneity; identified country trajectory groups using dynamic time warping–based clustering; and decomposed changes in counts into contributions from population growth, population aging, and changes in age-specific outcome rates. We also explored whether GBD-estimated rates during 2020–2023 deviated from trajectories extrapolated from 2010 - 2019, without attributing such deviations causally to the COVID-19 pandemic, and projected age-standardized rates to 2030 under alternative modeling windows. Because the analysis did not include country-level measurements of smoking, schistosomiasis, occupational exposure, or health-system capacity, these factors are considered contextual explanations rather than causal mechanisms tested in the present study.

## Methods

### Study design and data source

We conducted an ecological secondary analysis of modeled population-level estimates from the Global Burden of Diseases, Injuries, and Risk Factors Study 2023 (GBD 2023) to characterize bladder cancer burden across Africa. Annual estimates from 2010 through 2023 were analyzed, and study-specific projections were generated through 2030. The endpoint of 2023 was selected because it was the latest year available in GBD 2023. The starting year of 2010 was chosen to focus on contemporary burden patterns while providing 10 annual prepandemic estimates (2010–2019) and four pandemic-era estimates (2020–2023) for the exploratory time-series analysis.

We extracted estimates for bladder cancer incidence, prevalence, deaths, and disability-adjusted life-years (DALYs), including all-age counts and age-standardized rates (ASRs) per 100,000 population for both sexes combined. Corresponding GBD population estimates were used in the decomposition analysis. The continent-level analysis used the aggregate location labeled “African Union” in the GBD dataset; this series was obtained directly from GBD and was not calculated by averaging country estimates.

GBD 2023 synthesizes population-based cancer-registry, vital-registration, and verbal-autopsy data using standardized statistical models. Cancer mortality is estimated using Cause of Death Ensemble modeling and mortality-to-incidence ratios, from which incidence is derived. Prevalence is modeled using incidence and survival, while DALYs combine years lived with disability and years of life lost [14]. Accordingly, the analyzed values are GBD-modeled estimates rather than directly reported national registry data

### Geographic scope and outcomes

Country-level analyses included 54 prespecified African countries: Algeria, Angola, Benin, Botswana, Burkina Faso, Burundi, Cabo Verde, Cameroon, Central African Republic, Chad, Comoros, Congo, Côte d’Ivoire, Democratic Republic of the Congo, Djibouti, Egypt, Equatorial Guinea, Eritrea, Eswatini, Ethiopia, Gabon, Gambia, Ghana, Guinea, Guinea-Bissau, Kenya, Lesotho, Liberia, Libya, Madagascar, Malawi, Mali, Mauritania, Mauritius, Morocco, Mozambique, Namibia, Niger, Nigeria, Rwanda, Sao Tome and Principe, Senegal, Seychelles, Sierra Leone, Somalia, South Africa, South Sudan, Sudan, Tanzania, Togo, Tunisia, Uganda, Zambia, and Zimbabwe. No separate country-level estimate was analyzed for Western Sahara/Sahrawi Arab Democratic Republic.

For presentation, countries were grouped into North Africa and Central, Eastern, Southern, and Western sub-Saharan Africa. These groupings were used only to organize descriptive comparisons and were not explanatory variables in the statistical models. The study outcomes were incidence, prevalence, deaths, and DALYs. For each outcome, we analyzed counts and ASRs, with GBD 95% uncertainty intervals (UIs) reported for descriptive estimates. Downstream analyses used GBD mean point estimates; draw-level uncertainty was not propagated through the secondary models.

### Descriptive and trend analyses

We summarized continent- and country-level burden in 2010 and 2023. Country-specific ASRs were displayed using choropleth maps. For each outcome and year separately, countries were grouped into up to five categories based on empirical quintiles of the ASR distribution. Because category boundaries were year-specific, the maps were interpreted as showing relative geographic patterns within each year rather than absolute temporal changes based on color categories alone.

Temporal trends were summarized using the estimated annual percentage change (EAPC). For each country and outcome, we fitted:

$$\ln \left( {AS{R_y}} \right) = \alpha + \beta y + {\varepsilon _y}$$

and calculated:

$$EAPC = 100\left\{ {\exp \left( \beta \right) - 1} \right\}$$

The 95% confidence interval (CI) was obtained by transforming the corresponding confidence limits for β [15]. A trend was classified as increasing when the lower CI limit was greater than zero, decreasing when the upper limit was less than zero, and showing no clear change when the CI included zero.

Trajectory clustering

Dynamic time warping (DTW)–based partitional clustering was used to identify descriptive groups of country-level incidence trajectories. Annual incidence ASRs from 2010 through 2023 were analyzed, and only countries with complete 14-year series were eligible. Clustering was performed on the original ASR scale without within-country standardization; therefore, the groups reflected both incidence level and trajectory shape. We used dtw_basic distance, DTW barycenter averaging, three prespecified clusters, and a random seed of 20,260,307. The three-cluster solution was treated as a descriptive summary rather than a statistically unique or etiologically defined classification. Groups were ordered by their mean 2023 ASR and labeled lower-, intermediate-, and higher-burden trajectory groups.

### Decomposition analysis

A symmetric Das Gupta decomposition was used to partition changes in counts between 2010 and 2023 into contributions from total population size, population age structure, and age-specific rates. Age-specific population and outcome-rate files were matched by country, year, and harmonized age group. For country c and year t, the reconstructed count was:

$${N_{c,t}} = \mathop \sum \limits_a {{{P_{c,t}}{A_{c,a,t}}{R_{c,a,t}}} \over {100000}}$$

where P denotes total population, A the age-specific population proportion, and R the age-specific outcome rate. Contributions were calculated for all six possible replacement orders of the three components and averaged across orders. Country-level contributions were summed to obtain the 54-country continental summary. The age-specific rate-change component is a mathematical component and was not interpreted as a causal effect; it may reflect changes in exposure, detection, registration, treatment, survival, or other factors.\

### Exploratory analysis of pandemic-era deviations

We conducted an exploratory, non-causal analysis of whether GBD-estimated ASRs during 2020–2023 deviated from trajectories extrapolated from 2010 to 2019. For each outcome, the 10 prepandemic ASRs for the GBD aggregate labeled “African Union” were log-transformed, and an autoregressive integrated moving average model was selected using auto.arima(), with stepwise search and approximation disabled. The model generated forecasts and pointwise 95% prediction intervals for 2020–2023, which were back-transformed by exponentiation. Residual autocorrelation was assessed using the Ljung–Box test at lag 9.

Annual deviations were calculated as the GBD-estimated ASR minus the forecasted ASR. Positive and negative deviations were summed separately across 2020–2023. These summaries have units of ASR-years per 100,000 and do not represent excess cases, deaths, or DALYs. Given the short training series and modeled input estimates, this analysis does not establish a causal effect of the COVID-19 pandemic.

### Projections through 2030

Country-specific ASRs were projected using ordinary least-squares log-linear models:

$$ln\left( {ASRt} \right) = \alpha + \beta t + \varepsilon t.$$

The primary model used 2010–2023 data; sensitivity models used 2010–2019 and 2010–2018. Parameter uncertainty was assessed using 2,000 coefficient vectors sampled from a multivariate normal distribution defined by the fitted coefficients and variance–covariance matrix. The random seed was 20,260,307. The 2.5th and 97.5th percentiles of the back-transformed simulated values defined coefficient-based 95% CIs.

Residual innovations and GBD draw-level uncertainty were not included; these intervals should therefore not be interpreted as prediction intervals or GBD UIs. The model-fitted 2023 ASR was used as the within-model reference for change through 2030. Projections were limited to ASRs and did not incorporate future population changes, risk-factor trajectories, diagnostic capacity, or interventions.

Statistical software and ethics

Analyses were performed in R using dplyr, ggplot2, broom, sf, dtwclust, forecast, and MASS. The study used publicly available, deidentified aggregate estimates and did not involve individual-level data; institutional review board approval and informed consent were therefore not applicable.

## Results

### Burden and geographic distribution in 2010 and 2023

For the GBD aggregate labeled “African Union,” all four measures of bladder cancer burden increased between 2010 and 2023 (Table 1). Incident cases increased from 16,357 (95% uncertainty interval [UI], 12,903– 21,419) to 31,503 (23,135– 42,624), prevalent cases from 73,806 (55,965– 96,924) to 147,599 (106,679–205,827), deaths from 9,261 (7,146– 12,166) to 16,467 (12,059– 23,280), and disability-adjusted life-years (DALYs) from 232,414 (180,649–302,490) to 415,746 (306,075–581,236). The corresponding age-standardized rates (ASRs) per 100,000 population increased from 3.5 (95% UI, 2.7–4.6) to 4.4 (3.2–6.0) for incidence, from 13.9 (10.6–18.1) to 18.3 (13.2–25.2) for prevalence, from 2.2 (1.7–2.9) to 2.6 (1.9–3.6) for deaths, and from 48.1 (37.4–63.0) to 56.1 (41.5–78.8) for DALYs. The estimated annual percentage changes (EAPCs) were 1.42% (95% confidence interval [CI], 0.99%–1.86%) for incidence, 1.54% (0.97%–2.12%) for prevalence, 0.89% (0.52%–1.26%) for deaths, and 0.84% (0.43%–1.24%) for DALYs.

**Table 1:** Bladder cancer burden in the African Union and 54 African countries in 2010 and 2023. This table presents the number of cases and age-standardized rates (ASRs) of bladder cancer incidence, prevalence, deaths, and disability-adjusted life-years (DALYs) in the African Union and 54 African countries in 2010 and 2023, together with the estimated annual percentage change (EAPC) in ASRs from 2010 to 2023. Estimates are shown with 95% uncertainty intervals and are based on both sexes combined. Countries are grouped by African region. ASR indicates age-standardized rate; DALYs, disability-adjusted life-years; and EAPC, estimated annual percentage change

Region	Location	Number_2010	ASR_2010	Number_2023	ASR_2023	EAPC_95CI
African Union	African Union	9261 (7146–12166)	2.2 (1.7–2.9)	16467 (12059–23280)	2.6 (1.9–3.6)	0.89 (0.52–1.26)
North Africa	Algeria	353 (241–520)	1.7 (1.1–2.5)	663 (384–1033)	1.9 (1.1–3.0)	1.02 (0.80–1.23)
North Africa	Egypt	1604 (1231–2126)	4.5 (3.5–6.1)	2670 (1862–3726)	4.8 (3.4–6.8)	−0.09 (−1.01–0.85)
North Africa	Libya	165 (112–235)	5.3 (3.6–7.6)	257 (160–387)	5.3 (3.3–7.9)	−0.19 (−0.71–0.33)
North Africa	Morocco	276 (176–413)	1.5 (0.9–2.2)	574 (348–927)	1.9 (1.1–3.0)	1.82 (1.54–2.10)
North Africa	Sudan	360 (244–552)	2.8 (1.9–4.2)	577 (363–900)	3.0 (1.9–4.8)	0.60 (0.25–0.95)
North Africa	Tunisia	394 (281–545)	4.8 (3.4–6.6)	619 (451–839)	4.9 (3.6–6.6)	−0.70 (−1.33–0.06)
Central Sub-Saharan Africa	Angola	140 (99–192)	2.2 (1.6–3.0)	267 (190–367)	2.4 (1.7–3.2)	0.10 (−0.32–0.53)
Central Sub-Saharan Africa	Cameroon	176 (126–257)	2.5 (1.8–3.7)	320 (210–466)	2.9 (1.9–4.2)	0.70 (0.40–1.00)
Central Sub-Saharan Africa	Central African Republic	20 (14–27)	1.3 (1.0–1.9)	35 (24–51)	1.6 (1.1–2.4)	1.10 (0.48–1.72)
Central Sub-Saharan Africa	Chad	76 (49–109)	2.1 (1.4–3.0)	130 (86–194)	2.5 (1.7–3.8)	0.88 (0.38–1.37)
Central Sub-Saharan Africa	Congo	34 (25–47)	2.2 (1.6–3.1)	65 (46–92)	3.0 (2.1–4.2)	2.07 (1.65–2.50)
Central Sub-Saharan Africa	Democratic Republic of the Congo	396 (249–567)	1.8 (1.1–2.6)	857 (566–1223)	2.5 (1.6–3.7)	2.65 (2.06–3.25)
Central Sub-Saharan Africa	Equatorial Guinea	9 (6–13)	3.2 (2.2–4.7)	18 (12–25)	3.9 (2.7–5.5)	1.29 (0.77–1.82)
Central Sub-Saharan Africa	Gabon	18 (12–25)	2.2 (1.5–3.1)	34 (23–49)	3.0 (2.0–4.3)	1.91 (1.44–2.39)
Central Sub-Saharan Africa	Sao Tome and Principe	4 (3–5)	4.7 (3.3–6.6)	6 (3–8)	5.1 (3.1–7.2)	1.06 (0.54–1.58)
Eastern Sub-Saharan Africa	Burundi	57 (39–82)	1.9 (1.3–2.8)	107 (69–155)	2.3 (1.5–3.3)	1.15 (0.70–1.60)
Eastern Sub-Saharan Africa	Comoros	4 (3–5)	0.9 (0.6–1.2)	6 (4–9)	1.0 (0.7–1.5)	1.34 (0.43–2.26)
Eastern Sub-Saharan Africa	Djibouti	6 (4–9)	1.9 (1.3–2.6)	15 (10–22)	2.2 (1.4–3.3)	1.13 (0.84–1.42)
Eastern Sub-Saharan Africa	Eritrea	37 (28–49)	1.6 (1.2–2.2)	73 (51–99)	2.1 (1.5–2.9)	1.94 (1.34–2.55)
Eastern Sub-Saharan Africa	Ethiopia	664 (371–987)	2.3 (1.3–3.5)	1487 (926–2282)	3.4 (2.1–5.2)	2.56 (2.12–3.00)
Eastern Sub-Saharan Africa	Kenya	138 (102–187)	0.9 (0.7–1.3)	251 (186–339)	1.0 (0.8–1.4)	1.24 (0.35–2.14)
Eastern Sub-Saharan Africa	Madagascar	202 (146–278)	3.0 (2.1–4.2)	367 (257–511)	3.6 (2.5–5.1)	1.24 (0.60–1.88)
Eastern Sub-Saharan Africa	Malawi	271 (208–352)	5.2 (4.0–6.8)	523 (370–703)	6.5 (4.6–8.8)	2.10 (0.76–3.46)
Eastern Sub-Saharan Africa	Mauritius	26 (23–30)	2.2 (2.0–2.5)	37 (32–43)	2.1 (1.8–2.4)	−1.27 (−1.79–0.74)
Eastern Sub-Saharan Africa	Mozambique	182 (137–231)	2.1 (1.6–2.6)	265 (182–369)	2.2 (1.5–3.1)	0.68 (0.13–1.23)
Eastern Sub-Saharan Africa	Rwanda	112 (80–153)	3.0 (2.2–4.2)	246 (166–341)	3.7 (2.5–5.1)	1.12 (0.55–1.69)
Eastern Sub-Saharan Africa	Seychelles	4 (3–4)	4.3 (3.4–5.4)	5 (3–6)	4.0 (3.0–5.1)	−1.47 (−2.18–0.75)
Eastern Sub-Saharan Africa	Somalia	42 (24–61)	0.9 (0.5–1.2)	86 (55–126)	1.0 (0.7–1.5)	1.84 (0.87–2.81)
Eastern Sub-Saharan Africa	South Sudan	75 (54–104)	2.6 (1.9–3.7)	114 (80–159)	3.0 (2.1–4.1)	0.47 (−0.01–0.96)
Eastern Sub-Saharan Africa	Uganda	211 (159–276)	2.2 (1.6–2.8)	375 (253–517)	2.4 (1.6–3.4)	0.66 (0.05–1.27)
Eastern Sub-Saharan Africa	United Republic of Tanzania	456 (336–597)	2.5 (1.8–3.3)	685 (505–917)	2.5 (1.8–3.3)	−0.04 (−0.61–0.54)
Eastern Sub-Saharan Africa	Zambia	116 (88–150)	3.1 (2.3–4.0)	235 (161–313)	3.8 (2.6–5.1)	1.75 (1.19–2.32)
Southern Sub-Saharan Africa	Botswana	4 (3–5)	0.3 (0.3–0.5)	8 (5–12)	0.5 (0.3–0.7)	2.86 (2.38–3.35)
Southern Sub-Saharan Africa	Eswatini	4 (3–5)	0.7 (0.5–1.0)	5 (4–7)	0.7 (0.5–1.0)	0.21 (−1.00–1.44)
Southern Sub-Saharan Africa	Lesotho	10 (8–14)	1.0 (0.8–1.4)	17 (13–25)	1.4 (1.0–2.0)	1.60 (0.79–2.41)
Southern Sub-Saharan Africa	Namibia	10 (8–13)	0.9 (0.7–1.2)	17 (12–23)	1.0 (0.7–1.4)	0.93 (0.37–1.49)
Southern Sub-Saharan Africa	South Africa	708 (592–865)	2.1 (1.8–2.6)	1087 (851–1377)	2.2 (1.8–2.8)	−0.57 (−1.43–0.30)
Southern Sub-Saharan Africa	Zimbabwe	173 (131–234)	2.9 (2.2–3.9)	250 (176–346)	3.0 (2.1–4.1)	0.48 (−0.37–1.34)
Western Sub-Saharan Africa	Benin	30 (22–45)	0.9 (0.7–1.4)	50 (33–75)	0.9 (0.6–1.4)	−0.13 (−0.38–0.13)
Western Sub-Saharan Africa	Burkina Faso	105 (63–151)	1.7 (1.0–2.5)	183 (118–263)	2.0 (1.3–2.9)	0.74 (0.09–1.39)
Western Sub-Saharan Africa	Cabo Verde	6 (4–9)	1.7 (1.1–2.4)	8 (5–11)	1.7 (1.1–2.5)	0.00 (−0.41–0.41)
Western Sub-Saharan Africa	Côte d’Ivoire	95 (67–133)	1.5 (1.0–2.1)	230 (153–341)	2.1 (1.4–3.1)	2.05 (1.40–2.69)
Western Sub-Saharan Africa	Gambia	4 (3–6)	0.6 (0.5–0.8)	8 (6–12)	0.8 (0.6–1.1)	3.20 (2.44–3.96)
Western Sub-Saharan Africa	Ghana	214 (148–298)	1.9 (1.3–2.7)	434 (282–617)	2.6 (1.7–3.8)	2.21 (1.90–2.53)
Western Sub-Saharan Africa	Guinea	74 (53–102)	1.6 (1.1–2.2)	109 (75–161)	1.9 (1.3–2.9)	1.28 (0.73–1.84)
Western Sub-Saharan Africa	Guinea-Bissau	8 (6–12)	1.7 (1.2–2.4)	14 (9–21)	2.1 (1.4–3.0)	1.45 (1.00–1.90)
Western Sub-Saharan Africa	Liberia	33 (23–48)	2.3 (1.6–3.3)	56 (38–82)	2.6 (1.7–3.8)	0.67 (−0.02–1.37)
Western Sub-Saharan Africa	Mali	276 (202–383)	5.1 (3.7–7.2)	516 (376–701)	6.0 (4.4–8.2)	1.14 (0.60–1.68)
Western Sub-Saharan Africa	Mauritania	32 (23–46)	2.3 (1.6–3.3)	55 (34–80)	2.7 (1.7–3.9)	1.11 (0.30–1.93)
Western Sub-Saharan Africa	Niger	75 (55–108)	1.9 (1.4–2.7)	144 (99–215)	2.1 (1.4–3.0)	0.84 (0.56–1.13)
Western Sub-Saharan Africa	Nigeria	606 (420–830)	1.1 (0.7–1.5)	1005 (651–1460)	1.2 (0.8–1.8)	0.78 (0.48–1.07)
Western Sub-Saharan Africa	Senegal	81 (56–114)	1.5 (1.0–2.1)	146 (97–208)	1.8 (1.2–2.6)	0.94 (0.23–1.66)
Western Sub-Saharan Africa	Sierra Leone	53 (37–75)	2.0 (1.4–2.8)	81 (54–116)	2.5 (1.7–3.7)	1.59 (1.23–1.95)
Western Sub-Saharan Africa	Togo	34 (25–46)	1.6 (1.2–2.2)	73 (50–103)	2.0 (1.4–2.8)	1.38 (0.75–2.01)

**Fig. 1:**
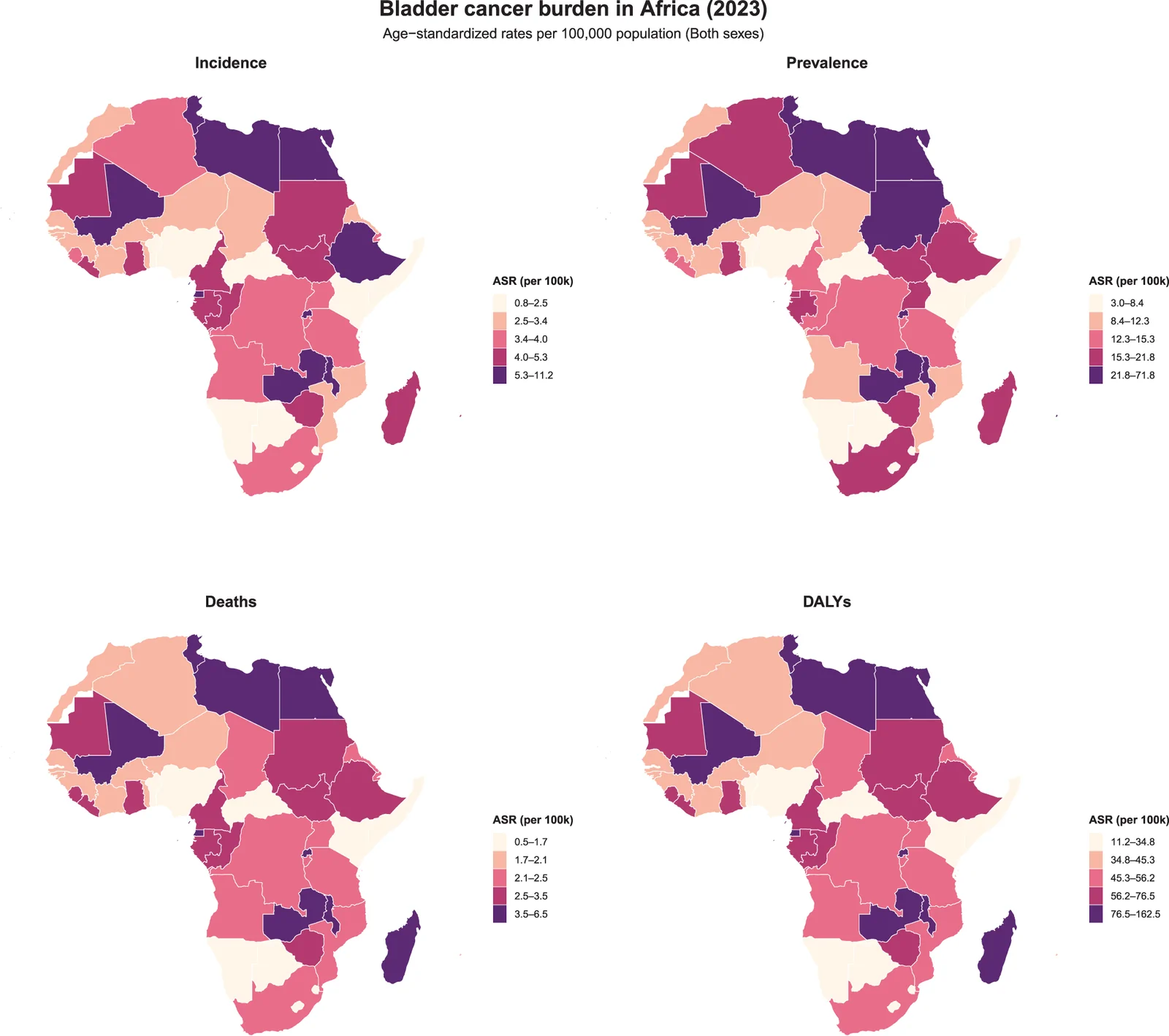
Geographic distribution of bladder cancer burden in Africa in 2023. Maps show the age-standardized incidence, prevalence, death, and disability-adjusted life-year (DALY) rates of bladder cancer per 100 000 population across 54 African countries in 2023 for both sexes combined. Countries were grouped into 5 categories for each outcome according to the distribution of country-specific age-standardized rates. DALYs indicate disability-adjusted life-years

Country-level estimates showed marked heterogeneity. In 2023, the highest incidence ASRs were estimated in Tunisia (11.2 per 100,000), Malawi (10.6), Libya (10.5), Egypt (9.2), and Mali (9.0), whereas the lowest were in Botswana (0.8), Eswatini (1.2), Gambia (1.2), Somalia (1.4), and Benin (1.5). Prevalence ASRs were highest in Tunisia (71.8), Libya (49.9), Seychelles (43.6), Egypt (40.2), and Malawi (40.1), and lowest in Botswana (3.0), Gambia (4.5), Somalia (5.3), Benin (5.4), and Eswatini (6.1). Malawi had the highest death ASR (6.5) and DALY ASR (162.5), followed by Mali for both outcomes (6.0 and 133.7, respectively); Botswana had the lowest death and DALY ASRs (0.5 and 11.2, respectively). Within each mapped year, higher ASR categories were most frequent in North Africa but also occurred in selected countries in Eastern, Central, Southern, and Western Africa; many Western and Central African countries remained in lower categories (Fig. 1 and Supplementary Figure 1). Because the map categories were based on year-specific quintiles, their colors indicate relative position within each year rather than absolute change between 2010 and 2023.

### Temporal trends in age-standardized burden across 54 countries

**Fig. 2:**
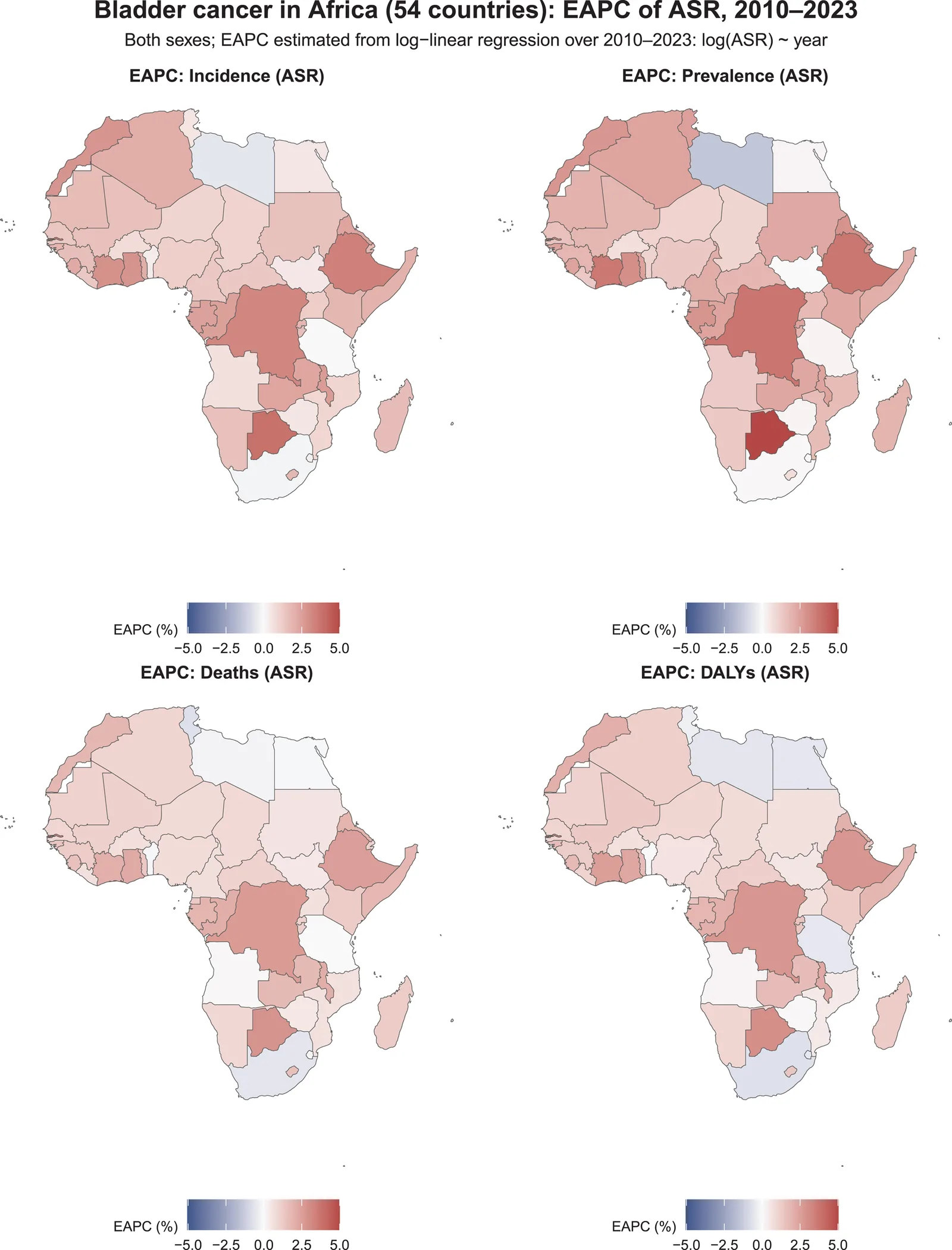
Estimated annual percentage change in age-standardized bladder cancer burden across 54 African countries, 2010–2023. Maps show the estimated annual percentage change (EAPC) in age-standardized incidence, prevalence, death, and DALY rates of bladder cancer across 54 African countries from 2010 to 2023 for both sexes combined. EAPCs were derived from log-linear regression models fitted to country-specific age-standardized rates over time. Positive values indicate increasing trends and negative values indicate decreasing trends. ASR indicates age-standardized rate; DALYs, disability-adjusted life-years; and EAPC, estimated annual percentage change

Using the prespecified CI-based classification, incidence ASRs increased in 44 countries, decreased in 2, and showed no clear change in 8 (Supplementary Table 1 and Fig. 2). The largest incidence EAPCs occurred in Botswana (3.91%; 95% CI, 3.45%–4.37%), Gambia (3.41%; 2.67%–4.17%), Ethiopia (3.40%; 2.92%–3.89%), the Democratic Republic of the Congo (3.26%; 2.67%–3.86%), and Côte d’Ivoire (2.89%; 2.27%–3.51%). Incidence decreased in Mauritius (−0.93%; −1.43% to −0.42%) and Seychelles (−0.89%; −1.73% to −0.04%).

Prevalence ASRs increased in 45 countries, decreased in 2, and showed no clear change in 7. The largest prevalence EAPCs were in Botswana (5.02%; 95% CI, 4.76%–5.28%), the Democratic Republic of the Congo (3.75%; 3.24%–4.26%), Ethiopia (3.70%; 3.10%–4.31%), Côte d’Ivoire (3.66%; 3.15%–4.16%), and Gambia (3.09%; 2.44%–3.74%). Decreases occurred in Libya (−1.42%; −2.03% to −0.81%) and Mauritius (−0.60%; −1.07% to −0.12%). Death ASRs increased in 40 countries, decreased in 3, and showed no clear change in 11; corresponding numbers for DALY ASRs were 41, 3, and 10. Gambia had the largest EAPCs for both deaths (3.20%; 2.44%–3.96%) and DALYs (3.25%; 2.50%–4.01%). Death ASRs decreased in Seychelles, Mauritius, and Tunisia, whereas DALY ASRs decreased in Seychelles, Mauritius, and Libya.

After Gambia, the largest death EAPCs were in Botswana (2.86%; 95% CI, 2.38%–3.35%), the Democratic Republic of the Congo (2.65%; 2.06%–3.25%), and Ethiopia (2.56%; 2.12%–3.00%). The next largest DALY EAPCs were likewise in Botswana (2.96%; 2.53%–3.39%), the Democratic Republic of the Congo (2.77%; 2.26%–3.28%), and Ethiopia (2.77%; 2.30%–3.24%). Geographically, stronger increases were distributed across parts of Eastern, Western, and Central Africa, while weaker or declining trends occurred in several island, Southern African, and North African locations (Fig. 2).

### Descriptive clustering of incidence trajectories

**Fig. 3:**
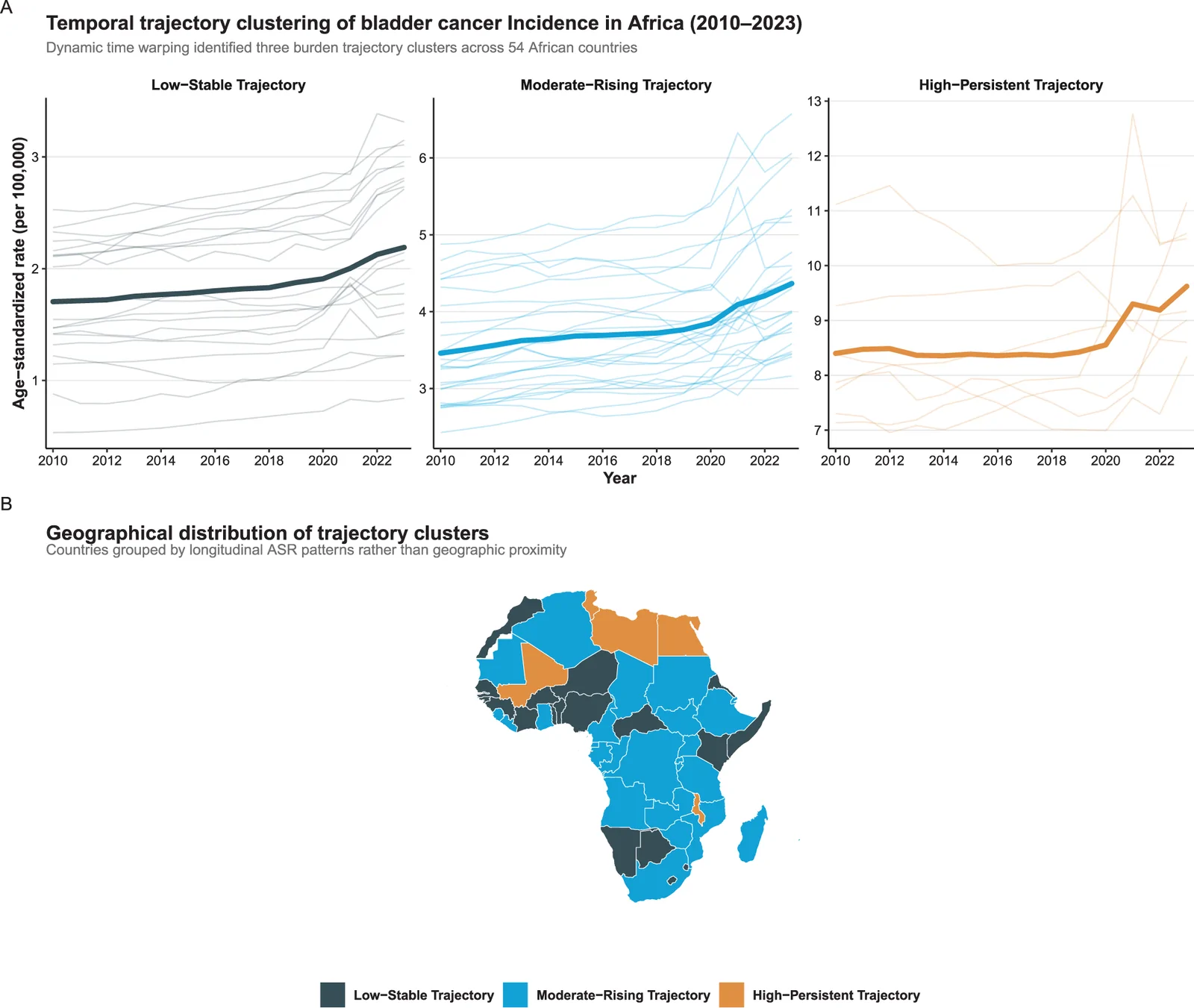
Temporal trajectory clustering of bladder cancer incidence in Africa, 2010–2023. Panel a shows dynamic time warping-based clustering of country-level age-standardized incidence rate trajectories for bladder cancer across 54 African countries from 2010 to 2023, classifying countries into low-stable, moderate-rising, and high-persistent trajectory groups. Thin lines represent individual countries and bold lines represent cluster centroids. Panel B shows the geographic distribution of the 3 trajectory clusters. Rates are presented per 100 000 population and for both sexes combined

All 54 countries had complete annual incidence ASR series for 2010–2023 and were included in the dynamic time warping analysis. When the three prespecified groups were ordered by mean 2023 ASR, 20 countries were assigned to the lower-burden trajectory group, 27 to the intermediate-burden group, and 7 to the higher-burden group (Supplementary Table 2 and Fig. 3). The arithmetic mean trajectories remained separated across most of the study period: the lower-burden group remained at comparatively low incidence levels, the intermediate-burden group occupied the middle range, and the higher-burden group remained at the greatest levels.

The geographic map showed that none of the groups was confined to a single subregion. The higher-burden group included countries from North, Eastern, and Western Africa, while the lower- and intermediate-burden groups were distributed more broadly across sub-Saharan Africa. Because the analysis used original, unstandardized ASRs, the resulting groups reflected both burden level and temporal shape rather than shape alone.

### Decomposition of changes in counts

**Fig. 4:**
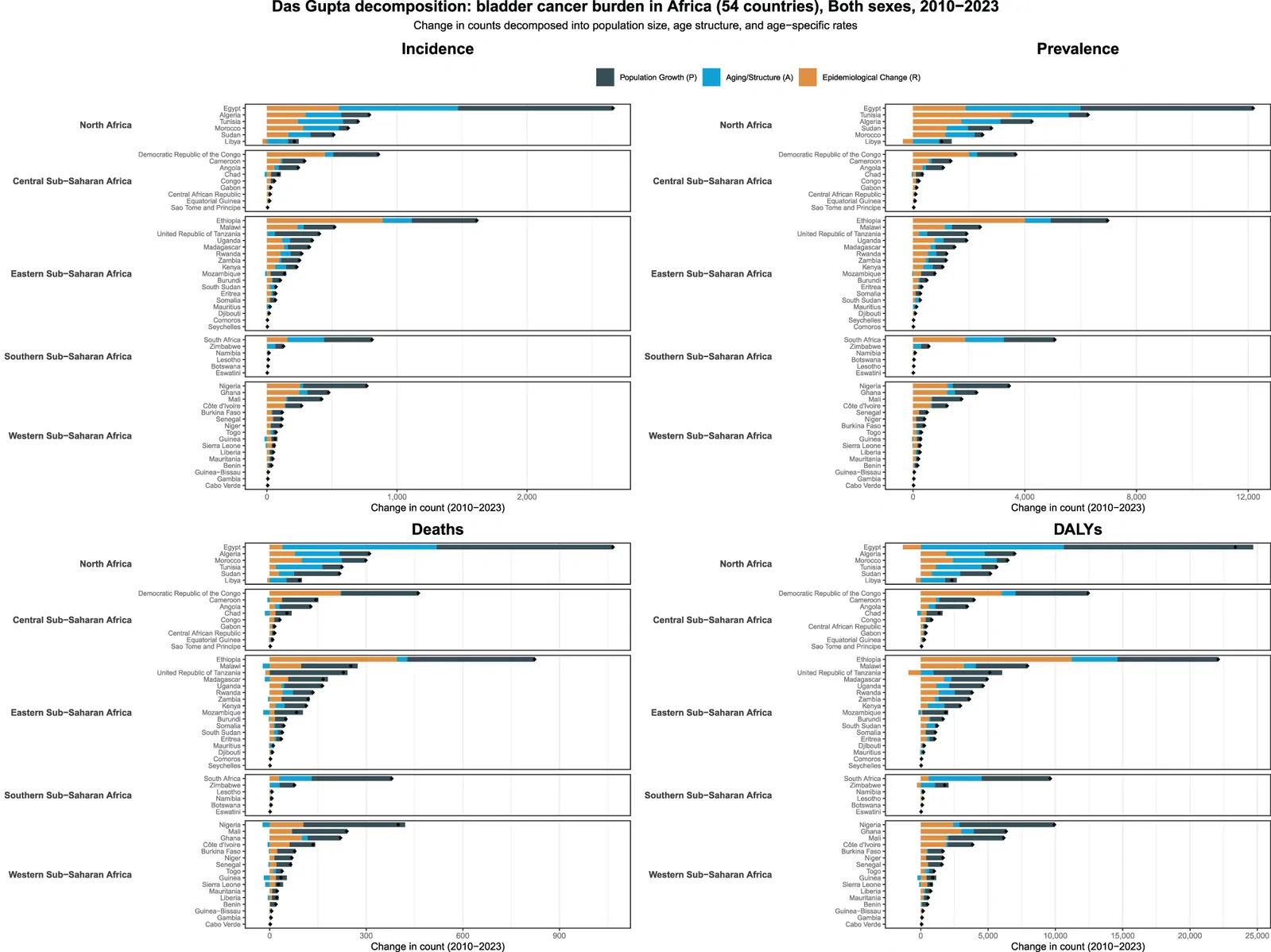
Das Gupta decomposition of changes in bladder cancer burden across 54 African countries, 2010–2023. Stacked bar plots show the decomposition of changes in bladder cancer incidence, prevalence, deaths, and DALYs between 2010 and 2023 into contributions from population growth, population aging or age structure change, and epidemiological change in age-specific rates across 54 African countries. Black diamonds indicate the net absolute change in counts for each country. Countries are grouped by African region and ranked within region according to the magnitude of overall change. Analyses were based on both sexes combined. DALYs indicate disability-adjusted life-years

Across the 54 countries, the reconstructed increases between 2010 and 2023 were 15,146 incident cases, 73,793 prevalent cases, 7,205 deaths, and 183,332 DALYs (Supplementary Table 3 and Fig. 4). Population-size change accounted for 43.2% of the increase in incidence, 39.8% in prevalence, 58.8% in deaths, and 50.3% in DALYs. Age-structure change accounted for 22.5%, 22.1%, 15.9%, and 23.8%, respectively, while the age-specific rate-change component accounted for 34.4%, 38.1%, 25.2%, and 25.9%, respectively.

North Africa contributed the largest absolute increases in incidence (5,498 reconstructed cases) and prevalence (29,009), whereas Eastern sub-Saharan Africa contributed the largest increases in deaths (2,277) and DALYs (62,455). At the country level, Egypt had the largest absolute increases in incidence (2,661 reconstructed cases), prevalence (12,178), deaths (1,066), and DALYs (23,368); Ethiopia ranked second for all four measures. The Democratic Republic of the Congo, Nigeria, and South Africa also contributed large absolute increases, although their ordering differed by outcome.

The age-specific rate-change component was negative in 4 countries for incidence, 2 for prevalence, 5 for deaths, and 7 for DALYs. For incidence, negative components occurred in Libya, Mauritius, Seychelles, and Eswatini; for prevalence, they occurred in Libya and Eswatini. The component was negative for deaths in Libya, Tanzania, Mauritius, Seychelles, and Eswatini and for DALYs in Egypt, Libya, Tanzania, Mauritius, Seychelles, Zimbabwe, and Eswatini. Thus, it was negative for all four outcomes in Libya and Eswatini. These negative components offset part of the positive demographic contribution but do not identify the underlying mechanism.

### Exploratory pandemic-era deviations from extrapolated trends

**Fig. 5:**
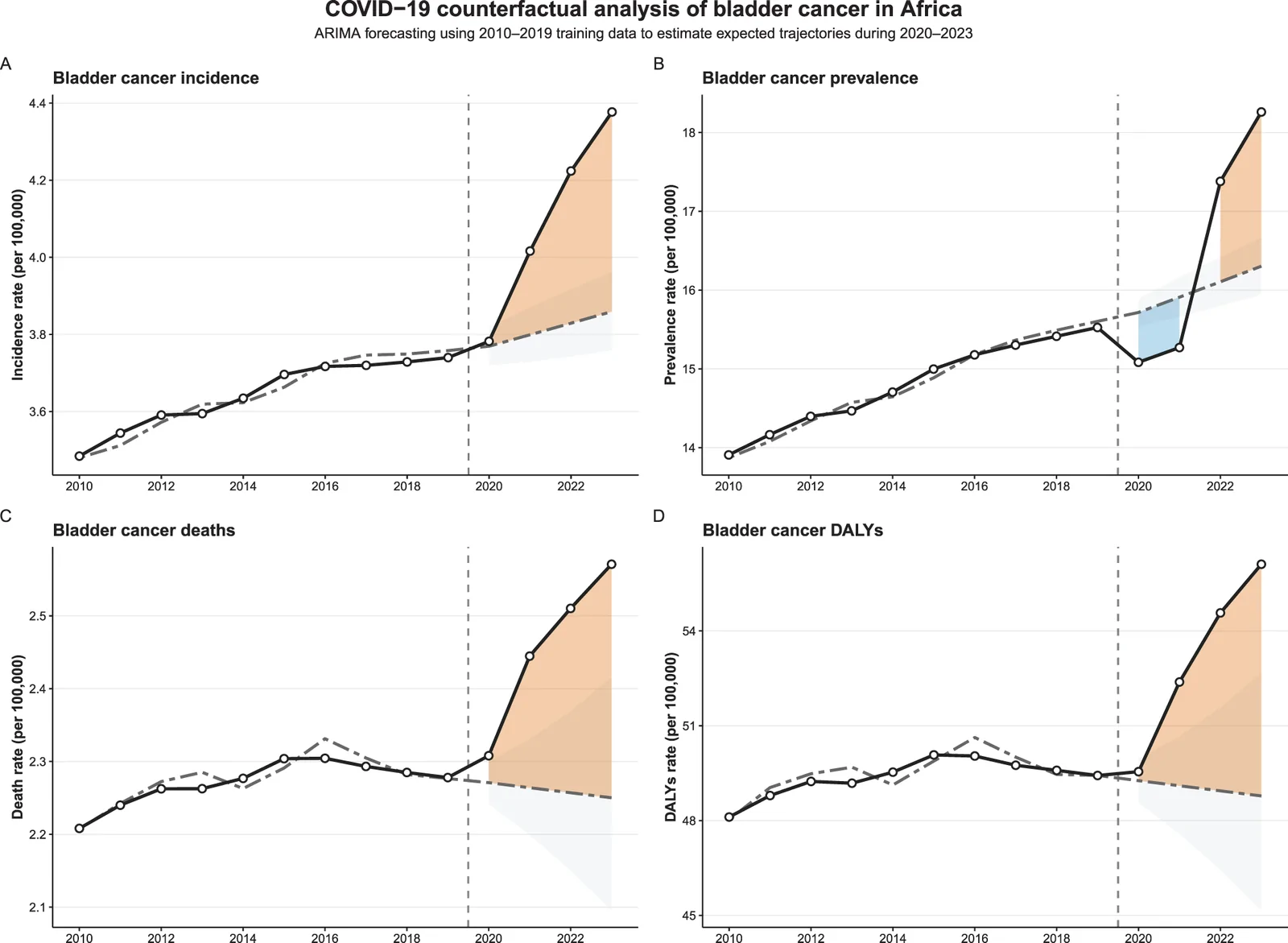
Counterfactual analysis of bladder cancer burden during the COVID-19 period in Africa. Panels a to D compare observed age-standardized incidence, prevalence, death, and DALY rates of bladder cancer with counterfactual expected trajectories estimated from autoregressive integrated moving average models trained on prepandemic data from 2010 to 2019. The vertical dashed line marks the beginning of the pandemic period. Shaded areas represent the gap between observed and expected values during 2020 to 2023 and the 95% prediction intervals around model-based expectations. Rates are shown per 100 000 population for both sexes combined. DALYs indicate disability-adjusted life-years

For the GBD aggregate labeled “African Union,” rates estimated for 2020–2023 were compared with point forecasts from ARIMA models fitted to the 2010–2019 series (Supplementary Table 4 and Fig. 5). Across these four years, the mean GBD-estimated versus forecasted ASRs were 4.10 versus 3.81 per 100,000 for incidence, 2.46 versus 2.26 for deaths, and 53.15 versus 49.02 for DALYs. The sums of positive annual deviations were 1.14, 0.79, and 16.53 ASR-years per 100,000 for incidence, deaths, and DALYs, respectively.

The incidence ASR was close to its point forecast in 2020 (3.78 versus 3.77 per 100,000) and was further above it in 2023 (4.38 versus 3.86). The corresponding GBD-estimated and forecasted values were 2.31 versus 2.27 in 2020 and 2.57 versus 2.25 in 2023 for deaths, and 49.55 versus 49.26 in 2020 and 56.11 versus 48.78 in 2023 for DALYs. Prevalence was below its forecast in 2020 and 2021 and above it in 2022 and 2023. Across the four years, its mean GBD-estimated ASR was 16.50 versus a forecasted 16.01; the summed positive and negative annual deviations were 3.23 and 1.27 ASR-years per 100,000, respectively. These quantities summarize differences between modeled GBD rates and extrapolated rate trajectories; they are not estimates of excess cases, deaths, or DALYs.

### Projections through 2030

**Fig. 6:**
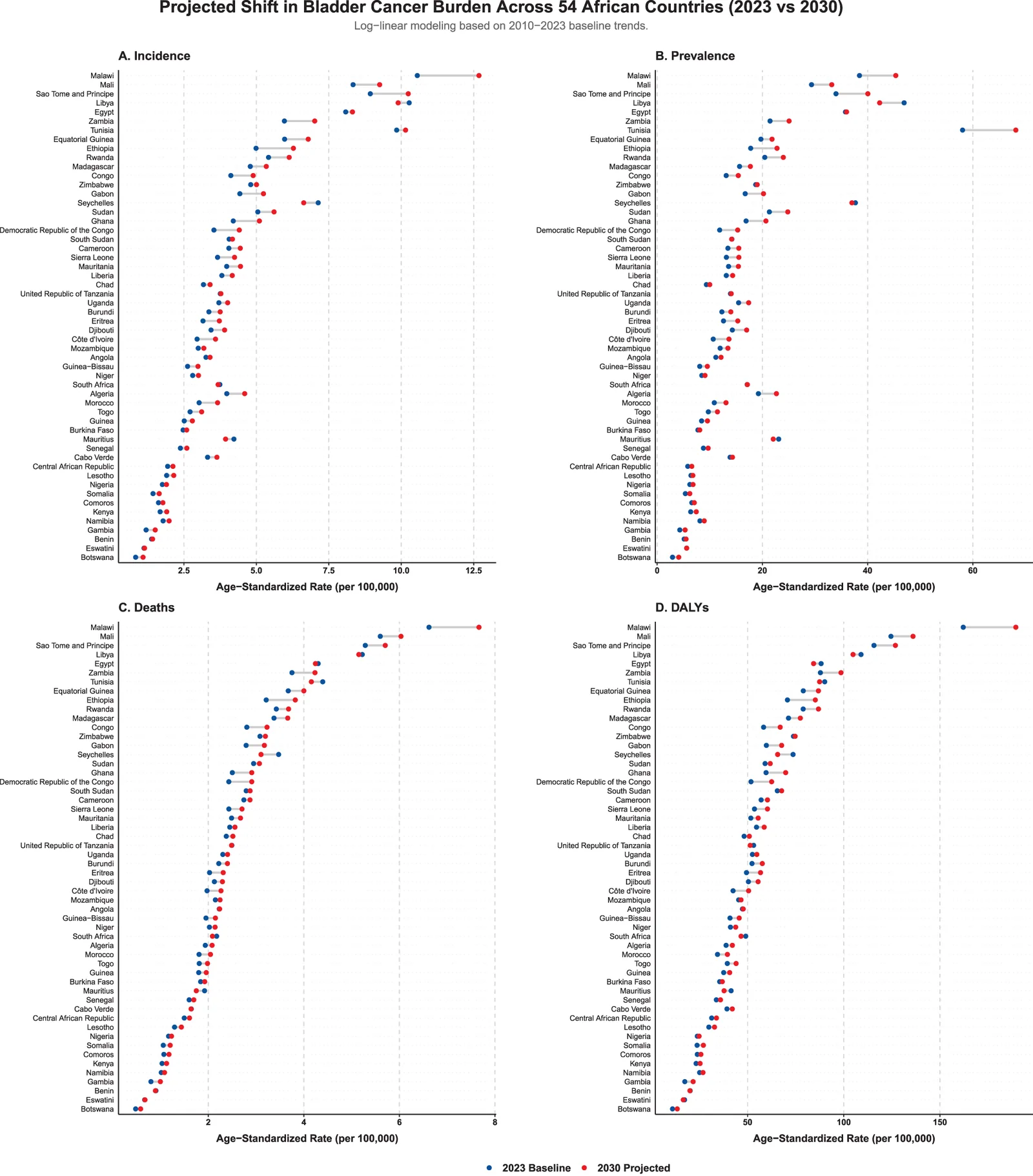
Projected shift in bladder cancer burden across 54 African countries from 2023 to 2030. Panels a to D show country-specific age-standardized incidence, prevalence, death, and disability-adjusted life-year (DALY) rates of bladder cancer in 2023 and the projected rates for 2030 across 54 African countries. Blue dots indicate observed 2023 baseline estimates and red dots indicate projected 2030 estimates derived from log-linear models based on 2010 to 2023 trends. Countries are grouped by African region and ordered according to the magnitude of age-standardized rates. Rates are presented per 100 000 population. DALYs indicate disability-adjusted life-years

In the primary log-linear models fitted to 2010–2023 data, the projected 2030 ASR was higher than the model-fitted 2023 reference in 50 countries for incidence and prevalence and in 45 countries for deaths and DALYs (Supplementary Table 5 and Fig. 6). The highest projected incidence ASRs in 2030 were in Malawi (12.68 per 100,000), Sao Tome and Principe (10.24), Tunisia (10.15), Libya (9.89), and Mali (9.25). Incidence trajectories decreased in four countries: Seychelles, Libya, Mauritius, and South Africa. For prevalence, the highest projected rates were in Tunisia (68.17), Malawi (45.35), Libya (42.29), Sao Tome and Principe (40.04), and Seychelles (37.02); fitted trajectories decreased in Libya, Mauritius, Seychelles, and South Sudan.

The highest projected death ASRs were in Malawi (7.66 per 100,000), Mali (6.03), Sao Tome and Principe (5.70), Libya (5.15), and Egypt (4.24). The largest absolute increases from the model-fitted 2023 reference occurred in Malawi, Ethiopia, the Democratic Republic of the Congo, and Zambia. Projected DALY ASRs were highest in Malawi (189.44 per 100,000), Mali (136.00), Sao Tome and Principe (126.84), Libya (104.82), and Zambia (98.55). Death and DALY trajectories decreased in nine countries each. Declining fitted trajectories for both outcomes occurred in Seychelles, Mauritius, Tunisia, Libya, Egypt, South Africa, Tanzania, and Benin. Cabo Verde additionally had a declining death trajectory, while Eswatini additionally had a declining DALY trajectory.

The direction of fitted change was sensitive to the training window. In the 2010–2019 sensitivity models, the fitted 2030 ASR was higher than the respective end-of-training reference value in 47 countries for incidence, 45 for prevalence, 36 for deaths, and 40 for DALYs. In the 2010–2018 models, the corresponding numbers were 45, 46, 37, and 38. Higher fitted values remained more common than lower values under both sensitivity windows, but the country classifications and projected magnitudes varied with the period used for model fitting.

## Discussion

In this ecological secondary analysis of GBD 2023 modeled estimates, the Africa-wide burden of bladder cancer increased between 2010 and 2023 in both counts and ASRs. In 2023, the highest estimated incidence rates occurred in Tunisia, Malawi, Libya, Egypt, and Mali, while Malawi had the highest mortality and DALY rates. Several countries with comparatively low estimated baseline rates, including Botswana and Gambia, nevertheless had some of the largest positive EAPCs, indicating rapid relative change from a low baseline. Population size was the largest contributor to the increase in counts, although age-structure and age-specific rate changes also contributed. These findings show that the African pattern is heterogeneous rather than a single uniform transition and are broadly consistent with previously reported international variation in bladder cancer burden [2].

Regional variation is compatible with, but cannot be attributed from our aggregate data to, heterogeneous exposure profiles. Smoking and selected occupational carcinogens are established bladder cancer risk factors [16]. A GBD 2019 analysis estimated smoking prevalence among people aged 15 years or older at 18.1% in North Africa and 17.8% in Southern Africa, compared with 10.5%, 8.8%, and 7.0% in Central, Eastern, and Western Africa, respectively [5]; Tunisia was among the countries exceeding 20%. This is compatible with, but does not explain, its high bladder cancer ASR. Infection-related disease also remains relevant in selected endemic settings. In Egypt, schistosomiasis control has coincided historically with declining schistosomiasis-associated squamous carcinoma and a relative shift toward urothelial carcinoma [17]. A hospital series from the Tanzanian Lake Zone reported squamous carcinoma in 57.0% of bladder cancers and *Schistosoma haematobium* eggs in 25.2% of specimens [9], but these findings cannot be extrapolated to all of Eastern Africa. International evidence also links bladder cancer to o-toluidine and selected occupations, but comparable African exposure data are sparse [18]. Our study therefore cannot determine whether smoking, schistosomiasis, or occupational exposures caused the observed country differences.

Lower estimated ASRs should not automatically be interpreted as lower underlying risk. Cancer surveillance varies substantially across Africa. For GLOBOCAN 2020, relatively recent registry data directly informed estimates in only 25 of 48 sub-Saharan African countries, while estimates for 16 countries without such data used information from neighbouring populations [11]. A later appraisal identified 15 sub-Saharan African countries without established cancer registries [19]. Mortality surveillance is similarly uneven: historical studies estimated death-registration completeness at approximately 10% in Nigeria in 2017 but approximately 96% overall in Egypt, with lower completeness in some rural areas [20; 21]. Although these studies did not evaluate the exact GBD 2023 bladder cancer inputs, they demonstrate the heterogeneous data environment. GBD addresses gaps through statistical modeling; estimates from data-sparse settings nevertheless depend more heavily on indirect inputs and assumptions [14]. Thus, low estimates may reflect true differences, incomplete ascertainment, or both.

The decomposition adds interpretation without establishing causation. Population-size change accounted for 43.2% of the increase in incidence, 39.8% in prevalence, 58.8% in deaths, and 50.3% in DALYs. The remaining changes were partitioned between age structure and age-specific rates. The latter should be termed the “age-specific rate-change component,” not “adverse epidemiological change.” For incidence, it may reflect exposure, detection, registration, or modeling changes; for prevalence, it may also reflect survival; and for deaths and DALYs, it may incorporate treatment, survival, and cause-of-death ascertainment. Its negative value in selected countries and outcomes further shows that it is a mathematical component rather than evidence of worsening risk.

DTW clustering likewise provided a descriptive, rather than etiological, summary. The lower-, intermediate-, and higher-burden trajectory groups reflected both ASR level and temporal shape because clustering used unstandardized rates. Because three clusters were prespecified and stability across alternative solutions was not tested, they should not be described as distinct biological phenotypes. They nevertheless illustrate why prevention, surveillance, and care planning should be adapted to country context rather than applied uniformly [22].

The pandemic-era results require particular restraint. GBD-estimated incidence, mortality, and DALY rates during 2020–2023 were above the ARIMA point forecasts, while prevalence changed from below to above its forecast trajectory. These differences are not observed excess cases or proof of a COVID-19 effect. The models used only 10 prepandemic observations, compared estimates primarily with point forecasts, and included no variables describing service disruption. Although a systematic review documented interruptions in cancer screening, diagnosis, and treatment in sub-Saharan Africa, the GBD 2023 cancer study explicitly stated that its estimates did not comprehensively reflect the pandemic’s potential effect because suitable data were unavailable across locations and measures. Our findings therefore represent exploratory pandemic-era deviations, not causal estimates of COVID-19 disruption.

The main log-linear models projected higher fitted ASRs in a majority of countries by 2030, but results varied with the fitting window. These projections are conditional extrapolations, not precise forecasts, and do not incorporate future population change, risk-factor trends, schistosomiasis control, registration, diagnosis, or treatment. Because they predict ASRs rather than absolute counts, they do not directly quantify future service demand. Nevertheless, considered together with population growth, they support forward planning for prevention, diagnostic evaluation, pathology, treatment, and surveillance, with tobacco control and schistosomiasis prevention emphasized according to local exposure patterns [22].

A strength of this study is its consistent comparison of four burden outcomes across 54 countries combined with trend, trajectory, decomposition, pandemic-era, and projection analyses. Several limitations remain. First, GBD estimates depend on heterogeneous source data and modeling, and country UIs cannot eliminate possible systematic error from incomplete ascertainment. Second, downstream analyses used GBD point estimates; draw-level uncertainty was not propagated into EAPC, clustering, decomposition, ARIMA, or projection results. Third, the ecological design and absence of country-year exposure data prevent causal attribution. Fourth, histology, stage, treatment, recurrence, and survival were unavailable. Fifth, clustering used a prespecified three-group solution, and decomposition components do not identify mechanisms. Finally, the short pandemic model and log-linear projections are assumption-dependent; projection intervals reflected coefficient uncertainty without incorporating GBD draw uncertainty or future random variation. These limitations are especially important for countries with sparse surveillance data.

In conclusion, GBD 2023 estimates indicate increasing and geographically heterogeneous bladder cancer burden across Africa. Population growth was the largest driver of increasing counts, while age structure and age-specific rates also contributed. Strengthening locally appropriate prevention, diagnosis, treatment, and cancer-registration systems is essential, but country differences and pandemic-era deviations should not be interpreted causally from these aggregate modeled data.

## Electronic supplementary material

Below is the link to the electronic supplementary material.

Supplementary material 1: Figure 1- Geographic distribution of bladder cancer burden in Africa in 2010. Maps show the age-standardized incidence, prevalence, death, and disability-adjusted life-year (DALY) rates of bladder cancer per 100 000 population across 54 African countries in 2010 for both sexes combined. Countries were grouped into 5 categories for each outcome according to the distribution of country-specific age-standardized rates. DALYs indicate disability-adjusted life-years.

Supplementary material 2: Table 1- Estimated annual percentage change in age-standardized bladder cancer burden across 54 African countries, 2010–2023. This table presents the estimated annual percentage change (EAPC) in age-standardized incidence, prevalence, death, and disability-adjusted life-year (DALY) rates of bladder cancer across 54 African countries from 2010 to 2023 for both sexes combined. For each outcome, the table provides country-specific EAPC estimates with corresponding 95% CIs, together with the 10 countries with the largest increases and the 10 countries with the largest decreases. DALYs indicate disability-adjusted life-years; EAPC, estimated annual percentage change.

Supplementary material 3: Table 2 - Dynamic time warping–based trajectory clustering of bladder cancer incidence across 54 African countries, 2010–2023. This table summarizes the results of dynamic time warping–based clustering of country-level age-standardized incidence rate trajectories for bladder cancer across 54 African countries from 2010 to 2023. Countries are classified into low-stable, moderate-rising, and high-persistent trajectory groups, with corresponding baseline and end-point age-standardized incidence rates, absolute changes, and relative percentage changes. Rates are presented per 100 000 population for both sexes combined.

Supplementary material 4: Table 3 - Das Gupta decomposition of changes in bladder cancer burden across 54 African countries, 2010–2023. This table presents the decomposition of changes in bladder cancer incidence, prevalence, deaths, and disability-adjusted life-years (DALYs) between 2010 and 2023 across 54 African countries. For each outcome, the absolute change in counts is partitioned into contributions from population growth, population aging or age structure change, and epidemiological change in age-specific rates. Summary estimates across outcomes and country-level rankings by overall change and by each decomposition component are also provided. Analyses were based on both sexes combined.

Supplementary material 5: Table 4 - Counterfactual analysis of bladder cancer burden during the COVID-19 period in the African Union, 2020–2023. This table presents the results of counterfactual analyses comparing observed and expected age-standardized incidence, prevalence, death, and disability-adjusted life-year (DALY) rates of bladder cancer in the African Union during 2020 to 2023. Expected values were estimated using autoregressive integrated moving average models fitted to prepandemic trends. The table includes summary estimates for the pandemic period, model diagnostics, and year-specific observed, expected, and gap values for each outcome. Rates are presented per 100 000 population for both sexes combined.

Supplementary material 6: Table 5 - Projected age-standardized bladder cancer burden across Africa to 2030 under the main and sensitivity forecasting windows. This table presents projected age-standardized incidence, prevalence, death, and disability-adjusted life-year (DALY) rates of bladder cancer from 2023 to 2030 under the main forecasting model and 2 sensitivity analyses using alternative training windows. For each outcome, the table provides projected estimates with uncertainty intervals under the main model based on 2010–2023 data and sensitivity models restricted to prepandemic periods. Rates are presented per 100 000 population for both sexes combined.

## Data Availability

All primary data used in this study are publicly available from the Global Burden of Disease (GBD) 2023 study through the IHME GBD Results Tool (https://vizhub.healthdata.org/gbd-results/). The analytic code and derived outputs generated during the current study are available from the corresponding author upon reasonable request.
