# Supplementary material for: Bladder cancer in Africa, 2010–2023: burden, geographic heterogeneity, demographic drivers, pandemic-era temporal changes, and projections to 2030
Source: Infect Agent Cancer. 2026 Jul 23;21:69. doi: 10.1186/s13027-026-00782-3 (PMC13681883; doi:10.1186/s13027-026-00782-3)

Bladder cancer burden in Africa (2010)

Age−standardized rates per 100,000 population (Both sexes)

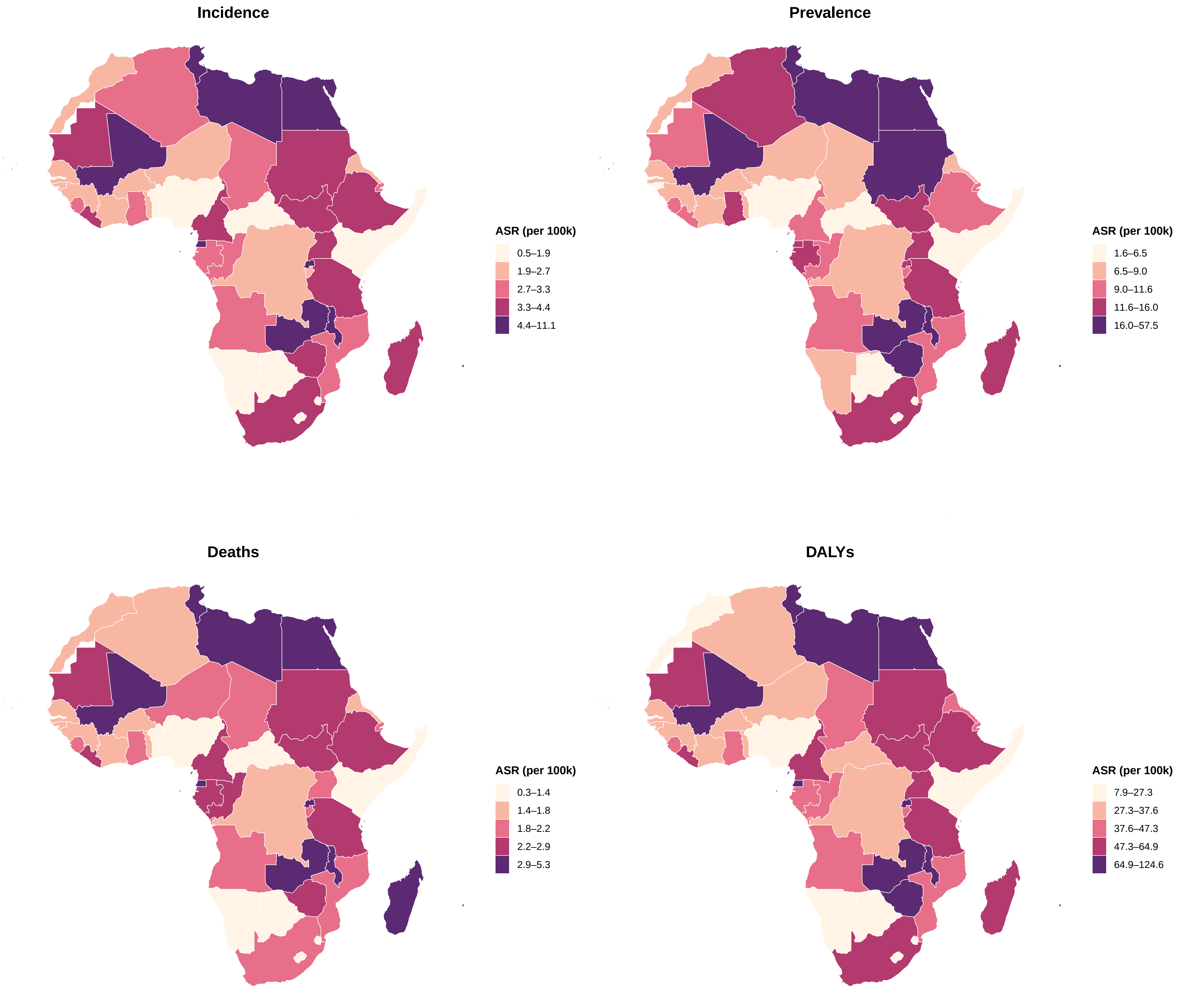

Supplement: Supplementary file 1 [file 13027_2026_782_MOESM1_ESM.pdf]
