# Supplementary material for: Bladder cancer in Africa, 2010–2023: burden, geographic heterogeneity, demographic drivers, pandemic-era temporal changes, and projections to 2030
Source: Infect Agent Cancer. 2026 Jul 23;21:69. doi: 10.1186/s13027-026-00782-3 (PMC13681883; doi:10.1186/s13027-026-00782-3)
Supplement: Supplementary file 2 [file 13027_2026_782_MOESM2_ESM.docx]

EAPC_all

| measure | location | EAPC | LCI | UCI | EAPC_95CI |
| --- | --- | --- | --- | --- | --- |
| DALYs (Disability-Adjusted Life Years) | Gambia | 3.251225262 | 2.499675247 | 4.008285807 | 3.25 (2.50, 4.01) |
| DALYs (Disability-Adjusted Life Years) | Botswana | 2.960839696 | 2.530548173 | 3.392937031 | 2.96 (2.53, 3.39) |
| DALYs (Disability-Adjusted Life Years) | Democratic Republic of the Congo | 2.772691499 | 2.263967736 | 3.283945966 | 2.77 (2.26, 3.28) |
| DALYs (Disability-Adjusted Life Years) | Ethiopia | 2.767170314 | 2.29513725 | 3.24138154 | 2.77 (2.30, 3.24) |
| DALYs (Disability-Adjusted Life Years) | Côte d'Ivoire | 2.567921176 | 1.90644633 | 3.233689656 | 2.57 (1.91, 3.23) |
| DALYs (Disability-Adjusted Life Years) | Ghana | 2.323985295 | 1.953689306 | 2.6956262 | 2.32 (1.95, 2.70) |
| DALYs (Disability-Adjusted Life Years) | Malawi | 2.252434316 | 1.065401472 | 3.453409092 | 2.25 (1.07, 3.45) |
| DALYs (Disability-Adjusted Life Years) | Congo | 2.052823316 | 1.595180119 | 2.512528001 | 2.05 (1.60, 2.51) |
| DALYs (Disability-Adjusted Life Years) | Morocco | 2.034881588 | 1.779154611 | 2.291251096 | 2.03 (1.78, 2.29) |
| DALYs (Disability-Adjusted Life Years) | Eritrea | 2.029958061 | 1.489934741 | 2.572854819 | 2.03 (1.49, 2.57) |
| DALYs (Disability-Adjusted Life Years) | Gabon | 1.864281459 | 1.34436339 | 2.386866817 | 1.86 (1.34, 2.39) |
| DALYs (Disability-Adjusted Life Years) | Somalia | 1.849482322 | 1.042962039 | 2.662440213 | 1.85 (1.04, 2.66) |
| DALYs (Disability-Adjusted Life Years) | Sierra Leone | 1.760171879 | 1.416506538 | 2.105001783 | 1.76 (1.42, 2.11) |
| DALYs (Disability-Adjusted Life Years) | Zambia | 1.671594156 | 1.094966672 | 2.251510619 | 1.67 (1.09, 2.25) |
| DALYs (Disability-Adjusted Life Years) | Togo | 1.660577086 | 0.975363076 | 2.350440926 | 1.66 (0.98, 2.35) |
| DALYs (Disability-Adjusted Life Years) | Guinea-Bissau | 1.658083806 | 1.216407777 | 2.101687168 | 1.66 (1.22, 2.10) |
| DALYs (Disability-Adjusted Life Years) | Rwanda | 1.473935936 | 0.831744167 | 2.120217788 | 1.47 (0.83, 2.12) |
| DALYs (Disability-Adjusted Life Years) | Equatorial Guinea | 1.459598524 | 0.784627687 | 2.139089749 | 1.46 (0.78, 2.14) |
| DALYs (Disability-Adjusted Life Years) | Lesotho | 1.4325544 | 0.527653105 | 2.345601179 | 1.43 (0.53, 2.35) |
| DALYs (Disability-Adjusted Life Years) | Burundi | 1.427046164 | 1.006604901 | 1.849237518 | 1.43 (1.01, 1.85) |
| DALYs (Disability-Adjusted Life Years) | Djibouti | 1.407166417 | 1.117430968 | 1.697732057 | 1.41 (1.12, 1.70) |
| DALYs (Disability-Adjusted Life Years) | Mali | 1.336768401 | 0.772384508 | 1.904313172 | 1.34 (0.77, 1.90) |
| DALYs (Disability-Adjusted Life Years) | Sao Tome and Principe | 1.285169494 | 0.864598429 | 1.707494196 | 1.29 (0.86, 1.71) |
| DALYs (Disability-Adjusted Life Years) | Madagascar | 1.275386427 | 0.57312116 | 1.982555355 | 1.28 (0.57, 1.98) |
| DALYs (Disability-Adjusted Life Years) | Kenya | 1.233656568 | 0.431542717 | 2.042176639 | 1.23 (0.43, 2.04) |
| DALYs (Disability-Adjusted Life Years) | Guinea | 1.221059583 | 0.669666929 | 1.77547235 | 1.22 (0.67, 1.78) |
| DALYs (Disability-Adjusted Life Years) | Central African Republic | 1.185998571 | 0.555293678 | 1.820659383 | 1.19 (0.56, 1.82) |
| DALYs (Disability-Adjusted Life Years) | Algeria | 1.167306447 | 0.936549062 | 1.39859138 | 1.17 (0.94, 1.40) |
| DALYs (Disability-Adjusted Life Years) | Mauritania | 1.109670193 | 0.2796546 | 1.946555834 | 1.11 (0.28, 1.95) |
| DALYs (Disability-Adjusted Life Years) | Comoros | 1.071199674 | 0.253962706 | 1.895098486 | 1.07 (0.25, 1.90) |
| DALYs (Disability-Adjusted Life Years) | Liberia | 1.060931939 | 0.371656679 | 1.754940612 | 1.06 (0.37, 1.75) |
| DALYs (Disability-Adjusted Life Years) | Senegal | 1.010512933 | 0.216673262 | 1.810640793 | 1.01 (0.22, 1.81) |
| DALYs (Disability-Adjusted Life Years) | Cabo Verde | 1.007386161 | 0.714213913 | 1.301411813 | 1.01 (0.71, 1.30) |
| DALYs (Disability-Adjusted Life Years) | Namibia | 0.961686732 | 0.392098542 | 1.534506557 | 0.96 (0.39, 1.53) |
| DALYs (Disability-Adjusted Life Years) | Niger | 0.955613729 | 0.657416363 | 1.254694503 | 0.96 (0.66, 1.25) |
| DALYs (Disability-Adjusted Life Years) | Cameroon | 0.861878994 | 0.542561053 | 1.182211072 | 0.86 (0.54, 1.18) |
| DALYs (Disability-Adjusted Life Years) | Chad | 0.851645914 | 0.334788995 | 1.37116533 | 0.85 (0.33, 1.37) |
| DALYs (Disability-Adjusted Life Years) | Uganda | 0.668498864 | 0.072766172 | 1.267777949 | 0.67 (0.07, 1.27) |
| DALYs (Disability-Adjusted Life Years) | Sudan | 0.655528432 | 0.327254658 | 0.984876328 | 0.66 (0.33, 0.98) |
| DALYs (Disability-Adjusted Life Years) | Burkina Faso | 0.647226603 | -0.077169359 | 1.376874113 | 0.65 (-0.08, 1.38) |
| DALYs (Disability-Adjusted Life Years) | Nigeria | 0.605598572 | 0.244639333 | 0.967857547 | 0.61 (0.24, 0.97) |
| DALYs (Disability-Adjusted Life Years) | South Sudan | 0.547519382 | 0.035588419 | 1.062070145 | 0.55 (0.04, 1.06) |
| DALYs (Disability-Adjusted Life Years) | Mozambique | 0.432486951 | -0.175091992 | 1.043763891 | 0.43 (-0.18, 1.04) |
| DALYs (Disability-Adjusted Life Years) | Angola | 0.184920793 | -0.292763212 | 0.664893318 | 0.18 (-0.29, 0.66) |
| DALYs (Disability-Adjusted Life Years) | Zimbabwe | 0.122172207 | -0.653394036 | 0.903793042 | 0.12 (-0.65, 0.90) |
| DALYs (Disability-Adjusted Life Years) | Benin | -0.063460194 | -0.311978145 | 0.185677301 | -0.06 (-0.31, 0.19) |
| DALYs (Disability-Adjusted Life Years) | Tunisia | -0.308600697 | -1.0718798 | 0.460567481 | -0.31 (-1.07, 0.46) |
| DALYs (Disability-Adjusted Life Years) | United Republic of Tanzania | -0.478333119 | -1.024652752 | 0.071002065 | -0.48 (-1.02, 0.07) |
| DALYs (Disability-Adjusted Life Years) | Eswatini | -0.47989157 | -1.815244318 | 0.873622521 | -0.48 (-1.82, 0.87) |
| DALYs (Disability-Adjusted Life Years) | Egypt | -0.533010907 | -1.464553852 | 0.407338739 | -0.53 (-1.46, 0.41) |
| DALYs (Disability-Adjusted Life Years) | Libya | -0.54413182 | -1.057231645 | -0.028371149 | -0.54 (-1.06, -0.03) |
| DALYs (Disability-Adjusted Life Years) | South Africa | -0.689801362 | -1.514100374 | 0.141396798 | -0.69 (-1.51, 0.14) |
| DALYs (Disability-Adjusted Life Years) | Mauritius | -1.251898984 | -1.724356647 | -0.777169993 | -1.25 (-1.72, -0.78) |
| DALYs (Disability-Adjusted Life Years) | Seychelles | -1.5055312 | -2.192794 | -0.813439204 | -1.51 (-2.19, -0.81) |
| Deaths | Gambia | 3.197885394 | 2.440791331 | 3.960574801 | 3.20 (2.44, 3.96) |
| Deaths | Botswana | 2.863408364 | 2.379978454 | 3.349120991 | 2.86 (2.38, 3.35) |
| Deaths | Democratic Republic of the Congo | 2.649637092 | 2.056162951 | 3.246562387 | 2.65 (2.06, 3.25) |
| Deaths | Ethiopia | 2.559121676 | 2.122641934 | 2.997466965 | 2.56 (2.12, 3.00) |
| Deaths | Ghana | 2.211837945 | 1.89765284 | 2.526991789 | 2.21 (1.90, 2.53) |
| Deaths | Malawi | 2.101885444 | 0.758912204 | 3.462758611 | 2.10 (0.76, 3.46) |
| Deaths | Congo | 2.07419379 | 1.647153474 | 2.503028189 | 2.07 (1.65, 2.50) |
| Deaths | Côte d'Ivoire | 2.046985484 | 1.403429626 | 2.694625663 | 2.05 (1.40, 2.69) |
| Deaths | Eritrea | 1.942336476 | 1.335313712 | 2.552995451 | 1.94 (1.34, 2.55) |
| Deaths | Gabon | 1.912431489 | 1.43609255 | 2.391007292 | 1.91 (1.44, 2.39) |
| Deaths | Somalia | 1.836610381 | 0.874968147 | 2.807419962 | 1.84 (0.87, 2.81) |
| Deaths | Morocco | 1.818930137 | 1.540050165 | 2.098576053 | 1.82 (1.54, 2.10) |
| Deaths | Zambia | 1.751850844 | 1.189377555 | 2.317450707 | 1.75 (1.19, 2.32) |
| Deaths | Lesotho | 1.599082673 | 0.790350824 | 2.414303707 | 1.60 (0.79, 2.41) |
| Deaths | Sierra Leone | 1.590038731 | 1.232078934 | 1.949264285 | 1.59 (1.23, 1.95) |
| Deaths | Guinea-Bissau | 1.447001446 | 0.9951225 | 1.900902218 | 1.45 (1.00, 1.90) |
| Deaths | Togo | 1.380344823 | 0.74991412 | 2.014720372 | 1.38 (0.75, 2.01) |
| Deaths | Comoros | 1.336354342 | 0.425958362 | 2.255003376 | 1.34 (0.43, 2.26) |
| Deaths | Equatorial Guinea | 1.29318118 | 0.76509211 | 1.824037855 | 1.29 (0.77, 1.82) |
| Deaths | Guinea | 1.283569457 | 0.728951274 | 1.841241393 | 1.28 (0.73, 1.84) |
| Deaths | Kenya | 1.239984075 | 0.348291693 | 2.139600012 | 1.24 (0.35, 2.14) |
| Deaths | Madagascar | 1.239546151 | 0.601892532 | 1.881241465 | 1.24 (0.60, 1.88) |
| Deaths | Burundi | 1.150299494 | 0.69960278 | 1.603013372 | 1.15 (0.70, 1.60) |
| Deaths | Mali | 1.138748844 | 0.603706538 | 1.676636674 | 1.14 (0.60, 1.68) |
| Deaths | Djibouti | 1.129904947 | 0.837409023 | 1.423249305 | 1.13 (0.84, 1.42) |
| Deaths | Rwanda | 1.117984897 | 0.551972402 | 1.687183507 | 1.12 (0.55, 1.69) |
| Deaths | Mauritania | 1.111306885 | 0.29550434 | 1.933745159 | 1.11 (0.30, 1.93) |
| Deaths | Central African Republic | 1.095770958 | 0.47942279 | 1.71589985 | 1.10 (0.48, 1.72) |
| Deaths | Sao Tome and Principe | 1.056735174 | 0.535039098 | 1.581138433 | 1.06 (0.54, 1.58) |
| Deaths | Algeria | 1.016850664 | 0.801465665 | 1.232695882 | 1.02 (0.80, 1.23) |
| Deaths | Senegal | 0.94484059 | 0.234839015 | 1.659871377 | 0.94 (0.23, 1.66) |
| Deaths | Namibia | 0.925167108 | 0.368265537 | 1.485158693 | 0.93 (0.37, 1.49) |
| Deaths | Chad | 0.875434695 | 0.382034051 | 1.371260517 | 0.88 (0.38, 1.37) |
| Deaths | Niger | 0.841766151 | 0.556188557 | 1.128154781 | 0.84 (0.56, 1.13) |
| Deaths | Nigeria | 0.775771611 | 0.479649478 | 1.072766441 | 0.78 (0.48, 1.07) |
| Deaths | Burkina Faso | 0.73774729 | 0.085819256 | 1.393921781 | 0.74 (0.09, 1.39) |
| Deaths | Cameroon | 0.698048987 | 0.40060494 | 0.996374233 | 0.70 (0.40, 1.00) |
| Deaths | Mozambique | 0.679305283 | 0.132792336 | 1.228801033 | 0.68 (0.13, 1.23) |
| Deaths | Liberia | 0.671430448 | -0.022473208 | 1.370150209 | 0.67 (-0.02, 1.37) |
| Deaths | Uganda | 0.658284805 | 0.048823735 | 1.271458491 | 0.66 (0.05, 1.27) |
| Deaths | Sudan | 0.598321132 | 0.252001207 | 0.945837417 | 0.60 (0.25, 0.95) |
| Deaths | Zimbabwe | 0.482192622 | -0.372925856 | 1.344650748 | 0.48 (-0.37, 1.34) |
| Deaths | South Sudan | 0.47323634 | -0.010804379 | 0.959620266 | 0.47 (-0.01, 0.96) |
| Deaths | Eswatini | 0.211362687 | -1.000863929 | 1.438432799 | 0.21 (-1.00, 1.44) |
| Deaths | Angola | 0.103294082 | -0.321702425 | 0.530102639 | 0.10 (-0.32, 0.53) |
| Deaths | Cabo Verde | 0.001984277 | -0.408606595 | 0.414267915 | 0.00 (-0.41, 0.41) |
| Deaths | United Republic of Tanzania | -0.037608999 | -0.607321135 | 0.535368688 | -0.04 (-0.61, 0.54) |
| Deaths | Egypt | -0.085430595 | -1.009205537 | 0.846964947 | -0.09 (-1.01, 0.85) |
| Deaths | Benin | -0.12846735 | -0.382108688 | 0.125819794 | -0.13 (-0.38, 0.13) |
| Deaths | Libya | -0.190516427 | -0.708588736 | 0.330259026 | -0.19 (-0.71, 0.33) |
| Deaths | South Africa | -0.56851268 | -1.425208929 | 0.295628966 | -0.57 (-1.43, 0.30) |
| Deaths | Tunisia | -0.695933493 | -1.327359494 | -0.06046687 | -0.70 (-1.33, -0.06) |
| Deaths | Mauritius | -1.268531661 | -1.792091021 | -0.742181137 | -1.27 (-1.79, -0.74) |
| Deaths | Seychelles | -1.466358622 | -2.176365435 | -0.751198559 | -1.47 (-2.18, -0.75) |
| Incidence | Botswana | 3.910075333 | 3.448419705 | 4.373791175 | 3.91 (3.45, 4.37) |
| Incidence | Gambia | 3.414784043 | 2.668188436 | 4.166808839 | 3.41 (2.67, 4.17) |
| Incidence | Ethiopia | 3.401505223 | 2.916731408 | 3.888562493 | 3.40 (2.92, 3.89) |
| Incidence | Democratic Republic of the Congo | 3.264321482 | 2.66935699 | 3.862733767 | 3.26 (2.67, 3.86) |
| Incidence | Côte d'Ivoire | 2.888782241 | 2.270708764 | 3.510591048 | 2.89 (2.27, 3.51) |
| Incidence | Ghana | 2.868548103 | 2.397702788 | 3.341558461 | 2.87 (2.40, 3.34) |
| Incidence | Morocco | 2.796717082 | 2.484806144 | 3.109577316 | 2.80 (2.48, 3.11) |
| Incidence | Malawi | 2.661860684 | 1.40508266 | 3.934214762 | 2.66 (1.41, 3.93) |
| Incidence | Congo | 2.540797904 | 2.063500155 | 3.020327726 | 2.54 (2.06, 3.02) |
| Incidence | Gabon | 2.515108715 | 1.971654462 | 3.061459288 | 2.52 (1.97, 3.06) |
| Incidence | Eritrea | 2.425754565 | 1.859988583 | 2.994663008 | 2.43 (1.86, 2.99) |
| Incidence | Zambia | 2.350516031 | 1.786883608 | 2.9172695 | 2.35 (1.79, 2.92) |
| Incidence | Sierra Leone | 2.193478209 | 1.842983424 | 2.545179229 | 2.19 (1.84, 2.55) |
| Incidence | Algeria | 2.087011356 | 1.868957387 | 2.305532077 | 2.09 (1.87, 2.31) |
| Incidence | Togo | 2.06875426 | 1.351661135 | 2.790921033 | 2.07 (1.35, 2.79) |
| Incidence | Somalia | 1.99230089 | 1.140720176 | 2.85105171 | 1.99 (1.14, 2.85) |
| Incidence | Equatorial Guinea | 1.936584561 | 1.258025005 | 2.619691343 | 1.94 (1.26, 2.62) |
| Incidence | Sao Tome and Principe | 1.936092578 | 1.481642564 | 2.392577688 | 1.94 (1.48, 2.39) |
| Incidence | Guinea-Bissau | 1.911320742 | 1.441740548 | 2.383074653 | 1.91 (1.44, 2.38) |
| Incidence | Djibouti | 1.879649832 | 1.574299832 | 2.185917768 | 1.88 (1.57, 2.19) |
| Incidence | Rwanda | 1.878218005 | 1.215028766 | 2.545752645 | 1.88 (1.22, 2.55) |
| Incidence | Kenya | 1.844457029 | 1.019847011 | 2.675798216 | 1.84 (1.02, 2.68) |
| Incidence | Lesotho | 1.828848517 | 0.981390616 | 2.68341847 | 1.83 (0.98, 2.68) |
| Incidence | Mauritania | 1.708848784 | 0.894553091 | 2.529716461 | 1.71 (0.89, 2.53) |
| Incidence | Madagascar | 1.658578829 | 0.892557442 | 2.430416192 | 1.66 (0.89, 2.43) |
| Incidence | Guinea | 1.60659547 | 1.013232353 | 2.20344407 | 1.61 (1.01, 2.20) |
| Incidence | Burundi | 1.601908921 | 1.17402706 | 2.031600366 | 1.60 (1.17, 2.03) |
| Incidence | Namibia | 1.579949586 | 1.016258819 | 2.14678586 | 1.58 (1.02, 2.15) |
| Incidence | Mali | 1.576441195 | 0.973502501 | 2.182980191 | 1.58 (0.97, 2.18) |
| Incidence | Sudan | 1.535606361 | 1.153527579 | 1.919128336 | 1.54 (1.15, 1.92) |
| Incidence | Senegal | 1.410311071 | 0.575067681 | 2.252490887 | 1.41 (0.58, 2.25) |
| Incidence | Cameroon | 1.396384141 | 1.050522917 | 1.74342913 | 1.40 (1.05, 1.74) |
| Incidence | Central African Republic | 1.387055372 | 0.707269267 | 2.071430114 | 1.39 (0.71, 2.07) |
| Incidence | Cabo Verde | 1.374814738 | 0.969323861 | 1.781934059 | 1.37 (0.97, 1.78) |
| Incidence | Liberia | 1.346220356 | 0.640418663 | 2.056971909 | 1.35 (0.64, 2.06) |
| Incidence | Comoros | 1.277147102 | 0.399108358 | 2.162864719 | 1.28 (0.40, 2.16) |
| Incidence | Uganda | 1.201404615 | 0.51753813 | 1.889923755 | 1.20 (0.52, 1.89) |
| Incidence | Nigeria | 1.171554832 | 0.804329224 | 1.540118227 | 1.17 (0.80, 1.54) |
| Incidence | Chad | 1.069734801 | 0.556596064 | 1.585492077 | 1.07 (0.56, 1.59) |
| Incidence | Niger | 1.026286906 | 0.681045101 | 1.372712568 | 1.03 (0.68, 1.37) |
| Incidence | Mozambique | 0.953941345 | 0.278062951 | 1.634375188 | 0.95 (0.28, 1.63) |
| Incidence | Burkina Faso | 0.809722341 | 0.042921938 | 1.582400049 | 0.81 (0.04, 1.58) |
| Incidence | Angola | 0.665565407 | 0.195499071 | 1.137837056 | 0.67 (0.20, 1.14) |
| Incidence | Tunisia | 0.561412754 | -0.13170148 | 1.259337397 | 0.56 (-0.13, 1.26) |
| Incidence | Zimbabwe | 0.544094939 | -0.255202727 | 1.34979772 | 0.54 (-0.26, 1.35) |
| Incidence | Egypt | 0.526295776 | -0.456287927 | 1.518578441 | 0.53 (-0.46, 1.52) |
| Incidence | South Sudan | 0.464848521 | -0.064842456 | 0.997347044 | 0.46 (-0.06, 1.00) |
| Incidence | Benin | 0.399502392 | 0.133066277 | 0.666647447 | 0.40 (0.13, 0.67) |
| Incidence | Eswatini | 0.301987734 | -0.999426435 | 1.62050967 | 0.30 (-1.00, 1.62) |
| Incidence | United Republic of Tanzania | 0.093267915 | -0.467666956 | 0.65736405 | 0.09 (-0.47, 0.66) |
| Incidence | South Africa | -0.206099293 | -1.08882027 | 0.684499421 | -0.21 (-1.09, 0.68) |
| Incidence | Libya | -0.518228611 | -1.135989988 | 0.103392908 | -0.52 (-1.14, 0.10) |
| Incidence | Seychelles | -0.885324181 | -1.725325818 | -0.038142639 | -0.89 (-1.73, -0.04) |
| Incidence | Mauritius | -0.929441341 | -1.434592154 | -0.421701614 | -0.93 (-1.43, -0.42) |
| Prevalence | Botswana | 5.021608063 | 4.764612501 | 5.279234055 | 5.02 (4.76, 5.28) |
| Prevalence | Democratic Republic of the Congo | 3.749726935 | 3.24093181 | 4.26102952 | 3.75 (3.24, 4.26) |
| Prevalence | Ethiopia | 3.699983105 | 3.096485792 | 4.307013118 | 3.70 (3.10, 4.31) |
| Prevalence | Côte d'Ivoire | 3.655771954 | 3.152715827 | 4.161281389 | 3.66 (3.15, 4.16) |
| Prevalence | Gambia | 3.087640354 | 2.441565176 | 3.737790178 | 3.09 (2.44, 3.74) |
| Prevalence | Ghana | 3.001093848 | 2.395906692 | 3.609857821 | 3.00 (2.40, 3.61) |
| Prevalence | Eritrea | 2.854222365 | 2.467801769 | 3.242100207 | 2.85 (2.47, 3.24) |
| Prevalence | Gabon | 2.81527934 | 2.197980147 | 3.436307162 | 2.82 (2.20, 3.44) |
| Prevalence | Morocco | 2.758183656 | 2.321435729 | 3.196795794 | 2.76 (2.32, 3.20) |
| Prevalence | Djibouti | 2.566382211 | 2.275873898 | 2.857715694 | 2.57 (2.28, 2.86) |
| Prevalence | Tunisia | 2.531319855 | 0.743211373 | 4.351165781 | 2.53 (0.74, 4.35) |
| Prevalence | Togo | 2.452404669 | 1.730247393 | 3.179688358 | 2.45 (1.73, 3.18) |
| Prevalence | Sierra Leone | 2.449606727 | 2.074982668 | 2.825605688 | 2.45 (2.07, 2.83) |
| Prevalence | Guinea-Bissau | 2.447224021 | 1.972817909 | 2.923837204 | 2.45 (1.97, 2.92) |
| Prevalence | Malawi | 2.427732035 | 2.033168563 | 2.823821289 | 2.43 (2.03, 2.82) |
| Prevalence | Rwanda | 2.413373274 | 1.69627434 | 3.135528745 | 2.41 (1.70, 3.14) |
| Prevalence | Congo | 2.397247168 | 1.936338102 | 2.860240251 | 2.40 (1.94, 2.86) |
| Prevalence | Algeria | 2.392432266 | 1.986954601 | 2.799522022 | 2.39 (1.99, 2.80) |
| Prevalence | Sao Tome and Principe | 2.363395699 | 2.133832888 | 2.593474491 | 2.36 (2.13, 2.59) |
| Prevalence | Zambia | 2.292418755 | 2.028506687 | 2.557013472 | 2.29 (2.03, 2.56) |
| Prevalence | Kenya | 2.256964897 | 1.67285496 | 2.844430541 | 2.26 (1.67, 2.84) |
| Prevalence | Sudan | 2.240325234 | 1.857812305 | 2.624274637 | 2.24 (1.86, 2.62) |
| Prevalence | Cameroon | 2.150968119 | 1.584060672 | 2.721039291 | 2.15 (1.58, 2.72) |
| Prevalence | Somalia | 2.088528535 | 1.564798065 | 2.614959681 | 2.09 (1.56, 2.61) |
| Prevalence | Mauritania | 1.982486354 | 1.197348295 | 2.773715895 | 1.98 (1.20, 2.77) |
| Prevalence | Burundi | 1.900742262 | 1.654708472 | 2.147371525 | 1.90 (1.65, 2.15) |
| Prevalence | Madagascar | 1.898426355 | 1.030019636 | 2.774297492 | 1.90 (1.03, 2.77) |
| Prevalence | Central African Republic | 1.872146798 | 1.114733246 | 2.635233859 | 1.87 (1.11, 2.64) |
| Prevalence | Guinea | 1.869272509 | 1.254544089 | 2.487733018 | 1.87 (1.25, 2.49) |
| Prevalence | Mali | 1.853796681 | 1.217372794 | 2.494222206 | 1.85 (1.22, 2.49) |
| Prevalence | Uganda | 1.775831302 | 1.158315083 | 2.397117121 | 1.78 (1.16, 2.40) |
| Prevalence | Mozambique | 1.693067438 | 1.32452865 | 2.06294668 | 1.69 (1.32, 2.06) |
| Prevalence | Senegal | 1.5445977 | 0.594934247 | 2.503226423 | 1.54 (0.59, 2.50) |
| Prevalence | Equatorial Guinea | 1.542620878 | 0.919153267 | 2.169940205 | 1.54 (0.92, 2.17) |
| Prevalence | Nigeria | 1.388473101 | 0.900922593 | 1.87837944 | 1.39 (0.90, 1.88) |
| Prevalence | Liberia | 1.374224715 | 0.649817594 | 2.103845613 | 1.37 (0.65, 2.10) |
| Prevalence | Namibia | 1.359709433 | 1.191920762 | 1.527776319 | 1.36 (1.19, 1.53) |
| Prevalence | Angola | 1.297210663 | 0.777490482 | 1.819611096 | 1.30 (0.78, 1.82) |
| Prevalence | Niger | 1.058848901 | 0.595976975 | 1.523850638 | 1.06 (0.60, 1.52) |
| Prevalence | Benin | 1.035726376 | 0.704085157 | 1.368459764 | 1.04 (0.70, 1.37) |
| Prevalence | Chad | 1.016481787 | 0.51542567 | 1.520035603 | 1.02 (0.52, 1.52) |
| Prevalence | Comoros | 1.000124038 | 0.256162154 | 1.749606573 | 1.00 (0.26, 1.75) |
| Prevalence | Lesotho | 0.790869536 | 0.071141702 | 1.515773769 | 0.79 (0.07, 1.52) |
| Prevalence | Burkina Faso | 0.741505912 | -0.050571042 | 1.539859899 | 0.74 (-0.05, 1.54) |
| Prevalence | Cabo Verde | 0.495157423 | 0.038729775 | 0.953667527 | 0.50 (0.04, 0.95) |
| Prevalence | Zimbabwe | 0.206091647 | 0.026484708 | 0.386021087 | 0.21 (0.03, 0.39) |
| Prevalence | United Republic of Tanzania | 0.19508081 | -0.127887765 | 0.519093808 | 0.20 (-0.13, 0.52) |
| Prevalence | Egypt | 0.192923936 | -0.616018172 | 1.008450479 | 0.19 (-0.62, 1.01) |
| Prevalence | Eswatini | 0.143423764 | -1.045907958 | 1.347050093 | 0.14 (-1.05, 1.35) |
| Prevalence | South Africa | 0.10755408 | -1.450469714 | 1.690209531 | 0.11 (-1.45, 1.69) |
| Prevalence | South Sudan | 0.033105492 | -0.535263697 | 0.604722502 | 0.03 (-0.54, 0.60) |
| Prevalence | Seychelles | -0.114176017 | -0.917048776 | 0.695202449 | -0.11 (-0.92, 0.70) |
| Prevalence | Mauritius | -0.598900955 | -1.071675403 | -0.123867136 | -0.60 (-1.07, -0.12) |
| Prevalence | Libya | -1.422712111 | -2.03433503 | -0.807270685 | -1.42 (-2.03, -0.81) |

Top10_by_measure

| measure | location | EAPC | LCI | UCI | EAPC_95CI |
| --- | --- | --- | --- | --- | --- |
| DALYs (Disability-Adjusted Life Years) | Gambia | 3.251225262 | 2.499675247 | 4.008285807 | 3.25 (2.50, 4.01) |
| DALYs (Disability-Adjusted Life Years) | Botswana | 2.960839696 | 2.530548173 | 3.392937031 | 2.96 (2.53, 3.39) |
| DALYs (Disability-Adjusted Life Years) | Democratic Republic of the Congo | 2.772691499 | 2.263967736 | 3.283945966 | 2.77 (2.26, 3.28) |
| DALYs (Disability-Adjusted Life Years) | Ethiopia | 2.767170314 | 2.29513725 | 3.24138154 | 2.77 (2.30, 3.24) |
| DALYs (Disability-Adjusted Life Years) | Côte d'Ivoire | 2.567921176 | 1.90644633 | 3.233689656 | 2.57 (1.91, 3.23) |
| DALYs (Disability-Adjusted Life Years) | Ghana | 2.323985295 | 1.953689306 | 2.6956262 | 2.32 (1.95, 2.70) |
| DALYs (Disability-Adjusted Life Years) | Malawi | 2.252434316 | 1.065401472 | 3.453409092 | 2.25 (1.07, 3.45) |
| DALYs (Disability-Adjusted Life Years) | Congo | 2.052823316 | 1.595180119 | 2.512528001 | 2.05 (1.60, 2.51) |
| DALYs (Disability-Adjusted Life Years) | Morocco | 2.034881588 | 1.779154611 | 2.291251096 | 2.03 (1.78, 2.29) |
| DALYs (Disability-Adjusted Life Years) | Eritrea | 2.029958061 | 1.489934741 | 2.572854819 | 2.03 (1.49, 2.57) |
| Deaths | Gambia | 3.197885394 | 2.440791331 | 3.960574801 | 3.20 (2.44, 3.96) |
| Deaths | Botswana | 2.863408364 | 2.379978454 | 3.349120991 | 2.86 (2.38, 3.35) |
| Deaths | Democratic Republic of the Congo | 2.649637092 | 2.056162951 | 3.246562387 | 2.65 (2.06, 3.25) |
| Deaths | Ethiopia | 2.559121676 | 2.122641934 | 2.997466965 | 2.56 (2.12, 3.00) |
| Deaths | Ghana | 2.211837945 | 1.89765284 | 2.526991789 | 2.21 (1.90, 2.53) |
| Deaths | Malawi | 2.101885444 | 0.758912204 | 3.462758611 | 2.10 (0.76, 3.46) |
| Deaths | Congo | 2.07419379 | 1.647153474 | 2.503028189 | 2.07 (1.65, 2.50) |
| Deaths | Côte d'Ivoire | 2.046985484 | 1.403429626 | 2.694625663 | 2.05 (1.40, 2.69) |
| Deaths | Eritrea | 1.942336476 | 1.335313712 | 2.552995451 | 1.94 (1.34, 2.55) |
| Deaths | Gabon | 1.912431489 | 1.43609255 | 2.391007292 | 1.91 (1.44, 2.39) |
| Incidence | Botswana | 3.910075333 | 3.448419705 | 4.373791175 | 3.91 (3.45, 4.37) |
| Incidence | Gambia | 3.414784043 | 2.668188436 | 4.166808839 | 3.41 (2.67, 4.17) |
| Incidence | Ethiopia | 3.401505223 | 2.916731408 | 3.888562493 | 3.40 (2.92, 3.89) |
| Incidence | Democratic Republic of the Congo | 3.264321482 | 2.66935699 | 3.862733767 | 3.26 (2.67, 3.86) |
| Incidence | Côte d'Ivoire | 2.888782241 | 2.270708764 | 3.510591048 | 2.89 (2.27, 3.51) |
| Incidence | Ghana | 2.868548103 | 2.397702788 | 3.341558461 | 2.87 (2.40, 3.34) |
| Incidence | Morocco | 2.796717082 | 2.484806144 | 3.109577316 | 2.80 (2.48, 3.11) |
| Incidence | Malawi | 2.661860684 | 1.40508266 | 3.934214762 | 2.66 (1.41, 3.93) |
| Incidence | Congo | 2.540797904 | 2.063500155 | 3.020327726 | 2.54 (2.06, 3.02) |
| Incidence | Gabon | 2.515108715 | 1.971654462 | 3.061459288 | 2.52 (1.97, 3.06) |
| Prevalence | Botswana | 5.021608063 | 4.764612501 | 5.279234055 | 5.02 (4.76, 5.28) |
| Prevalence | Democratic Republic of the Congo | 3.749726935 | 3.24093181 | 4.26102952 | 3.75 (3.24, 4.26) |
| Prevalence | Ethiopia | 3.699983105 | 3.096485792 | 4.307013118 | 3.70 (3.10, 4.31) |
| Prevalence | Côte d'Ivoire | 3.655771954 | 3.152715827 | 4.161281389 | 3.66 (3.15, 4.16) |
| Prevalence | Gambia | 3.087640354 | 2.441565176 | 3.737790178 | 3.09 (2.44, 3.74) |
| Prevalence | Ghana | 3.001093848 | 2.395906692 | 3.609857821 | 3.00 (2.40, 3.61) |
| Prevalence | Eritrea | 2.854222365 | 2.467801769 | 3.242100207 | 2.85 (2.47, 3.24) |
| Prevalence | Gabon | 2.81527934 | 2.197980147 | 3.436307162 | 2.82 (2.20, 3.44) |
| Prevalence | Morocco | 2.758183656 | 2.321435729 | 3.196795794 | 2.76 (2.32, 3.20) |
| Prevalence | Djibouti | 2.566382211 | 2.275873898 | 2.857715694 | 2.57 (2.28, 2.86) |

Bottom10_by_measure

| measure | location | EAPC | LCI | UCI | EAPC_95CI |
| --- | --- | --- | --- | --- | --- |
| DALYs (Disability-Adjusted Life Years) | Seychelles | -1.5055312 | -2.192794 | -0.813439204 | -1.51 (-2.19, -0.81) |
| DALYs (Disability-Adjusted Life Years) | Mauritius | -1.251898984 | -1.724356647 | -0.777169993 | -1.25 (-1.72, -0.78) |
| DALYs (Disability-Adjusted Life Years) | South Africa | -0.689801362 | -1.514100374 | 0.141396798 | -0.69 (-1.51, 0.14) |
| DALYs (Disability-Adjusted Life Years) | Libya | -0.54413182 | -1.057231645 | -0.028371149 | -0.54 (-1.06, -0.03) |
| DALYs (Disability-Adjusted Life Years) | Egypt | -0.533010907 | -1.464553852 | 0.407338739 | -0.53 (-1.46, 0.41) |
| DALYs (Disability-Adjusted Life Years) | Eswatini | -0.47989157 | -1.815244318 | 0.873622521 | -0.48 (-1.82, 0.87) |
| DALYs (Disability-Adjusted Life Years) | United Republic of Tanzania | -0.478333119 | -1.024652752 | 0.071002065 | -0.48 (-1.02, 0.07) |
| DALYs (Disability-Adjusted Life Years) | Tunisia | -0.308600697 | -1.0718798 | 0.460567481 | -0.31 (-1.07, 0.46) |
| DALYs (Disability-Adjusted Life Years) | Benin | -0.063460194 | -0.311978145 | 0.185677301 | -0.06 (-0.31, 0.19) |
| DALYs (Disability-Adjusted Life Years) | Zimbabwe | 0.122172207 | -0.653394036 | 0.903793042 | 0.12 (-0.65, 0.90) |
| Deaths | Seychelles | -1.466358622 | -2.176365435 | -0.751198559 | -1.47 (-2.18, -0.75) |
| Deaths | Mauritius | -1.268531661 | -1.792091021 | -0.742181137 | -1.27 (-1.79, -0.74) |
| Deaths | Tunisia | -0.695933493 | -1.327359494 | -0.06046687 | -0.70 (-1.33, -0.06) |
| Deaths | South Africa | -0.56851268 | -1.425208929 | 0.295628966 | -0.57 (-1.43, 0.30) |
| Deaths | Libya | -0.190516427 | -0.708588736 | 0.330259026 | -0.19 (-0.71, 0.33) |
| Deaths | Benin | -0.12846735 | -0.382108688 | 0.125819794 | -0.13 (-0.38, 0.13) |
| Deaths | Egypt | -0.085430595 | -1.009205537 | 0.846964947 | -0.09 (-1.01, 0.85) |
| Deaths | United Republic of Tanzania | -0.037608999 | -0.607321135 | 0.535368688 | -0.04 (-0.61, 0.54) |
| Deaths | Cabo Verde | 0.001984277 | -0.408606595 | 0.414267915 | 0.00 (-0.41, 0.41) |
| Deaths | Angola | 0.103294082 | -0.321702425 | 0.530102639 | 0.10 (-0.32, 0.53) |
| Incidence | Mauritius | -0.929441341 | -1.434592154 | -0.421701614 | -0.93 (-1.43, -0.42) |
| Incidence | Seychelles | -0.885324181 | -1.725325818 | -0.038142639 | -0.89 (-1.73, -0.04) |
| Incidence | Libya | -0.518228611 | -1.135989988 | 0.103392908 | -0.52 (-1.14, 0.10) |
| Incidence | South Africa | -0.206099293 | -1.08882027 | 0.684499421 | -0.21 (-1.09, 0.68) |
| Incidence | United Republic of Tanzania | 0.093267915 | -0.467666956 | 0.65736405 | 0.09 (-0.47, 0.66) |
| Incidence | Eswatini | 0.301987734 | -0.999426435 | 1.62050967 | 0.30 (-1.00, 1.62) |
| Incidence | Benin | 0.399502392 | 0.133066277 | 0.666647447 | 0.40 (0.13, 0.67) |
| Incidence | South Sudan | 0.464848521 | -0.064842456 | 0.997347044 | 0.46 (-0.06, 1.00) |
| Incidence | Egypt | 0.526295776 | -0.456287927 | 1.518578441 | 0.53 (-0.46, 1.52) |
| Incidence | Zimbabwe | 0.544094939 | -0.255202727 | 1.34979772 | 0.54 (-0.26, 1.35) |
| Prevalence | Libya | -1.422712111 | -2.03433503 | -0.807270685 | -1.42 (-2.03, -0.81) |
| Prevalence | Mauritius | -0.598900955 | -1.071675403 | -0.123867136 | -0.60 (-1.07, -0.12) |
| Prevalence | Seychelles | -0.114176017 | -0.917048776 | 0.695202449 | -0.11 (-0.92, 0.70) |
| Prevalence | South Sudan | 0.033105492 | -0.535263697 | 0.604722502 | 0.03 (-0.54, 0.60) |
| Prevalence | South Africa | 0.10755408 | -1.450469714 | 1.690209531 | 0.11 (-1.45, 1.69) |
| Prevalence | Eswatini | 0.143423764 | -1.045907958 | 1.347050093 | 0.14 (-1.05, 1.35) |
| Prevalence | Egypt | 0.192923936 | -0.616018172 | 1.008450479 | 0.19 (-0.62, 1.01) |
| Prevalence | United Republic of Tanzania | 0.19508081 | -0.127887765 | 0.519093808 | 0.20 (-0.13, 0.52) |
| Prevalence | Zimbabwe | 0.206091647 | 0.026484708 | 0.386021087 | 0.21 (0.03, 0.39) |
| Prevalence | Cabo Verde | 0.495157423 | 0.038729775 | 0.953667527 | 0.50 (0.04, 0.95) |
