# Supplementary material for: Bladder cancer in Africa, 2010–2023: burden, geographic heterogeneity, demographic drivers, pandemic-era temporal changes, and projections to 2030
Source: Infect Agent Cancer. 2026 Jul 23;21:69. doi: 10.1186/s13027-026-00782-3 (PMC13681883; doi:10.1186/s13027-026-00782-3)
Supplement: Supplementary file 3 [file 13027_2026_782_MOESM3_ESM.docx]

| **Country** | **Trajectory_Cluster** | **ASR_2010** | **ASR_2023** | **Absolute_Change** | **Relative_Change_Percent** |
| --- | --- | --- | --- | --- | --- |
| Botswana | Low-Stable Trajectory | 0.53 | 0.84 | 0.31 | 57.42 |
| Côte d'Ivoire | Low-Stable Trajectory | 2.02 | 3.15 | 1.13 | 56.29 |
| Morocco | Low-Stable Trajectory | 2.16 | 3.11 | 0.95 | 43.88 |
| Lesotho | Low-Stable Trajectory | 1.47 | 2.08 | 0.61 | 41.6 |
| Eritrea | Low-Stable Trajectory | 2.37 | 3.31 | 0.95 | 39.92 |
| Gambia | Low-Stable Trajectory | 0.88 | 1.22 | 0.34 | 38.73 |
| Guinea-Bissau | Low-Stable Trajectory | 2.12 | 2.81 | 0.69 | 32.4 |
| Togo | Low-Stable Trajectory | 2.25 | 2.96 | 0.71 | 31.75 |
| Guinea | Low-Stable Trajectory | 2.12 | 2.73 | 0.62 | 29.22 |
| Senegal | Low-Stable Trajectory | 2.11 | 2.71 | 0.6 | 28.59 |
| Central African Republic | Low-Stable Trajectory | 1.69 | 2.14 | 0.46 | 27.13 |
| Somalia | Low-Stable Trajectory | 1.15 | 1.42 | 0.28 | 24.13 |
| Nigeria | Low-Stable Trajectory | 1.55 | 1.87 | 0.32 | 20.69 |
| Namibia | Low-Stable Trajectory | 1.47 | 1.78 | 0.3 | 20.66 |
| Burkina Faso | Low-Stable Trajectory | 2.33 | 2.79 | 0.46 | 19.75 |
| Kenya | Low-Stable Trajectory | 1.41 | 1.68 | 0.27 | 19.21 |
| Niger | Low-Stable Trajectory | 2.53 | 2.92 | 0.39 | 15.38 |
| Comoros | Low-Stable Trajectory | 1.42 | 1.6 | 0.19 | 13.22 |
| Benin | Low-Stable Trajectory | 1.32 | 1.46 | 0.14 | 10.35 |
| Eswatini | Low-Stable Trajectory | 1.22 | 1.22 | 0 | 0.2 |
| Ethiopia | Moderate-Rising Trajectory | 3.3 | 5.33 | 2.03 | 61.68 |
| Democratic Republic of the Congo | Moderate-Rising Trajectory | 2.43 | 3.73 | 1.31 | 53.81 |
| Ghana | Moderate-Rising Trajectory | 2.99 | 4.45 | 1.46 | 48.97 |
| Gabon | Moderate-Rising Trajectory | 3.25 | 4.77 | 1.52 | 46.71 |
| Congo | Moderate-Rising Trajectory | 3.08 | 4.38 | 1.3 | 42.27 |
| Sierra Leone | Moderate-Rising Trajectory | 2.77 | 3.86 | 1.08 | 39.13 |
| Rwanda | Moderate-Rising Trajectory | 4.43 | 5.99 | 1.57 | 35.41 |
| Zambia | Moderate-Rising Trajectory | 4.49 | 6.06 | 1.57 | 34.96 |
| Equatorial Guinea | Moderate-Rising Trajectory | 4.88 | 6.57 | 1.7 | 34.8 |
| Algeria | Moderate-Rising Trajectory | 3.05 | 3.99 | 0.95 | 31.13 |
| Djibouti | Moderate-Rising Trajectory | 2.75 | 3.58 | 0.83 | 30.19 |
| Madagascar | Moderate-Rising Trajectory | 4.08 | 5.25 | 1.17 | 28.67 |
| Mauritania | Moderate-Rising Trajectory | 3.38 | 4.32 | 0.94 | 27.86 |
| Cameroon | Moderate-Rising Trajectory | 3.43 | 4.3 | 0.87 | 25.5 |
| Burundi | Moderate-Rising Trajectory | 2.75 | 3.45 | 0.7 | 25.35 |
| Cabo Verde | Moderate-Rising Trajectory | 2.77 | 3.46 | 0.68 | 24.59 |
| Chad | Moderate-Rising Trajectory | 2.78 | 3.41 | 0.63 | 22.69 |
| Uganda | Moderate-Rising Trajectory | 3.28 | 3.99 | 0.71 | 21.81 |
| Liberia | Moderate-Rising Trajectory | 3.29 | 4.01 | 0.71 | 21.7 |
| Sudan | Moderate-Rising Trajectory | 4.32 | 5.16 | 0.84 | 19.46 |
| Angola | Moderate-Rising Trajectory | 3 | 3.48 | 0.48 | 15.97 |
| South Sudan | Moderate-Rising Trajectory | 3.86 | 4.37 | 0.51 | 13.33 |
| Mozambique | Moderate-Rising Trajectory | 2.81 | 3.16 | 0.35 | 12.49 |
| South Africa | Moderate-Rising Trajectory | 3.49 | 3.85 | 0.36 | 10.38 |
| Zimbabwe | Moderate-Rising Trajectory | 4.42 | 4.6 | 0.18 | 4.08 |
| United Republic of Tanzania | Moderate-Rising Trajectory | 3.69 | 3.79 | 0.1 | 2.7 |
| Mauritius | Moderate-Rising Trajectory | 4.67 | 4.56 | -0.11 | -2.29 |
| Malawi | High-Persistent Trajectory | 7.73 | 10.59 | 2.86 | 37.02 |
| Mali | High-Persistent Trajectory | 7.3 | 9.01 | 1.71 | 23.35 |
| Sao Tome and Principe | High-Persistent Trajectory | 7.13 | 8.6 | 1.47 | 20.57 |
| Tunisia | High-Persistent Trajectory | 9.27 | 11.16 | 1.89 | 20.38 |
| Egypt | High-Persistent Trajectory | 7.87 | 9.17 | 1.3 | 16.51 |
| Seychelles | High-Persistent Trajectory | 8.38 | 8.34 | -0.04 | -0.46 |
| Libya | High-Persistent Trajectory | 11.12 | 10.49 | -0.62 | -5.61 |
