# Supplementary material for: Bladder cancer in Africa, 2010–2023: burden, geographic heterogeneity, demographic drivers, pandemic-era temporal changes, and projections to 2030
Source: Infect Agent Cancer. 2026 Jul 23;21:69. doi: 10.1186/s13027-026-00782-3 (PMC13681883; doi:10.1186/s13027-026-00782-3)
Supplement: Supplementary file 4 [file 13027_2026_782_MOESM4_ESM.docx]

| **location** | **region** | **N_start** | **N_end** | **change_total** | **effect_population** | **effect_aging_structure** | **effect_epidemiology** | **measure_display** | **measure_actual** | **diff_check** | **rank_type** | **rank** |
| --- | --- | --- | --- | --- | --- | --- | --- | --- | --- | --- | --- | --- |
| Egypt | North Africa | 3367.049578 | 6028.202761 | 2661.153183 | 1191.589074 | 917.7906676 | 551.7734413 | Incidence | Incidence | 0 | Total change (desc) | 1 |
| Ethiopia | Eastern Sub-Saharan Africa | 1042.918693 | 2656.503829 | 1613.585136 | 499.512365 | 224.9131476 | 889.1596232 | Incidence | Incidence | 0 | Total change (desc) | 2 |
| Democratic Republic of the Congo | Central Sub-Saharan Africa | 619.0070889 | 1475.997941 | 856.9908518 | 347.4466034 | 62.65132274 | 446.8929257 | Incidence | Incidence | 0 | Total change (desc) | 3 |
| South Africa | Southern Sub-Saharan Africa | 1241.553831 | 2049.487777 | 807.9339465 | 368.9575772 | 280.9154607 | 158.0609085 | Incidence | Incidence | 0 | Total change (desc) | 4 |
| Algeria | North Africa | 717.603892 | 1505.02003 | 787.4161377 | 215.8868439 | 274.84709 | 296.6822037 | Incidence | Incidence | 0 | Total change (desc) | 5 |
| Nigeria | Western Sub-Saharan Africa | 974.9532455 | 1743.234906 | 768.2816606 | 490.8929283 | 23.05258944 | 254.3361428 | Incidence | Incidence | 0 | Total change (desc) | 6 |
| Tunisia | North Africa | 829.9400543 | 1531.092221 | 701.1521665 | 115.5728304 | 348.9224228 | 236.6569133 | Incidence | Incidence | 0 | Total change (desc) | 7 |
| Morocco | North Africa | 471.0505405 | 1095.244829 | 624.1942881 | 70.01707424 | 276.7010653 | 277.4761486 | Incidence | Incidence | -1.13687E-13 | Total change (desc) | 8 |
| Malawi | Eastern Sub-Saharan Africa | 448.2878939 | 968.4864717 | 520.1985778 | 237.9703765 | 47.71512614 | 234.5130751 | Incidence | Incidence | -1.13687E-13 | Total change (desc) | 9 |
| Sudan | North Africa | 631.7564066 | 1144.568309 | 512.8119029 | 178.6276636 | 169.982512 | 164.2017273 | Incidence | Incidence | 0 | Total change (desc) | 10 |
| Ghana | Western Sub-Saharan Africa | 395.7505041 | 870.5992387 | 474.8487346 | 163.4481674 | 63.87680096 | 247.5237663 | Incidence | Incidence | 5.68434E-14 | Total change (desc) | 11 |
| Mali | Western Sub-Saharan Africa | 452.0920815 | 872.488061 | 420.3959795 | 262.8005522 | 12.34793988 | 145.2474874 | Incidence | Incidence | -5.68434E-14 | Total change (desc) | 12 |
| United Republic of Tanzania | Eastern Sub-Saharan Africa | 718.41496 | 1121.406422 | 402.991462 | 342.3322127 | 55.70398607 | 4.955263212 | Incidence | Incidence | 0 | Total change (desc) | 13 |
| Uganda | Eastern Sub-Saharan Africa | 365.6067326 | 714.4561944 | 348.8494619 | 170.9025127 | 61.84866931 | 116.0982798 | Incidence | Incidence | 0 | Total change (desc) | 14 |
| Madagascar | Eastern Sub-Saharan Africa | 323.9550133 | 649.4611704 | 325.5061571 | 165.6526925 | 29.32843494 | 130.5250297 | Incidence | Incidence | 0 | Total change (desc) | 15 |
| Cameroon | Central Sub-Saharan Africa | 276.9747758 | 566.3580805 | 289.3833047 | 171.6778082 | 12.42378634 | 105.2817102 | Incidence | Incidence | 0 | Total change (desc) | 16 |
| Rwanda | Eastern Sub-Saharan Africa | 182.8867416 | 449.5147385 | 266.6279969 | 84.69191069 | 77.47852202 | 104.4575642 | Incidence | Incidence | 0 | Total change (desc) | 17 |
| Côte d'Ivoire | Western Sub-Saharan Africa | 150.7762616 | 416.8739394 | 266.0976778 | 124.5747583 | 5.414980679 | 136.1079388 | Incidence | Incidence | 0 | Total change (desc) | 18 |
| Zambia | Eastern Sub-Saharan Africa | 201.6870828 | 452.2517401 | 250.5646573 | 140.857193 | 16.62221819 | 93.08524609 | Incidence | Incidence | 0 | Total change (desc) | 19 |
| Angola | Central Sub-Saharan Africa | 223.5026224 | 464.9090857 | 241.4064632 | 150.9616646 | 31.87298424 | 58.57181437 | Incidence | Incidence | -2.84217E-14 | Total change (desc) | 20 |
| Kenya | Eastern Sub-Saharan Africa | 237.48015 | 466.3540824 | 228.8739325 | 81.41782665 | 84.30224443 | 63.15386137 | Incidence | Incidence | 0 | Total change (desc) | 21 |
| Libya | North Africa | 382.31982 | 593.796733 | 211.476913 | 81.40002924 | 162.866347 | -32.78946329 | Incidence | Incidence | 0 | Total change (desc) | 22 |
| Mozambique | Eastern Sub-Saharan Africa | 268.1066567 | 402.9028683 | 134.7962116 | 120.7949153 | -13.11218603 | 27.11348231 | Incidence | Incidence | 0 | Total change (desc) | 23 |
| Zimbabwe | Southern Sub-Saharan Africa | 283.8878896 | 410.8919649 | 127.0040753 | 62.46317884 | 62.56372893 | 1.9771675 | Incidence | Incidence | 0 | Total change (desc) | 24 |
| Burkina Faso | Western Sub-Saharan Africa | 155.506192 | 272.3669682 | 116.8607762 | 75.60371026 | 5.709710735 | 35.54735521 | Incidence | Incidence | 0 | Total change (desc) | 25 |
| Senegal | Western Sub-Saharan Africa | 123.2812039 | 239.6781262 | 116.3969223 | 69.45142814 | 1.211692835 | 45.73380133 | Incidence | Incidence | 0 | Total change (desc) | 26 |
| Niger | Western Sub-Saharan Africa | 118.8153946 | 230.1798049 | 111.3644103 | 81.83716626 | 1.984076089 | 27.5431679 | Incidence | Incidence | -1.42109E-14 | Total change (desc) | 27 |
| Burundi | Eastern Sub-Saharan Africa | 93.63631324 | 196.0972405 | 102.4609273 | 58.85155319 | 4.563255958 | 39.04611814 | Incidence | Incidence | 1.42109E-14 | Total change (desc) | 28 |
| Chad | Central Sub-Saharan Africa | 111.6779669 | 199.5444284 | 87.86646153 | 74.16993224 | -17.48423476 | 31.18076405 | Incidence | Incidence | 0 | Total change (desc) | 29 |
| Togo | Western Sub-Saharan Africa | 55.90637987 | 126.4710203 | 70.56464039 | 24.96842551 | 19.38258348 | 26.2136314 | Incidence | Incidence | 0 | Total change (desc) | 30 |
| South Sudan | Eastern Sub-Saharan Africa | 129.3486379 | 198.3292364 | 68.98059856 | 9.538804703 | 36.10575438 | 23.33603948 | Incidence | Incidence | 0 | Total change (desc) | 31 |
| Eritrea | Eastern Sub-Saharan Africa | 63.92276195 | 131.453102 | 67.53034006 | 23.57046225 | 11.21897664 | 32.74090116 | Incidence | Incidence | 0 | Total change (desc) | 32 |
| Somalia | Eastern Sub-Saharan Africa | 65.20927724 | 130.772374 | 65.56309677 | 40.90003982 | 3.76154567 | 20.90151128 | Incidence | Incidence | 0 | Total change (desc) | 33 |
| Guinea | Western Sub-Saharan Africa | 104.0115759 | 165.4458229 | 61.43424698 | 46.32725862 | -18.53988635 | 33.64687471 | Incidence | Incidence | 0 | Total change (desc) | 34 |
| Congo | Central Sub-Saharan Africa | 53.74178904 | 111.6579722 | 57.91618316 | 28.38697991 | 1.667738299 | 27.86146495 | Incidence | Incidence | -7.10543E-15 | Total change (desc) | 35 |
| Sierra Leone | Western Sub-Saharan Africa | 83.62864391 | 139.2027744 | 55.57413045 | 26.64922273 | -8.527665321 | 37.45257305 | Incidence | Incidence | 0 | Total change (desc) | 36 |
| Liberia | Western Sub-Saharan Africa | 52.84883346 | 102.9044594 | 50.0556259 | 26.96741359 | 5.343403615 | 17.7448087 | Incidence | Incidence | 0 | Total change (desc) | 37 |
| Mauritania | Western Sub-Saharan Africa | 51.39508696 | 97.35518072 | 45.96009375 | 22.11129513 | 5.481238367 | 18.36756026 | Incidence | Incidence | 0 | Total change (desc) | 38 |
| Benin | Western Sub-Saharan Africa | 47.05118146 | 83.67818684 | 36.62700537 | 25.8761262 | 4.32378786 | 6.427091315 | Incidence | Incidence | 0 | Total change (desc) | 39 |
| Gabon | Central Sub-Saharan Africa | 28.15469381 | 57.96037753 | 29.80568372 | 10.00543098 | 4.07969374 | 15.720559 | Incidence | Incidence | -3.55271E-15 | Total change (desc) | 40 |
| Central African Republic | Central Sub-Saharan Africa | 28.61050162 | 53.30204079 | 24.69153917 | 12.14605707 | 2.396578433 | 10.14890366 | Incidence | Incidence | 3.55271E-15 | Total change (desc) | 41 |
| Mauritius | Eastern Sub-Saharan Africa | 60.19161675 | 84.02221324 | 23.83059649 | 0.011342292 | 25.66054451 | -1.841290313 | Incidence | Incidence | 3.55271E-15 | Total change (desc) | 42 |
| Equatorial Guinea | Central Sub-Saharan Africa | 16.25149499 | 36.67667409 | 20.42517911 | 11.39915094 | 0.902493832 | 8.123534332 | Incidence | Incidence | 0 | Total change (desc) | 43 |
| Djibouti | Eastern Sub-Saharan Africa | 11.0692782 | 28.61447115 | 17.54519294 | 7.817567031 | 3.88739226 | 5.840233652 | Incidence | Incidence | 0 | Total change (desc) | 44 |
| Namibia | Southern Sub-Saharan Africa | 17.70440285 | 33.03275073 | 15.32834788 | 8.414433054 | 1.930204778 | 4.983710051 | Incidence | Incidence | 0 | Total change (desc) | 45 |
| Lesotho | Southern Sub-Saharan Africa | 15.92881369 | 26.95036111 | 11.02154742 | 0.800833023 | 3.141576313 | 7.07913808 | Incidence | Incidence | 0 | Total change (desc) | 46 |
| Guinea-Bissau | Western Sub-Saharan Africa | 11.87648763 | 22.53876942 | 10.6622818 | 5.264156748 | 0.259410561 | 5.13871449 | Incidence | Incidence | 0 | Total change (desc) | 47 |
| Botswana | Southern Sub-Saharan Africa | 6.202745607 | 14.80455431 | 8.601808704 | 1.826308112 | 2.248030224 | 4.527470368 | Incidence | Incidence | 0 | Total change (desc) | 48 |
| Gambia | Western Sub-Saharan Africa | 6.675622025 | 13.55162437 | 6.876002348 | 3.336254125 | 0.323289649 | 3.216458574 | Incidence | Incidence | 8.88178E-16 | Total change (desc) | 49 |
| Cabo Verde | Western Sub-Saharan Africa | 10.15281093 | 15.79540173 | 5.642590794 | -0.488964256 | 3.064073968 | 3.067481082 | Incidence | Incidence | 0 | Total change (desc) | 50 |
| Sao Tome and Principe | Central Sub-Saharan Africa | 6.132713872 | 11.02063118 | 4.887917308 | 1.862372146 | 1.226636111 | 1.798909051 | Incidence | Incidence | 0 | Total change (desc) | 51 |
| Comoros | Eastern Sub-Saharan Africa | 5.780427965 | 9.50207455 | 3.721646585 | 2.064926462 | 0.818329906 | 0.838390217 | Incidence | Incidence | 0 | Total change (desc) | 52 |
| Seychelles | Eastern Sub-Saharan Africa | 7.138248913 | 10.33984684 | 3.201597929 | 1.031709925 | 2.182325008 | -0.012437003 | Incidence | Incidence | -4.44089E-16 | Total change (desc) | 53 |
| Eswatini | Southern Sub-Saharan Africa | 7.311386974 | 9.708305634 | 2.39691866 | 0.734342191 | 1.965613019 | -0.30303655 | Incidence | Incidence | 0 | Total change (desc) | 54 |
| Ethiopia | Eastern Sub-Saharan Africa | 1042.918693 | 2656.503829 | 1613.585136 | 499.512365 | 224.9131476 | 889.1596232 | Incidence | Incidence | 0 | Epidemiology (desc) | 1 |
| Egypt | North Africa | 3367.049578 | 6028.202761 | 2661.153183 | 1191.589074 | 917.7906676 | 551.7734413 | Incidence | Incidence | 0 | Epidemiology (desc) | 2 |
| Democratic Republic of the Congo | Central Sub-Saharan Africa | 619.0070889 | 1475.997941 | 856.9908518 | 347.4466034 | 62.65132274 | 446.8929257 | Incidence | Incidence | 0 | Epidemiology (desc) | 3 |
| Algeria | North Africa | 717.603892 | 1505.02003 | 787.4161377 | 215.8868439 | 274.84709 | 296.6822037 | Incidence | Incidence | 0 | Epidemiology (desc) | 4 |
| Morocco | North Africa | 471.0505405 | 1095.244829 | 624.1942881 | 70.01707424 | 276.7010653 | 277.4761486 | Incidence | Incidence | -1.13687E-13 | Epidemiology (desc) | 5 |
| Nigeria | Western Sub-Saharan Africa | 974.9532455 | 1743.234906 | 768.2816606 | 490.8929283 | 23.05258944 | 254.3361428 | Incidence | Incidence | 0 | Epidemiology (desc) | 6 |
| Ghana | Western Sub-Saharan Africa | 395.7505041 | 870.5992387 | 474.8487346 | 163.4481674 | 63.87680096 | 247.5237663 | Incidence | Incidence | 5.68434E-14 | Epidemiology (desc) | 7 |
| Tunisia | North Africa | 829.9400543 | 1531.092221 | 701.1521665 | 115.5728304 | 348.9224228 | 236.6569133 | Incidence | Incidence | 0 | Epidemiology (desc) | 8 |
| Malawi | Eastern Sub-Saharan Africa | 448.2878939 | 968.4864717 | 520.1985778 | 237.9703765 | 47.71512614 | 234.5130751 | Incidence | Incidence | -1.13687E-13 | Epidemiology (desc) | 9 |
| Sudan | North Africa | 631.7564066 | 1144.568309 | 512.8119029 | 178.6276636 | 169.982512 | 164.2017273 | Incidence | Incidence | 0 | Epidemiology (desc) | 10 |
| South Africa | Southern Sub-Saharan Africa | 1241.553831 | 2049.487777 | 807.9339465 | 368.9575772 | 280.9154607 | 158.0609085 | Incidence | Incidence | 0 | Epidemiology (desc) | 11 |
| Mali | Western Sub-Saharan Africa | 452.0920815 | 872.488061 | 420.3959795 | 262.8005522 | 12.34793988 | 145.2474874 | Incidence | Incidence | -5.68434E-14 | Epidemiology (desc) | 12 |
| Côte d'Ivoire | Western Sub-Saharan Africa | 150.7762616 | 416.8739394 | 266.0976778 | 124.5747583 | 5.414980679 | 136.1079388 | Incidence | Incidence | 0 | Epidemiology (desc) | 13 |
| Madagascar | Eastern Sub-Saharan Africa | 323.9550133 | 649.4611704 | 325.5061571 | 165.6526925 | 29.32843494 | 130.5250297 | Incidence | Incidence | 0 | Epidemiology (desc) | 14 |
| Uganda | Eastern Sub-Saharan Africa | 365.6067326 | 714.4561944 | 348.8494619 | 170.9025127 | 61.84866931 | 116.0982798 | Incidence | Incidence | 0 | Epidemiology (desc) | 15 |
| Cameroon | Central Sub-Saharan Africa | 276.9747758 | 566.3580805 | 289.3833047 | 171.6778082 | 12.42378634 | 105.2817102 | Incidence | Incidence | 0 | Epidemiology (desc) | 16 |
| Rwanda | Eastern Sub-Saharan Africa | 182.8867416 | 449.5147385 | 266.6279969 | 84.69191069 | 77.47852202 | 104.4575642 | Incidence | Incidence | 0 | Epidemiology (desc) | 17 |
| Zambia | Eastern Sub-Saharan Africa | 201.6870828 | 452.2517401 | 250.5646573 | 140.857193 | 16.62221819 | 93.08524609 | Incidence | Incidence | 0 | Epidemiology (desc) | 18 |
| Kenya | Eastern Sub-Saharan Africa | 237.48015 | 466.3540824 | 228.8739325 | 81.41782665 | 84.30224443 | 63.15386137 | Incidence | Incidence | 0 | Epidemiology (desc) | 19 |
| Angola | Central Sub-Saharan Africa | 223.5026224 | 464.9090857 | 241.4064632 | 150.9616646 | 31.87298424 | 58.57181437 | Incidence | Incidence | -2.84217E-14 | Epidemiology (desc) | 20 |
| Senegal | Western Sub-Saharan Africa | 123.2812039 | 239.6781262 | 116.3969223 | 69.45142814 | 1.211692835 | 45.73380133 | Incidence | Incidence | 0 | Epidemiology (desc) | 21 |
| Burundi | Eastern Sub-Saharan Africa | 93.63631324 | 196.0972405 | 102.4609273 | 58.85155319 | 4.563255958 | 39.04611814 | Incidence | Incidence | 1.42109E-14 | Epidemiology (desc) | 22 |
| Sierra Leone | Western Sub-Saharan Africa | 83.62864391 | 139.2027744 | 55.57413045 | 26.64922273 | -8.527665321 | 37.45257305 | Incidence | Incidence | 0 | Epidemiology (desc) | 23 |
| Burkina Faso | Western Sub-Saharan Africa | 155.506192 | 272.3669682 | 116.8607762 | 75.60371026 | 5.709710735 | 35.54735521 | Incidence | Incidence | 0 | Epidemiology (desc) | 24 |
| Guinea | Western Sub-Saharan Africa | 104.0115759 | 165.4458229 | 61.43424698 | 46.32725862 | -18.53988635 | 33.64687471 | Incidence | Incidence | 0 | Epidemiology (desc) | 25 |
| Eritrea | Eastern Sub-Saharan Africa | 63.92276195 | 131.453102 | 67.53034006 | 23.57046225 | 11.21897664 | 32.74090116 | Incidence | Incidence | 0 | Epidemiology (desc) | 26 |
| Chad | Central Sub-Saharan Africa | 111.6779669 | 199.5444284 | 87.86646153 | 74.16993224 | -17.48423476 | 31.18076405 | Incidence | Incidence | 0 | Epidemiology (desc) | 27 |
| Congo | Central Sub-Saharan Africa | 53.74178904 | 111.6579722 | 57.91618316 | 28.38697991 | 1.667738299 | 27.86146495 | Incidence | Incidence | -7.10543E-15 | Epidemiology (desc) | 28 |
| Niger | Western Sub-Saharan Africa | 118.8153946 | 230.1798049 | 111.3644103 | 81.83716626 | 1.984076089 | 27.5431679 | Incidence | Incidence | -1.42109E-14 | Epidemiology (desc) | 29 |
| Mozambique | Eastern Sub-Saharan Africa | 268.1066567 | 402.9028683 | 134.7962116 | 120.7949153 | -13.11218603 | 27.11348231 | Incidence | Incidence | 0 | Epidemiology (desc) | 30 |
| Togo | Western Sub-Saharan Africa | 55.90637987 | 126.4710203 | 70.56464039 | 24.96842551 | 19.38258348 | 26.2136314 | Incidence | Incidence | 0 | Epidemiology (desc) | 31 |
| South Sudan | Eastern Sub-Saharan Africa | 129.3486379 | 198.3292364 | 68.98059856 | 9.538804703 | 36.10575438 | 23.33603948 | Incidence | Incidence | 0 | Epidemiology (desc) | 32 |
| Somalia | Eastern Sub-Saharan Africa | 65.20927724 | 130.772374 | 65.56309677 | 40.90003982 | 3.76154567 | 20.90151128 | Incidence | Incidence | 0 | Epidemiology (desc) | 33 |
| Mauritania | Western Sub-Saharan Africa | 51.39508696 | 97.35518072 | 45.96009375 | 22.11129513 | 5.481238367 | 18.36756026 | Incidence | Incidence | 0 | Epidemiology (desc) | 34 |
| Liberia | Western Sub-Saharan Africa | 52.84883346 | 102.9044594 | 50.0556259 | 26.96741359 | 5.343403615 | 17.7448087 | Incidence | Incidence | 0 | Epidemiology (desc) | 35 |
| Gabon | Central Sub-Saharan Africa | 28.15469381 | 57.96037753 | 29.80568372 | 10.00543098 | 4.07969374 | 15.720559 | Incidence | Incidence | -3.55271E-15 | Epidemiology (desc) | 36 |
| Central African Republic | Central Sub-Saharan Africa | 28.61050162 | 53.30204079 | 24.69153917 | 12.14605707 | 2.396578433 | 10.14890366 | Incidence | Incidence | 3.55271E-15 | Epidemiology (desc) | 37 |
| Equatorial Guinea | Central Sub-Saharan Africa | 16.25149499 | 36.67667409 | 20.42517911 | 11.39915094 | 0.902493832 | 8.123534332 | Incidence | Incidence | 0 | Epidemiology (desc) | 38 |
| Lesotho | Southern Sub-Saharan Africa | 15.92881369 | 26.95036111 | 11.02154742 | 0.800833023 | 3.141576313 | 7.07913808 | Incidence | Incidence | 0 | Epidemiology (desc) | 39 |
| Benin | Western Sub-Saharan Africa | 47.05118146 | 83.67818684 | 36.62700537 | 25.8761262 | 4.32378786 | 6.427091315 | Incidence | Incidence | 0 | Epidemiology (desc) | 40 |
| Djibouti | Eastern Sub-Saharan Africa | 11.0692782 | 28.61447115 | 17.54519294 | 7.817567031 | 3.88739226 | 5.840233652 | Incidence | Incidence | 0 | Epidemiology (desc) | 41 |
| Guinea-Bissau | Western Sub-Saharan Africa | 11.87648763 | 22.53876942 | 10.6622818 | 5.264156748 | 0.259410561 | 5.13871449 | Incidence | Incidence | 0 | Epidemiology (desc) | 42 |
| Namibia | Southern Sub-Saharan Africa | 17.70440285 | 33.03275073 | 15.32834788 | 8.414433054 | 1.930204778 | 4.983710051 | Incidence | Incidence | 0 | Epidemiology (desc) | 43 |
| United Republic of Tanzania | Eastern Sub-Saharan Africa | 718.41496 | 1121.406422 | 402.991462 | 342.3322127 | 55.70398607 | 4.955263212 | Incidence | Incidence | 0 | Epidemiology (desc) | 44 |
| Botswana | Southern Sub-Saharan Africa | 6.202745607 | 14.80455431 | 8.601808704 | 1.826308112 | 2.248030224 | 4.527470368 | Incidence | Incidence | 0 | Epidemiology (desc) | 45 |
| Gambia | Western Sub-Saharan Africa | 6.675622025 | 13.55162437 | 6.876002348 | 3.336254125 | 0.323289649 | 3.216458574 | Incidence | Incidence | 8.88178E-16 | Epidemiology (desc) | 46 |
| Cabo Verde | Western Sub-Saharan Africa | 10.15281093 | 15.79540173 | 5.642590794 | -0.488964256 | 3.064073968 | 3.067481082 | Incidence | Incidence | 0 | Epidemiology (desc) | 47 |
| Zimbabwe | Southern Sub-Saharan Africa | 283.8878896 | 410.8919649 | 127.0040753 | 62.46317884 | 62.56372893 | 1.9771675 | Incidence | Incidence | 0 | Epidemiology (desc) | 48 |
| Sao Tome and Principe | Central Sub-Saharan Africa | 6.132713872 | 11.02063118 | 4.887917308 | 1.862372146 | 1.226636111 | 1.798909051 | Incidence | Incidence | 0 | Epidemiology (desc) | 49 |
| Comoros | Eastern Sub-Saharan Africa | 5.780427965 | 9.50207455 | 3.721646585 | 2.064926462 | 0.818329906 | 0.838390217 | Incidence | Incidence | 0 | Epidemiology (desc) | 50 |
| Seychelles | Eastern Sub-Saharan Africa | 7.138248913 | 10.33984684 | 3.201597929 | 1.031709925 | 2.182325008 | -0.012437003 | Incidence | Incidence | -4.44089E-16 | Epidemiology (desc) | 51 |
| Eswatini | Southern Sub-Saharan Africa | 7.311386974 | 9.708305634 | 2.39691866 | 0.734342191 | 1.965613019 | -0.30303655 | Incidence | Incidence | 0 | Epidemiology (desc) | 52 |
| Mauritius | Eastern Sub-Saharan Africa | 60.19161675 | 84.02221324 | 23.83059649 | 0.011342292 | 25.66054451 | -1.841290313 | Incidence | Incidence | 3.55271E-15 | Epidemiology (desc) | 53 |
| Libya | North Africa | 382.31982 | 593.796733 | 211.476913 | 81.40002924 | 162.866347 | -32.78946329 | Incidence | Incidence | 0 | Epidemiology (desc) | 54 |
| Egypt | North Africa | 3367.049578 | 6028.202761 | 2661.153183 | 1191.589074 | 917.7906676 | 551.7734413 | Incidence | Incidence | 0 | Aging/Structure (desc) | 1 |
| Tunisia | North Africa | 829.9400543 | 1531.092221 | 701.1521665 | 115.5728304 | 348.9224228 | 236.6569133 | Incidence | Incidence | 0 | Aging/Structure (desc) | 2 |
| South Africa | Southern Sub-Saharan Africa | 1241.553831 | 2049.487777 | 807.9339465 | 368.9575772 | 280.9154607 | 158.0609085 | Incidence | Incidence | 0 | Aging/Structure (desc) | 3 |
| Morocco | North Africa | 471.0505405 | 1095.244829 | 624.1942881 | 70.01707424 | 276.7010653 | 277.4761486 | Incidence | Incidence | -1.13687E-13 | Aging/Structure (desc) | 4 |
| Algeria | North Africa | 717.603892 | 1505.02003 | 787.4161377 | 215.8868439 | 274.84709 | 296.6822037 | Incidence | Incidence | 0 | Aging/Structure (desc) | 5 |
| Ethiopia | Eastern Sub-Saharan Africa | 1042.918693 | 2656.503829 | 1613.585136 | 499.512365 | 224.9131476 | 889.1596232 | Incidence | Incidence | 0 | Aging/Structure (desc) | 6 |
| Sudan | North Africa | 631.7564066 | 1144.568309 | 512.8119029 | 178.6276636 | 169.982512 | 164.2017273 | Incidence | Incidence | 0 | Aging/Structure (desc) | 7 |
| Libya | North Africa | 382.31982 | 593.796733 | 211.476913 | 81.40002924 | 162.866347 | -32.78946329 | Incidence | Incidence | 0 | Aging/Structure (desc) | 8 |
| Kenya | Eastern Sub-Saharan Africa | 237.48015 | 466.3540824 | 228.8739325 | 81.41782665 | 84.30224443 | 63.15386137 | Incidence | Incidence | 0 | Aging/Structure (desc) | 9 |
| Rwanda | Eastern Sub-Saharan Africa | 182.8867416 | 449.5147385 | 266.6279969 | 84.69191069 | 77.47852202 | 104.4575642 | Incidence | Incidence | 0 | Aging/Structure (desc) | 10 |
| Ghana | Western Sub-Saharan Africa | 395.7505041 | 870.5992387 | 474.8487346 | 163.4481674 | 63.87680096 | 247.5237663 | Incidence | Incidence | 5.68434E-14 | Aging/Structure (desc) | 11 |
| Democratic Republic of the Congo | Central Sub-Saharan Africa | 619.0070889 | 1475.997941 | 856.9908518 | 347.4466034 | 62.65132274 | 446.8929257 | Incidence | Incidence | 0 | Aging/Structure (desc) | 12 |
| Zimbabwe | Southern Sub-Saharan Africa | 283.8878896 | 410.8919649 | 127.0040753 | 62.46317884 | 62.56372893 | 1.9771675 | Incidence | Incidence | 0 | Aging/Structure (desc) | 13 |
| Uganda | Eastern Sub-Saharan Africa | 365.6067326 | 714.4561944 | 348.8494619 | 170.9025127 | 61.84866931 | 116.0982798 | Incidence | Incidence | 0 | Aging/Structure (desc) | 14 |
| United Republic of Tanzania | Eastern Sub-Saharan Africa | 718.41496 | 1121.406422 | 402.991462 | 342.3322127 | 55.70398607 | 4.955263212 | Incidence | Incidence | 0 | Aging/Structure (desc) | 15 |
| Malawi | Eastern Sub-Saharan Africa | 448.2878939 | 968.4864717 | 520.1985778 | 237.9703765 | 47.71512614 | 234.5130751 | Incidence | Incidence | -1.13687E-13 | Aging/Structure (desc) | 16 |
| South Sudan | Eastern Sub-Saharan Africa | 129.3486379 | 198.3292364 | 68.98059856 | 9.538804703 | 36.10575438 | 23.33603948 | Incidence | Incidence | 0 | Aging/Structure (desc) | 17 |
| Angola | Central Sub-Saharan Africa | 223.5026224 | 464.9090857 | 241.4064632 | 150.9616646 | 31.87298424 | 58.57181437 | Incidence | Incidence | -2.84217E-14 | Aging/Structure (desc) | 18 |
| Madagascar | Eastern Sub-Saharan Africa | 323.9550133 | 649.4611704 | 325.5061571 | 165.6526925 | 29.32843494 | 130.5250297 | Incidence | Incidence | 0 | Aging/Structure (desc) | 19 |
| Mauritius | Eastern Sub-Saharan Africa | 60.19161675 | 84.02221324 | 23.83059649 | 0.011342292 | 25.66054451 | -1.841290313 | Incidence | Incidence | 3.55271E-15 | Aging/Structure (desc) | 20 |
| Nigeria | Western Sub-Saharan Africa | 974.9532455 | 1743.234906 | 768.2816606 | 490.8929283 | 23.05258944 | 254.3361428 | Incidence | Incidence | 0 | Aging/Structure (desc) | 21 |
| Togo | Western Sub-Saharan Africa | 55.90637987 | 126.4710203 | 70.56464039 | 24.96842551 | 19.38258348 | 26.2136314 | Incidence | Incidence | 0 | Aging/Structure (desc) | 22 |
| Zambia | Eastern Sub-Saharan Africa | 201.6870828 | 452.2517401 | 250.5646573 | 140.857193 | 16.62221819 | 93.08524609 | Incidence | Incidence | 0 | Aging/Structure (desc) | 23 |
| Cameroon | Central Sub-Saharan Africa | 276.9747758 | 566.3580805 | 289.3833047 | 171.6778082 | 12.42378634 | 105.2817102 | Incidence | Incidence | 0 | Aging/Structure (desc) | 24 |
| Mali | Western Sub-Saharan Africa | 452.0920815 | 872.488061 | 420.3959795 | 262.8005522 | 12.34793988 | 145.2474874 | Incidence | Incidence | -5.68434E-14 | Aging/Structure (desc) | 25 |
| Eritrea | Eastern Sub-Saharan Africa | 63.92276195 | 131.453102 | 67.53034006 | 23.57046225 | 11.21897664 | 32.74090116 | Incidence | Incidence | 0 | Aging/Structure (desc) | 26 |
| Burkina Faso | Western Sub-Saharan Africa | 155.506192 | 272.3669682 | 116.8607762 | 75.60371026 | 5.709710735 | 35.54735521 | Incidence | Incidence | 0 | Aging/Structure (desc) | 27 |
| Mauritania | Western Sub-Saharan Africa | 51.39508696 | 97.35518072 | 45.96009375 | 22.11129513 | 5.481238367 | 18.36756026 | Incidence | Incidence | 0 | Aging/Structure (desc) | 28 |
| Côte d'Ivoire | Western Sub-Saharan Africa | 150.7762616 | 416.8739394 | 266.0976778 | 124.5747583 | 5.414980679 | 136.1079388 | Incidence | Incidence | 0 | Aging/Structure (desc) | 29 |
| Liberia | Western Sub-Saharan Africa | 52.84883346 | 102.9044594 | 50.0556259 | 26.96741359 | 5.343403615 | 17.7448087 | Incidence | Incidence | 0 | Aging/Structure (desc) | 30 |
| Burundi | Eastern Sub-Saharan Africa | 93.63631324 | 196.0972405 | 102.4609273 | 58.85155319 | 4.563255958 | 39.04611814 | Incidence | Incidence | 1.42109E-14 | Aging/Structure (desc) | 31 |
| Benin | Western Sub-Saharan Africa | 47.05118146 | 83.67818684 | 36.62700537 | 25.8761262 | 4.32378786 | 6.427091315 | Incidence | Incidence | 0 | Aging/Structure (desc) | 32 |
| Gabon | Central Sub-Saharan Africa | 28.15469381 | 57.96037753 | 29.80568372 | 10.00543098 | 4.07969374 | 15.720559 | Incidence | Incidence | -3.55271E-15 | Aging/Structure (desc) | 33 |
| Djibouti | Eastern Sub-Saharan Africa | 11.0692782 | 28.61447115 | 17.54519294 | 7.817567031 | 3.88739226 | 5.840233652 | Incidence | Incidence | 0 | Aging/Structure (desc) | 34 |
| Somalia | Eastern Sub-Saharan Africa | 65.20927724 | 130.772374 | 65.56309677 | 40.90003982 | 3.76154567 | 20.90151128 | Incidence | Incidence | 0 | Aging/Structure (desc) | 35 |
| Lesotho | Southern Sub-Saharan Africa | 15.92881369 | 26.95036111 | 11.02154742 | 0.800833023 | 3.141576313 | 7.07913808 | Incidence | Incidence | 0 | Aging/Structure (desc) | 36 |
| Cabo Verde | Western Sub-Saharan Africa | 10.15281093 | 15.79540173 | 5.642590794 | -0.488964256 | 3.064073968 | 3.067481082 | Incidence | Incidence | 0 | Aging/Structure (desc) | 37 |
| Central African Republic | Central Sub-Saharan Africa | 28.61050162 | 53.30204079 | 24.69153917 | 12.14605707 | 2.396578433 | 10.14890366 | Incidence | Incidence | 3.55271E-15 | Aging/Structure (desc) | 38 |
| Botswana | Southern Sub-Saharan Africa | 6.202745607 | 14.80455431 | 8.601808704 | 1.826308112 | 2.248030224 | 4.527470368 | Incidence | Incidence | 0 | Aging/Structure (desc) | 39 |
| Seychelles | Eastern Sub-Saharan Africa | 7.138248913 | 10.33984684 | 3.201597929 | 1.031709925 | 2.182325008 | -0.012437003 | Incidence | Incidence | -4.44089E-16 | Aging/Structure (desc) | 40 |
| Niger | Western Sub-Saharan Africa | 118.8153946 | 230.1798049 | 111.3644103 | 81.83716626 | 1.984076089 | 27.5431679 | Incidence | Incidence | -1.42109E-14 | Aging/Structure (desc) | 41 |
| Eswatini | Southern Sub-Saharan Africa | 7.311386974 | 9.708305634 | 2.39691866 | 0.734342191 | 1.965613019 | -0.30303655 | Incidence | Incidence | 0 | Aging/Structure (desc) | 42 |
| Namibia | Southern Sub-Saharan Africa | 17.70440285 | 33.03275073 | 15.32834788 | 8.414433054 | 1.930204778 | 4.983710051 | Incidence | Incidence | 0 | Aging/Structure (desc) | 43 |
| Congo | Central Sub-Saharan Africa | 53.74178904 | 111.6579722 | 57.91618316 | 28.38697991 | 1.667738299 | 27.86146495 | Incidence | Incidence | -7.10543E-15 | Aging/Structure (desc) | 44 |
| Sao Tome and Principe | Central Sub-Saharan Africa | 6.132713872 | 11.02063118 | 4.887917308 | 1.862372146 | 1.226636111 | 1.798909051 | Incidence | Incidence | 0 | Aging/Structure (desc) | 45 |
| Senegal | Western Sub-Saharan Africa | 123.2812039 | 239.6781262 | 116.3969223 | 69.45142814 | 1.211692835 | 45.73380133 | Incidence | Incidence | 0 | Aging/Structure (desc) | 46 |
| Equatorial Guinea | Central Sub-Saharan Africa | 16.25149499 | 36.67667409 | 20.42517911 | 11.39915094 | 0.902493832 | 8.123534332 | Incidence | Incidence | 0 | Aging/Structure (desc) | 47 |
| Comoros | Eastern Sub-Saharan Africa | 5.780427965 | 9.50207455 | 3.721646585 | 2.064926462 | 0.818329906 | 0.838390217 | Incidence | Incidence | 0 | Aging/Structure (desc) | 48 |
| Gambia | Western Sub-Saharan Africa | 6.675622025 | 13.55162437 | 6.876002348 | 3.336254125 | 0.323289649 | 3.216458574 | Incidence | Incidence | 8.88178E-16 | Aging/Structure (desc) | 49 |
| Guinea-Bissau | Western Sub-Saharan Africa | 11.87648763 | 22.53876942 | 10.6622818 | 5.264156748 | 0.259410561 | 5.13871449 | Incidence | Incidence | 0 | Aging/Structure (desc) | 50 |
| Sierra Leone | Western Sub-Saharan Africa | 83.62864391 | 139.2027744 | 55.57413045 | 26.64922273 | -8.527665321 | 37.45257305 | Incidence | Incidence | 0 | Aging/Structure (desc) | 51 |
| Mozambique | Eastern Sub-Saharan Africa | 268.1066567 | 402.9028683 | 134.7962116 | 120.7949153 | -13.11218603 | 27.11348231 | Incidence | Incidence | 0 | Aging/Structure (desc) | 52 |
| Chad | Central Sub-Saharan Africa | 111.6779669 | 199.5444284 | 87.86646153 | 74.16993224 | -17.48423476 | 31.18076405 | Incidence | Incidence | 0 | Aging/Structure (desc) | 53 |
| Guinea | Western Sub-Saharan Africa | 104.0115759 | 165.4458229 | 61.43424698 | 46.32725862 | -18.53988635 | 33.64687471 | Incidence | Incidence | 0 | Aging/Structure (desc) | 54 |
| Egypt | North Africa | 3367.049578 | 6028.202761 | 2661.153183 | 1191.589074 | 917.7906676 | 551.7734413 | Incidence | Incidence | 0 | Population (desc) | 1 |
| Ethiopia | Eastern Sub-Saharan Africa | 1042.918693 | 2656.503829 | 1613.585136 | 499.512365 | 224.9131476 | 889.1596232 | Incidence | Incidence | 0 | Population (desc) | 2 |
| Nigeria | Western Sub-Saharan Africa | 974.9532455 | 1743.234906 | 768.2816606 | 490.8929283 | 23.05258944 | 254.3361428 | Incidence | Incidence | 0 | Population (desc) | 3 |
| South Africa | Southern Sub-Saharan Africa | 1241.553831 | 2049.487777 | 807.9339465 | 368.9575772 | 280.9154607 | 158.0609085 | Incidence | Incidence | 0 | Population (desc) | 4 |
| Democratic Republic of the Congo | Central Sub-Saharan Africa | 619.0070889 | 1475.997941 | 856.9908518 | 347.4466034 | 62.65132274 | 446.8929257 | Incidence | Incidence | 0 | Population (desc) | 5 |
| United Republic of Tanzania | Eastern Sub-Saharan Africa | 718.41496 | 1121.406422 | 402.991462 | 342.3322127 | 55.70398607 | 4.955263212 | Incidence | Incidence | 0 | Population (desc) | 6 |
| Mali | Western Sub-Saharan Africa | 452.0920815 | 872.488061 | 420.3959795 | 262.8005522 | 12.34793988 | 145.2474874 | Incidence | Incidence | -5.68434E-14 | Population (desc) | 7 |
| Malawi | Eastern Sub-Saharan Africa | 448.2878939 | 968.4864717 | 520.1985778 | 237.9703765 | 47.71512614 | 234.5130751 | Incidence | Incidence | -1.13687E-13 | Population (desc) | 8 |
| Algeria | North Africa | 717.603892 | 1505.02003 | 787.4161377 | 215.8868439 | 274.84709 | 296.6822037 | Incidence | Incidence | 0 | Population (desc) | 9 |
| Sudan | North Africa | 631.7564066 | 1144.568309 | 512.8119029 | 178.6276636 | 169.982512 | 164.2017273 | Incidence | Incidence | 0 | Population (desc) | 10 |
| Cameroon | Central Sub-Saharan Africa | 276.9747758 | 566.3580805 | 289.3833047 | 171.6778082 | 12.42378634 | 105.2817102 | Incidence | Incidence | 0 | Population (desc) | 11 |
| Uganda | Eastern Sub-Saharan Africa | 365.6067326 | 714.4561944 | 348.8494619 | 170.9025127 | 61.84866931 | 116.0982798 | Incidence | Incidence | 0 | Population (desc) | 12 |
| Madagascar | Eastern Sub-Saharan Africa | 323.9550133 | 649.4611704 | 325.5061571 | 165.6526925 | 29.32843494 | 130.5250297 | Incidence | Incidence | 0 | Population (desc) | 13 |
| Ghana | Western Sub-Saharan Africa | 395.7505041 | 870.5992387 | 474.8487346 | 163.4481674 | 63.87680096 | 247.5237663 | Incidence | Incidence | 5.68434E-14 | Population (desc) | 14 |
| Angola | Central Sub-Saharan Africa | 223.5026224 | 464.9090857 | 241.4064632 | 150.9616646 | 31.87298424 | 58.57181437 | Incidence | Incidence | -2.84217E-14 | Population (desc) | 15 |
| Zambia | Eastern Sub-Saharan Africa | 201.6870828 | 452.2517401 | 250.5646573 | 140.857193 | 16.62221819 | 93.08524609 | Incidence | Incidence | 0 | Population (desc) | 16 |
| Côte d'Ivoire | Western Sub-Saharan Africa | 150.7762616 | 416.8739394 | 266.0976778 | 124.5747583 | 5.414980679 | 136.1079388 | Incidence | Incidence | 0 | Population (desc) | 17 |
| Mozambique | Eastern Sub-Saharan Africa | 268.1066567 | 402.9028683 | 134.7962116 | 120.7949153 | -13.11218603 | 27.11348231 | Incidence | Incidence | 0 | Population (desc) | 18 |
| Tunisia | North Africa | 829.9400543 | 1531.092221 | 701.1521665 | 115.5728304 | 348.9224228 | 236.6569133 | Incidence | Incidence | 0 | Population (desc) | 19 |
| Rwanda | Eastern Sub-Saharan Africa | 182.8867416 | 449.5147385 | 266.6279969 | 84.69191069 | 77.47852202 | 104.4575642 | Incidence | Incidence | 0 | Population (desc) | 20 |
| Niger | Western Sub-Saharan Africa | 118.8153946 | 230.1798049 | 111.3644103 | 81.83716626 | 1.984076089 | 27.5431679 | Incidence | Incidence | -1.42109E-14 | Population (desc) | 21 |
| Kenya | Eastern Sub-Saharan Africa | 237.48015 | 466.3540824 | 228.8739325 | 81.41782665 | 84.30224443 | 63.15386137 | Incidence | Incidence | 0 | Population (desc) | 22 |
| Libya | North Africa | 382.31982 | 593.796733 | 211.476913 | 81.40002924 | 162.866347 | -32.78946329 | Incidence | Incidence | 0 | Population (desc) | 23 |
| Burkina Faso | Western Sub-Saharan Africa | 155.506192 | 272.3669682 | 116.8607762 | 75.60371026 | 5.709710735 | 35.54735521 | Incidence | Incidence | 0 | Population (desc) | 24 |
| Chad | Central Sub-Saharan Africa | 111.6779669 | 199.5444284 | 87.86646153 | 74.16993224 | -17.48423476 | 31.18076405 | Incidence | Incidence | 0 | Population (desc) | 25 |
| Morocco | North Africa | 471.0505405 | 1095.244829 | 624.1942881 | 70.01707424 | 276.7010653 | 277.4761486 | Incidence | Incidence | -1.13687E-13 | Population (desc) | 26 |
| Senegal | Western Sub-Saharan Africa | 123.2812039 | 239.6781262 | 116.3969223 | 69.45142814 | 1.211692835 | 45.73380133 | Incidence | Incidence | 0 | Population (desc) | 27 |
| Zimbabwe | Southern Sub-Saharan Africa | 283.8878896 | 410.8919649 | 127.0040753 | 62.46317884 | 62.56372893 | 1.9771675 | Incidence | Incidence | 0 | Population (desc) | 28 |
| Burundi | Eastern Sub-Saharan Africa | 93.63631324 | 196.0972405 | 102.4609273 | 58.85155319 | 4.563255958 | 39.04611814 | Incidence | Incidence | 1.42109E-14 | Population (desc) | 29 |
| Guinea | Western Sub-Saharan Africa | 104.0115759 | 165.4458229 | 61.43424698 | 46.32725862 | -18.53988635 | 33.64687471 | Incidence | Incidence | 0 | Population (desc) | 30 |
| Somalia | Eastern Sub-Saharan Africa | 65.20927724 | 130.772374 | 65.56309677 | 40.90003982 | 3.76154567 | 20.90151128 | Incidence | Incidence | 0 | Population (desc) | 31 |
| Congo | Central Sub-Saharan Africa | 53.74178904 | 111.6579722 | 57.91618316 | 28.38697991 | 1.667738299 | 27.86146495 | Incidence | Incidence | -7.10543E-15 | Population (desc) | 32 |
| Liberia | Western Sub-Saharan Africa | 52.84883346 | 102.9044594 | 50.0556259 | 26.96741359 | 5.343403615 | 17.7448087 | Incidence | Incidence | 0 | Population (desc) | 33 |
| Sierra Leone | Western Sub-Saharan Africa | 83.62864391 | 139.2027744 | 55.57413045 | 26.64922273 | -8.527665321 | 37.45257305 | Incidence | Incidence | 0 | Population (desc) | 34 |
| Benin | Western Sub-Saharan Africa | 47.05118146 | 83.67818684 | 36.62700537 | 25.8761262 | 4.32378786 | 6.427091315 | Incidence | Incidence | 0 | Population (desc) | 35 |
| Togo | Western Sub-Saharan Africa | 55.90637987 | 126.4710203 | 70.56464039 | 24.96842551 | 19.38258348 | 26.2136314 | Incidence | Incidence | 0 | Population (desc) | 36 |
| Eritrea | Eastern Sub-Saharan Africa | 63.92276195 | 131.453102 | 67.53034006 | 23.57046225 | 11.21897664 | 32.74090116 | Incidence | Incidence | 0 | Population (desc) | 37 |
| Mauritania | Western Sub-Saharan Africa | 51.39508696 | 97.35518072 | 45.96009375 | 22.11129513 | 5.481238367 | 18.36756026 | Incidence | Incidence | 0 | Population (desc) | 38 |
| Central African Republic | Central Sub-Saharan Africa | 28.61050162 | 53.30204079 | 24.69153917 | 12.14605707 | 2.396578433 | 10.14890366 | Incidence | Incidence | 3.55271E-15 | Population (desc) | 39 |
| Equatorial Guinea | Central Sub-Saharan Africa | 16.25149499 | 36.67667409 | 20.42517911 | 11.39915094 | 0.902493832 | 8.123534332 | Incidence | Incidence | 0 | Population (desc) | 40 |
| Gabon | Central Sub-Saharan Africa | 28.15469381 | 57.96037753 | 29.80568372 | 10.00543098 | 4.07969374 | 15.720559 | Incidence | Incidence | -3.55271E-15 | Population (desc) | 41 |
| South Sudan | Eastern Sub-Saharan Africa | 129.3486379 | 198.3292364 | 68.98059856 | 9.538804703 | 36.10575438 | 23.33603948 | Incidence | Incidence | 0 | Population (desc) | 42 |
| Namibia | Southern Sub-Saharan Africa | 17.70440285 | 33.03275073 | 15.32834788 | 8.414433054 | 1.930204778 | 4.983710051 | Incidence | Incidence | 0 | Population (desc) | 43 |
| Djibouti | Eastern Sub-Saharan Africa | 11.0692782 | 28.61447115 | 17.54519294 | 7.817567031 | 3.88739226 | 5.840233652 | Incidence | Incidence | 0 | Population (desc) | 44 |
| Guinea-Bissau | Western Sub-Saharan Africa | 11.87648763 | 22.53876942 | 10.6622818 | 5.264156748 | 0.259410561 | 5.13871449 | Incidence | Incidence | 0 | Population (desc) | 45 |
| Gambia | Western Sub-Saharan Africa | 6.675622025 | 13.55162437 | 6.876002348 | 3.336254125 | 0.323289649 | 3.216458574 | Incidence | Incidence | 8.88178E-16 | Population (desc) | 46 |
| Comoros | Eastern Sub-Saharan Africa | 5.780427965 | 9.50207455 | 3.721646585 | 2.064926462 | 0.818329906 | 0.838390217 | Incidence | Incidence | 0 | Population (desc) | 47 |
| Sao Tome and Principe | Central Sub-Saharan Africa | 6.132713872 | 11.02063118 | 4.887917308 | 1.862372146 | 1.226636111 | 1.798909051 | Incidence | Incidence | 0 | Population (desc) | 48 |
| Botswana | Southern Sub-Saharan Africa | 6.202745607 | 14.80455431 | 8.601808704 | 1.826308112 | 2.248030224 | 4.527470368 | Incidence | Incidence | 0 | Population (desc) | 49 |
| Seychelles | Eastern Sub-Saharan Africa | 7.138248913 | 10.33984684 | 3.201597929 | 1.031709925 | 2.182325008 | -0.012437003 | Incidence | Incidence | -4.44089E-16 | Population (desc) | 50 |
| Lesotho | Southern Sub-Saharan Africa | 15.92881369 | 26.95036111 | 11.02154742 | 0.800833023 | 3.141576313 | 7.07913808 | Incidence | Incidence | 0 | Population (desc) | 51 |
| Eswatini | Southern Sub-Saharan Africa | 7.311386974 | 9.708305634 | 2.39691866 | 0.734342191 | 1.965613019 | -0.30303655 | Incidence | Incidence | 0 | Population (desc) | 52 |
| Mauritius | Eastern Sub-Saharan Africa | 60.19161675 | 84.02221324 | 23.83059649 | 0.011342292 | 25.66054451 | -1.841290313 | Incidence | Incidence | 3.55271E-15 | Population (desc) | 53 |
| Cabo Verde | Western Sub-Saharan Africa | 10.15281093 | 15.79540173 | 5.642590794 | -0.488964256 | 3.064073968 | 3.067481082 | Incidence | Incidence | 0 | Population (desc) | 54 |
| Egypt | North Africa | 18131.70225 | 30309.92238 | 12178.22012 | 6180.145865 | 4120.540027 | 1877.534233 | Prevalence | Prevalence | 0 | Total change (desc) | 1 |
| Ethiopia | Eastern Sub-Saharan Africa | 4091.952611 | 11057.83655 | 6965.883943 | 2035.577998 | 923.5517195 | 4006.754226 | Prevalence | Prevalence | 0 | Total change (desc) | 2 |
| Tunisia | North Africa | 4064.6822 | 10329.24983 | 6264.567629 | 688.3961635 | 2069.743407 | 3506.428059 | Prevalence | Prevalence | -9.09495E-13 | Total change (desc) | 3 |
| South Africa | Southern Sub-Saharan Africa | 5672.50326 | 10745.56173 | 5073.058468 | 1820.063499 | 1390.559065 | 1862.435904 | Prevalence | Prevalence | 0 | Total change (desc) | 4 |
| Algeria | North Africa | 3663.513961 | 7916.531295 | 4253.017335 | 1121.367658 | 1404.822457 | 1726.82722 | Prevalence | Prevalence | 0 | Total change (desc) | 5 |
| Democratic Republic of the Congo | Central Sub-Saharan Africa | 2353.946754 | 6031.710194 | 3677.76344 | 1381.197645 | 287.3001588 | 2009.265637 | Prevalence | Prevalence | -4.54747E-13 | Total change (desc) | 6 |
| Nigeria | Western Sub-Saharan Africa | 3888.312397 | 7328.855907 | 3440.54351 | 2015.253952 | 187.1392311 | 1238.150327 | Prevalence | Prevalence | 0 | Total change (desc) | 7 |
| Sudan | North Africa | 2784.651339 | 5588.186126 | 2803.534786 | 835.1315619 | 770.7173216 | 1197.685903 | Prevalence | Prevalence | 0 | Total change (desc) | 8 |
| Morocco | North Africa | 1935.545323 | 4423.044883 | 2487.49956 | 284.4414412 | 1046.273094 | 1156.785024 | Prevalence | Prevalence | -4.54747E-13 | Total change (desc) | 9 |
| Malawi | Eastern Sub-Saharan Africa | 1825.619053 | 4227.892992 | 2402.273939 | 1009.978084 | 257.7704588 | 1134.525397 | Prevalence | Prevalence | 0 | Total change (desc) | 10 |
| Ghana | Western Sub-Saharan Africa | 1852.103618 | 4126.295632 | 2274.192014 | 770.9047288 | 287.7173396 | 1215.569946 | Prevalence | Prevalence | 0 | Total change (desc) | 11 |
| United Republic of Tanzania | Eastern Sub-Saharan Africa | 2861.443972 | 4781.909614 | 1920.465642 | 1412.430279 | 298.2672656 | 209.7680972 | Prevalence | Prevalence | 0 | Total change (desc) | 12 |
| Uganda | Eastern Sub-Saharan Africa | 1686.779275 | 3603.128312 | 1916.349038 | 829.8991809 | 316.6620252 | 769.7878315 | Prevalence | Prevalence | 0 | Total change (desc) | 13 |
| Mali | Western Sub-Saharan Africa | 1806.806194 | 3544.942488 | 1738.136294 | 1059.959078 | 22.72765054 | 655.4495655 | Prevalence | Prevalence | -2.27374E-13 | Total change (desc) | 14 |
| Madagascar | Eastern Sub-Saharan Africa | 1288.919991 | 2777.959794 | 1489.039803 | 687.0350312 | 176.4754438 | 625.5293284 | Prevalence | Prevalence | -2.27374E-13 | Total change (desc) | 15 |
| Cameroon | Central Sub-Saharan Africa | 1031.898508 | 2380.417958 | 1348.51945 | 685.3759414 | 90.84310287 | 572.3004056 | Prevalence | Prevalence | 0 | Total change (desc) | 16 |
| Côte d'Ivoire | Western Sub-Saharan Africa | 632.9505346 | 1852.066479 | 1219.115944 | 541.57232 | 45.01838744 | 632.5252369 | Prevalence | Prevalence | 0 | Total change (desc) | 17 |
| Rwanda | Eastern Sub-Saharan Africa | 758.7153596 | 1962.007612 | 1203.292252 | 362.6359195 | 310.1294152 | 530.5269176 | Prevalence | Prevalence | 0 | Total change (desc) | 18 |
| Zambia | Eastern Sub-Saharan Africa | 886.47697 | 2069.07891 | 1182.60194 | 633.7106931 | 96.38182021 | 452.5094265 | Prevalence | Prevalence | 0 | Total change (desc) | 19 |
| Angola | Central Sub-Saharan Africa | 874.0552735 | 1953.776216 | 1079.720943 | 614.6768403 | 122.3581391 | 342.6859633 | Prevalence | Prevalence | 0 | Total change (desc) | 20 |
| Kenya | Eastern Sub-Saharan Africa | 1000.90474 | 2064.755607 | 1063.850867 | 352.8344456 | 339.4823379 | 371.534084 | Prevalence | Prevalence | -2.27374E-13 | Total change (desc) | 21 |
| Libya | North Africa | 2145.164159 | 3167.384262 | 1022.220104 | 445.7548247 | 938.1306753 | -361.6653964 | Prevalence | Prevalence | 0 | Total change (desc) | 22 |
| Mozambique | Eastern Sub-Saharan Africa | 1053.88574 | 1831.626069 | 777.7403286 | 512.7170748 | -26.78749106 | 291.8107449 | Prevalence | Prevalence | 0 | Total change (desc) | 23 |
| Zimbabwe | Southern Sub-Saharan Africa | 1257.029785 | 1821.482165 | 564.4523797 | 276.8288687 | 269.2035122 | 18.41999878 | Prevalence | Prevalence | 0 | Total change (desc) | 24 |
| Senegal | Western Sub-Saharan Africa | 498.1639478 | 1003.177589 | 505.0136409 | 286.1965229 | 12.16114464 | 206.6559733 | Prevalence | Prevalence | 0 | Total change (desc) | 25 |
| Burundi | Eastern Sub-Saharan Africa | 393.733043 | 885.008416 | 491.2753729 | 257.5393261 | 37.88227645 | 195.8537703 | Prevalence | Prevalence | 0 | Total change (desc) | 26 |
| Niger | Western Sub-Saharan Africa | 448.7387461 | 859.2174514 | 410.4787053 | 307.2700666 | -9.547231236 | 112.7558699 | Prevalence | Prevalence | 5.68434E-14 | Total change (desc) | 27 |
| Burkina Faso | Western Sub-Saharan Africa | 572.3023123 | 977.7519005 | 405.4495881 | 274.5266986 | 25.53619403 | 105.3866955 | Prevalence | Prevalence | -5.68434E-14 | Total change (desc) | 28 |
| Chad | Central Sub-Saharan Africa | 392.3556224 | 719.8466153 | 327.4909929 | 263.9581837 | -41.86648581 | 105.399295 | Prevalence | Prevalence | 0 | Total change (desc) | 29 |
| Eritrea | Eastern Sub-Saharan Africa | 285.615959 | 598.2009953 | 312.5850364 | 106.4974008 | 44.223946 | 161.8636896 | Prevalence | Prevalence | 0 | Total change (desc) | 30 |
| Togo | Western Sub-Saharan Africa | 235.8029935 | 533.6127193 | 297.8097257 | 105.2980104 | 71.51150447 | 121.0002108 | Prevalence | Prevalence | 0 | Total change (desc) | 31 |
| Guinea | Western Sub-Saharan Africa | 362.9310116 | 631.2875236 | 268.356512 | 169.2480621 | -35.76985642 | 134.8783063 | Prevalence | Prevalence | 0 | Total change (desc) | 32 |
| Somalia | Eastern Sub-Saharan Africa | 291.3934488 | 556.1240344 | 264.7305856 | 178.0601312 | -3.115721942 | 89.78617628 | Prevalence | Prevalence | 0 | Total change (desc) | 33 |
| South Sudan | Eastern Sub-Saharan Africa | 583.929808 | 842.9386523 | 259.0088443 | 41.66732877 | 150.0404775 | 67.30103804 | Prevalence | Prevalence | 0 | Total change (desc) | 34 |
| Sierra Leone | Western Sub-Saharan Africa | 335.6482897 | 591.3956481 | 255.7473584 | 110.3064008 | -14.64384089 | 160.0847985 | Prevalence | Prevalence | 0 | Total change (desc) | 35 |
| Liberia | Western Sub-Saharan Africa | 205.7888975 | 455.9559469 | 250.1670494 | 113.0686668 | 45.02984492 | 92.0685376 | Prevalence | Prevalence | 0 | Total change (desc) | 36 |
| Congo | Central Sub-Saharan Africa | 205.9141105 | 416.5253702 | 210.6112597 | 106.987008 | 13.09212542 | 90.53212623 | Prevalence | Prevalence | -2.84217E-14 | Total change (desc) | 37 |
| Mauritania | Western Sub-Saharan Africa | 184.5223599 | 382.2335469 | 197.711187 | 83.59914391 | 21.81229564 | 92.29974741 | Prevalence | Prevalence | 0 | Total change (desc) | 38 |
| Benin | Western Sub-Saharan Africa | 182.6894548 | 351.3191798 | 168.629725 | 104.8020964 | 21.43897959 | 42.38864895 | Prevalence | Prevalence | 0 | Total change (desc) | 39 |
| Gabon | Central Sub-Saharan Africa | 109.5275381 | 240.1102564 | 130.5827183 | 40.40025855 | 19.17846835 | 71.00399141 | Prevalence | Prevalence | 0 | Total change (desc) | 40 |
| Mauritius | Eastern Sub-Saharan Africa | 332.4755473 | 459.0004704 | 126.5249231 | 0.062069082 | 122.5300676 | 3.93278644 | Prevalence | Prevalence | 0 | Total change (desc) | 41 |
| Central African Republic | Central Sub-Saharan Africa | 103.2135685 | 200.6864995 | 97.47293107 | 44.91872648 | 8.242453424 | 44.31175116 | Prevalence | Prevalence | 0 | Total change (desc) | 42 |
| Djibouti | Eastern Sub-Saharan Africa | 52.45486736 | 140.5183404 | 88.06347307 | 37.8502081 | 16.31675908 | 33.89650589 | Prevalence | Prevalence | 0 | Total change (desc) | 43 |
| Namibia | Southern Sub-Saharan Africa | 88.5113806 | 166.5466388 | 78.03525822 | 42.28634765 | 12.62427163 | 23.12463893 | Prevalence | Prevalence | 0 | Total change (desc) | 44 |
| Equatorial Guinea | Central Sub-Saharan Africa | 61.85919044 | 136.954158 | 75.09496758 | 42.94538097 | 5.627702061 | 26.52188455 | Prevalence | Prevalence | 0 | Total change (desc) | 45 |
| Guinea-Bissau | Western Sub-Saharan Africa | 41.08124773 | 86.35486752 | 45.27361979 | 19.30161415 | 3.366160446 | 22.60584519 | Prevalence | Prevalence | -7.10543E-15 | Total change (desc) | 46 |
| Botswana | Southern Sub-Saharan Africa | 19.74827995 | 55.2078097 | 35.45952975 | 6.457536043 | 7.184458661 | 21.81753504 | Prevalence | Prevalence | 0 | Total change (desc) | 47 |
| Gambia | Western Sub-Saharan Africa | 26.41271089 | 54.9472777 | 28.53456681 | 13.38026421 | 1.892926148 | 13.26137646 | Prevalence | Prevalence | 0 | Total change (desc) | 48 |
| Lesotho | Southern Sub-Saharan Africa | 67.34306534 | 93.31469079 | 25.97162544 | 3.018256127 | 10.1723041 | 12.78106522 | Prevalence | Prevalence | 0 | Total change (desc) | 49 |
| Sao Tome and Principe | Central Sub-Saharan Africa | 24.28033251 | 49.91392209 | 25.63358958 | 7.973308543 | 6.950833931 | 10.70944711 | Prevalence | Prevalence | 0 | Total change (desc) | 50 |
| Seychelles | Eastern Sub-Saharan Africa | 35.61893862 | 56.46012052 | 20.8411819 | 5.404980106 | 11.86084651 | 3.575355279 | Prevalence | Prevalence | 0 | Total change (desc) | 51 |
| Cabo Verde | Western Sub-Saharan Africa | 47.52885394 | 66.59513082 | 19.06627687 | -2.157276063 | 14.70016248 | 6.523390459 | Prevalence | Prevalence | 0 | Total change (desc) | 52 |
| Comoros | Eastern Sub-Saharan Africa | 25.11197218 | 39.67402235 | 14.56205017 | 8.781427113 | 3.315886986 | 2.464736075 | Prevalence | Prevalence | -1.77636E-15 | Total change (desc) | 53 |
| Eswatini | Southern Sub-Saharan Africa | 42.08594244 | 52.88713578 | 10.80119334 | 4.110256755 | 10.25408035 | -3.563143763 | Prevalence | Prevalence | 1.77636E-15 | Total change (desc) | 54 |
| Ethiopia | Eastern Sub-Saharan Africa | 4091.952611 | 11057.83655 | 6965.883943 | 2035.577998 | 923.5517195 | 4006.754226 | Prevalence | Prevalence | 0 | Epidemiology (desc) | 1 |
| Tunisia | North Africa | 4064.6822 | 10329.24983 | 6264.567629 | 688.3961635 | 2069.743407 | 3506.428059 | Prevalence | Prevalence | -9.09495E-13 | Epidemiology (desc) | 2 |
| Democratic Republic of the Congo | Central Sub-Saharan Africa | 2353.946754 | 6031.710194 | 3677.76344 | 1381.197645 | 287.3001588 | 2009.265637 | Prevalence | Prevalence | -4.54747E-13 | Epidemiology (desc) | 3 |
| Egypt | North Africa | 18131.70225 | 30309.92238 | 12178.22012 | 6180.145865 | 4120.540027 | 1877.534233 | Prevalence | Prevalence | 0 | Epidemiology (desc) | 4 |
| South Africa | Southern Sub-Saharan Africa | 5672.50326 | 10745.56173 | 5073.058468 | 1820.063499 | 1390.559065 | 1862.435904 | Prevalence | Prevalence | 0 | Epidemiology (desc) | 5 |
| Algeria | North Africa | 3663.513961 | 7916.531295 | 4253.017335 | 1121.367658 | 1404.822457 | 1726.82722 | Prevalence | Prevalence | 0 | Epidemiology (desc) | 6 |
| Nigeria | Western Sub-Saharan Africa | 3888.312397 | 7328.855907 | 3440.54351 | 2015.253952 | 187.1392311 | 1238.150327 | Prevalence | Prevalence | 0 | Epidemiology (desc) | 7 |
| Ghana | Western Sub-Saharan Africa | 1852.103618 | 4126.295632 | 2274.192014 | 770.9047288 | 287.7173396 | 1215.569946 | Prevalence | Prevalence | 0 | Epidemiology (desc) | 8 |
| Sudan | North Africa | 2784.651339 | 5588.186126 | 2803.534786 | 835.1315619 | 770.7173216 | 1197.685903 | Prevalence | Prevalence | 0 | Epidemiology (desc) | 9 |
| Morocco | North Africa | 1935.545323 | 4423.044883 | 2487.49956 | 284.4414412 | 1046.273094 | 1156.785024 | Prevalence | Prevalence | -4.54747E-13 | Epidemiology (desc) | 10 |
| Malawi | Eastern Sub-Saharan Africa | 1825.619053 | 4227.892992 | 2402.273939 | 1009.978084 | 257.7704588 | 1134.525397 | Prevalence | Prevalence | 0 | Epidemiology (desc) | 11 |
| Uganda | Eastern Sub-Saharan Africa | 1686.779275 | 3603.128312 | 1916.349038 | 829.8991809 | 316.6620252 | 769.7878315 | Prevalence | Prevalence | 0 | Epidemiology (desc) | 12 |
| Mali | Western Sub-Saharan Africa | 1806.806194 | 3544.942488 | 1738.136294 | 1059.959078 | 22.72765054 | 655.4495655 | Prevalence | Prevalence | -2.27374E-13 | Epidemiology (desc) | 13 |
| Côte d'Ivoire | Western Sub-Saharan Africa | 632.9505346 | 1852.066479 | 1219.115944 | 541.57232 | 45.01838744 | 632.5252369 | Prevalence | Prevalence | 0 | Epidemiology (desc) | 14 |
| Madagascar | Eastern Sub-Saharan Africa | 1288.919991 | 2777.959794 | 1489.039803 | 687.0350312 | 176.4754438 | 625.5293284 | Prevalence | Prevalence | -2.27374E-13 | Epidemiology (desc) | 15 |
| Cameroon | Central Sub-Saharan Africa | 1031.898508 | 2380.417958 | 1348.51945 | 685.3759414 | 90.84310287 | 572.3004056 | Prevalence | Prevalence | 0 | Epidemiology (desc) | 16 |
| Rwanda | Eastern Sub-Saharan Africa | 758.7153596 | 1962.007612 | 1203.292252 | 362.6359195 | 310.1294152 | 530.5269176 | Prevalence | Prevalence | 0 | Epidemiology (desc) | 17 |
| Zambia | Eastern Sub-Saharan Africa | 886.47697 | 2069.07891 | 1182.60194 | 633.7106931 | 96.38182021 | 452.5094265 | Prevalence | Prevalence | 0 | Epidemiology (desc) | 18 |
| Kenya | Eastern Sub-Saharan Africa | 1000.90474 | 2064.755607 | 1063.850867 | 352.8344456 | 339.4823379 | 371.534084 | Prevalence | Prevalence | -2.27374E-13 | Epidemiology (desc) | 19 |
| Angola | Central Sub-Saharan Africa | 874.0552735 | 1953.776216 | 1079.720943 | 614.6768403 | 122.3581391 | 342.6859633 | Prevalence | Prevalence | 0 | Epidemiology (desc) | 20 |
| Mozambique | Eastern Sub-Saharan Africa | 1053.88574 | 1831.626069 | 777.7403286 | 512.7170748 | -26.78749106 | 291.8107449 | Prevalence | Prevalence | 0 | Epidemiology (desc) | 21 |
| United Republic of Tanzania | Eastern Sub-Saharan Africa | 2861.443972 | 4781.909614 | 1920.465642 | 1412.430279 | 298.2672656 | 209.7680972 | Prevalence | Prevalence | 0 | Epidemiology (desc) | 22 |
| Senegal | Western Sub-Saharan Africa | 498.1639478 | 1003.177589 | 505.0136409 | 286.1965229 | 12.16114464 | 206.6559733 | Prevalence | Prevalence | 0 | Epidemiology (desc) | 23 |
| Burundi | Eastern Sub-Saharan Africa | 393.733043 | 885.008416 | 491.2753729 | 257.5393261 | 37.88227645 | 195.8537703 | Prevalence | Prevalence | 0 | Epidemiology (desc) | 24 |
| Eritrea | Eastern Sub-Saharan Africa | 285.615959 | 598.2009953 | 312.5850364 | 106.4974008 | 44.223946 | 161.8636896 | Prevalence | Prevalence | 0 | Epidemiology (desc) | 25 |
| Sierra Leone | Western Sub-Saharan Africa | 335.6482897 | 591.3956481 | 255.7473584 | 110.3064008 | -14.64384089 | 160.0847985 | Prevalence | Prevalence | 0 | Epidemiology (desc) | 26 |
| Guinea | Western Sub-Saharan Africa | 362.9310116 | 631.2875236 | 268.356512 | 169.2480621 | -35.76985642 | 134.8783063 | Prevalence | Prevalence | 0 | Epidemiology (desc) | 27 |
| Togo | Western Sub-Saharan Africa | 235.8029935 | 533.6127193 | 297.8097257 | 105.2980104 | 71.51150447 | 121.0002108 | Prevalence | Prevalence | 0 | Epidemiology (desc) | 28 |
| Niger | Western Sub-Saharan Africa | 448.7387461 | 859.2174514 | 410.4787053 | 307.2700666 | -9.547231236 | 112.7558699 | Prevalence | Prevalence | 5.68434E-14 | Epidemiology (desc) | 29 |
| Chad | Central Sub-Saharan Africa | 392.3556224 | 719.8466153 | 327.4909929 | 263.9581837 | -41.86648581 | 105.399295 | Prevalence | Prevalence | 0 | Epidemiology (desc) | 30 |
| Burkina Faso | Western Sub-Saharan Africa | 572.3023123 | 977.7519005 | 405.4495881 | 274.5266986 | 25.53619403 | 105.3866955 | Prevalence | Prevalence | -5.68434E-14 | Epidemiology (desc) | 31 |
| Mauritania | Western Sub-Saharan Africa | 184.5223599 | 382.2335469 | 197.711187 | 83.59914391 | 21.81229564 | 92.29974741 | Prevalence | Prevalence | 0 | Epidemiology (desc) | 32 |
| Liberia | Western Sub-Saharan Africa | 205.7888975 | 455.9559469 | 250.1670494 | 113.0686668 | 45.02984492 | 92.0685376 | Prevalence | Prevalence | 0 | Epidemiology (desc) | 33 |
| Congo | Central Sub-Saharan Africa | 205.9141105 | 416.5253702 | 210.6112597 | 106.987008 | 13.09212542 | 90.53212623 | Prevalence | Prevalence | -2.84217E-14 | Epidemiology (desc) | 34 |
| Somalia | Eastern Sub-Saharan Africa | 291.3934488 | 556.1240344 | 264.7305856 | 178.0601312 | -3.115721942 | 89.78617628 | Prevalence | Prevalence | 0 | Epidemiology (desc) | 35 |
| Gabon | Central Sub-Saharan Africa | 109.5275381 | 240.1102564 | 130.5827183 | 40.40025855 | 19.17846835 | 71.00399141 | Prevalence | Prevalence | 0 | Epidemiology (desc) | 36 |
| South Sudan | Eastern Sub-Saharan Africa | 583.929808 | 842.9386523 | 259.0088443 | 41.66732877 | 150.0404775 | 67.30103804 | Prevalence | Prevalence | 0 | Epidemiology (desc) | 37 |
| Central African Republic | Central Sub-Saharan Africa | 103.2135685 | 200.6864995 | 97.47293107 | 44.91872648 | 8.242453424 | 44.31175116 | Prevalence | Prevalence | 0 | Epidemiology (desc) | 38 |
| Benin | Western Sub-Saharan Africa | 182.6894548 | 351.3191798 | 168.629725 | 104.8020964 | 21.43897959 | 42.38864895 | Prevalence | Prevalence | 0 | Epidemiology (desc) | 39 |
| Djibouti | Eastern Sub-Saharan Africa | 52.45486736 | 140.5183404 | 88.06347307 | 37.8502081 | 16.31675908 | 33.89650589 | Prevalence | Prevalence | 0 | Epidemiology (desc) | 40 |
| Equatorial Guinea | Central Sub-Saharan Africa | 61.85919044 | 136.954158 | 75.09496758 | 42.94538097 | 5.627702061 | 26.52188455 | Prevalence | Prevalence | 0 | Epidemiology (desc) | 41 |
| Namibia | Southern Sub-Saharan Africa | 88.5113806 | 166.5466388 | 78.03525822 | 42.28634765 | 12.62427163 | 23.12463893 | Prevalence | Prevalence | 0 | Epidemiology (desc) | 42 |
| Guinea-Bissau | Western Sub-Saharan Africa | 41.08124773 | 86.35486752 | 45.27361979 | 19.30161415 | 3.366160446 | 22.60584519 | Prevalence | Prevalence | -7.10543E-15 | Epidemiology (desc) | 43 |
| Botswana | Southern Sub-Saharan Africa | 19.74827995 | 55.2078097 | 35.45952975 | 6.457536043 | 7.184458661 | 21.81753504 | Prevalence | Prevalence | 0 | Epidemiology (desc) | 44 |
| Zimbabwe | Southern Sub-Saharan Africa | 1257.029785 | 1821.482165 | 564.4523797 | 276.8288687 | 269.2035122 | 18.41999878 | Prevalence | Prevalence | 0 | Epidemiology (desc) | 45 |
| Gambia | Western Sub-Saharan Africa | 26.41271089 | 54.9472777 | 28.53456681 | 13.38026421 | 1.892926148 | 13.26137646 | Prevalence | Prevalence | 0 | Epidemiology (desc) | 46 |
| Lesotho | Southern Sub-Saharan Africa | 67.34306534 | 93.31469079 | 25.97162544 | 3.018256127 | 10.1723041 | 12.78106522 | Prevalence | Prevalence | 0 | Epidemiology (desc) | 47 |
| Sao Tome and Principe | Central Sub-Saharan Africa | 24.28033251 | 49.91392209 | 25.63358958 | 7.973308543 | 6.950833931 | 10.70944711 | Prevalence | Prevalence | 0 | Epidemiology (desc) | 48 |
| Cabo Verde | Western Sub-Saharan Africa | 47.52885394 | 66.59513082 | 19.06627687 | -2.157276063 | 14.70016248 | 6.523390459 | Prevalence | Prevalence | 0 | Epidemiology (desc) | 49 |
| Mauritius | Eastern Sub-Saharan Africa | 332.4755473 | 459.0004704 | 126.5249231 | 0.062069082 | 122.5300676 | 3.93278644 | Prevalence | Prevalence | 0 | Epidemiology (desc) | 50 |
| Seychelles | Eastern Sub-Saharan Africa | 35.61893862 | 56.46012052 | 20.8411819 | 5.404980106 | 11.86084651 | 3.575355279 | Prevalence | Prevalence | 0 | Epidemiology (desc) | 51 |
| Comoros | Eastern Sub-Saharan Africa | 25.11197218 | 39.67402235 | 14.56205017 | 8.781427113 | 3.315886986 | 2.464736075 | Prevalence | Prevalence | -1.77636E-15 | Epidemiology (desc) | 52 |
| Eswatini | Southern Sub-Saharan Africa | 42.08594244 | 52.88713578 | 10.80119334 | 4.110256755 | 10.25408035 | -3.563143763 | Prevalence | Prevalence | 1.77636E-15 | Epidemiology (desc) | 53 |
| Libya | North Africa | 2145.164159 | 3167.384262 | 1022.220104 | 445.7548247 | 938.1306753 | -361.6653964 | Prevalence | Prevalence | 0 | Epidemiology (desc) | 54 |
| Egypt | North Africa | 18131.70225 | 30309.92238 | 12178.22012 | 6180.145865 | 4120.540027 | 1877.534233 | Prevalence | Prevalence | 0 | Aging/Structure (desc) | 1 |
| Tunisia | North Africa | 4064.6822 | 10329.24983 | 6264.567629 | 688.3961635 | 2069.743407 | 3506.428059 | Prevalence | Prevalence | -9.09495E-13 | Aging/Structure (desc) | 2 |
| Algeria | North Africa | 3663.513961 | 7916.531295 | 4253.017335 | 1121.367658 | 1404.822457 | 1726.82722 | Prevalence | Prevalence | 0 | Aging/Structure (desc) | 3 |
| South Africa | Southern Sub-Saharan Africa | 5672.50326 | 10745.56173 | 5073.058468 | 1820.063499 | 1390.559065 | 1862.435904 | Prevalence | Prevalence | 0 | Aging/Structure (desc) | 4 |
| Morocco | North Africa | 1935.545323 | 4423.044883 | 2487.49956 | 284.4414412 | 1046.273094 | 1156.785024 | Prevalence | Prevalence | -4.54747E-13 | Aging/Structure (desc) | 5 |
| Libya | North Africa | 2145.164159 | 3167.384262 | 1022.220104 | 445.7548247 | 938.1306753 | -361.6653964 | Prevalence | Prevalence | 0 | Aging/Structure (desc) | 6 |
| Ethiopia | Eastern Sub-Saharan Africa | 4091.952611 | 11057.83655 | 6965.883943 | 2035.577998 | 923.5517195 | 4006.754226 | Prevalence | Prevalence | 0 | Aging/Structure (desc) | 7 |
| Sudan | North Africa | 2784.651339 | 5588.186126 | 2803.534786 | 835.1315619 | 770.7173216 | 1197.685903 | Prevalence | Prevalence | 0 | Aging/Structure (desc) | 8 |
| Kenya | Eastern Sub-Saharan Africa | 1000.90474 | 2064.755607 | 1063.850867 | 352.8344456 | 339.4823379 | 371.534084 | Prevalence | Prevalence | -2.27374E-13 | Aging/Structure (desc) | 9 |
| Uganda | Eastern Sub-Saharan Africa | 1686.779275 | 3603.128312 | 1916.349038 | 829.8991809 | 316.6620252 | 769.7878315 | Prevalence | Prevalence | 0 | Aging/Structure (desc) | 10 |
| Rwanda | Eastern Sub-Saharan Africa | 758.7153596 | 1962.007612 | 1203.292252 | 362.6359195 | 310.1294152 | 530.5269176 | Prevalence | Prevalence | 0 | Aging/Structure (desc) | 11 |
| United Republic of Tanzania | Eastern Sub-Saharan Africa | 2861.443972 | 4781.909614 | 1920.465642 | 1412.430279 | 298.2672656 | 209.7680972 | Prevalence | Prevalence | 0 | Aging/Structure (desc) | 12 |
| Ghana | Western Sub-Saharan Africa | 1852.103618 | 4126.295632 | 2274.192014 | 770.9047288 | 287.7173396 | 1215.569946 | Prevalence | Prevalence | 0 | Aging/Structure (desc) | 13 |
| Democratic Republic of the Congo | Central Sub-Saharan Africa | 2353.946754 | 6031.710194 | 3677.76344 | 1381.197645 | 287.3001588 | 2009.265637 | Prevalence | Prevalence | -4.54747E-13 | Aging/Structure (desc) | 14 |
| Zimbabwe | Southern Sub-Saharan Africa | 1257.029785 | 1821.482165 | 564.4523797 | 276.8288687 | 269.2035122 | 18.41999878 | Prevalence | Prevalence | 0 | Aging/Structure (desc) | 15 |
| Malawi | Eastern Sub-Saharan Africa | 1825.619053 | 4227.892992 | 2402.273939 | 1009.978084 | 257.7704588 | 1134.525397 | Prevalence | Prevalence | 0 | Aging/Structure (desc) | 16 |
| Nigeria | Western Sub-Saharan Africa | 3888.312397 | 7328.855907 | 3440.54351 | 2015.253952 | 187.1392311 | 1238.150327 | Prevalence | Prevalence | 0 | Aging/Structure (desc) | 17 |
| Madagascar | Eastern Sub-Saharan Africa | 1288.919991 | 2777.959794 | 1489.039803 | 687.0350312 | 176.4754438 | 625.5293284 | Prevalence | Prevalence | -2.27374E-13 | Aging/Structure (desc) | 18 |
| South Sudan | Eastern Sub-Saharan Africa | 583.929808 | 842.9386523 | 259.0088443 | 41.66732877 | 150.0404775 | 67.30103804 | Prevalence | Prevalence | 0 | Aging/Structure (desc) | 19 |
| Mauritius | Eastern Sub-Saharan Africa | 332.4755473 | 459.0004704 | 126.5249231 | 0.062069082 | 122.5300676 | 3.93278644 | Prevalence | Prevalence | 0 | Aging/Structure (desc) | 20 |
| Angola | Central Sub-Saharan Africa | 874.0552735 | 1953.776216 | 1079.720943 | 614.6768403 | 122.3581391 | 342.6859633 | Prevalence | Prevalence | 0 | Aging/Structure (desc) | 21 |
| Zambia | Eastern Sub-Saharan Africa | 886.47697 | 2069.07891 | 1182.60194 | 633.7106931 | 96.38182021 | 452.5094265 | Prevalence | Prevalence | 0 | Aging/Structure (desc) | 22 |
| Cameroon | Central Sub-Saharan Africa | 1031.898508 | 2380.417958 | 1348.51945 | 685.3759414 | 90.84310287 | 572.3004056 | Prevalence | Prevalence | 0 | Aging/Structure (desc) | 23 |
| Togo | Western Sub-Saharan Africa | 235.8029935 | 533.6127193 | 297.8097257 | 105.2980104 | 71.51150447 | 121.0002108 | Prevalence | Prevalence | 0 | Aging/Structure (desc) | 24 |
| Liberia | Western Sub-Saharan Africa | 205.7888975 | 455.9559469 | 250.1670494 | 113.0686668 | 45.02984492 | 92.0685376 | Prevalence | Prevalence | 0 | Aging/Structure (desc) | 25 |
| Côte d'Ivoire | Western Sub-Saharan Africa | 632.9505346 | 1852.066479 | 1219.115944 | 541.57232 | 45.01838744 | 632.5252369 | Prevalence | Prevalence | 0 | Aging/Structure (desc) | 26 |
| Eritrea | Eastern Sub-Saharan Africa | 285.615959 | 598.2009953 | 312.5850364 | 106.4974008 | 44.223946 | 161.8636896 | Prevalence | Prevalence | 0 | Aging/Structure (desc) | 27 |
| Burundi | Eastern Sub-Saharan Africa | 393.733043 | 885.008416 | 491.2753729 | 257.5393261 | 37.88227645 | 195.8537703 | Prevalence | Prevalence | 0 | Aging/Structure (desc) | 28 |
| Burkina Faso | Western Sub-Saharan Africa | 572.3023123 | 977.7519005 | 405.4495881 | 274.5266986 | 25.53619403 | 105.3866955 | Prevalence | Prevalence | -5.68434E-14 | Aging/Structure (desc) | 29 |
| Mali | Western Sub-Saharan Africa | 1806.806194 | 3544.942488 | 1738.136294 | 1059.959078 | 22.72765054 | 655.4495655 | Prevalence | Prevalence | -2.27374E-13 | Aging/Structure (desc) | 30 |
| Mauritania | Western Sub-Saharan Africa | 184.5223599 | 382.2335469 | 197.711187 | 83.59914391 | 21.81229564 | 92.29974741 | Prevalence | Prevalence | 0 | Aging/Structure (desc) | 31 |
| Benin | Western Sub-Saharan Africa | 182.6894548 | 351.3191798 | 168.629725 | 104.8020964 | 21.43897959 | 42.38864895 | Prevalence | Prevalence | 0 | Aging/Structure (desc) | 32 |
| Gabon | Central Sub-Saharan Africa | 109.5275381 | 240.1102564 | 130.5827183 | 40.40025855 | 19.17846835 | 71.00399141 | Prevalence | Prevalence | 0 | Aging/Structure (desc) | 33 |
| Djibouti | Eastern Sub-Saharan Africa | 52.45486736 | 140.5183404 | 88.06347307 | 37.8502081 | 16.31675908 | 33.89650589 | Prevalence | Prevalence | 0 | Aging/Structure (desc) | 34 |
| Cabo Verde | Western Sub-Saharan Africa | 47.52885394 | 66.59513082 | 19.06627687 | -2.157276063 | 14.70016248 | 6.523390459 | Prevalence | Prevalence | 0 | Aging/Structure (desc) | 35 |
| Congo | Central Sub-Saharan Africa | 205.9141105 | 416.5253702 | 210.6112597 | 106.987008 | 13.09212542 | 90.53212623 | Prevalence | Prevalence | -2.84217E-14 | Aging/Structure (desc) | 36 |
| Namibia | Southern Sub-Saharan Africa | 88.5113806 | 166.5466388 | 78.03525822 | 42.28634765 | 12.62427163 | 23.12463893 | Prevalence | Prevalence | 0 | Aging/Structure (desc) | 37 |
| Senegal | Western Sub-Saharan Africa | 498.1639478 | 1003.177589 | 505.0136409 | 286.1965229 | 12.16114464 | 206.6559733 | Prevalence | Prevalence | 0 | Aging/Structure (desc) | 38 |
| Seychelles | Eastern Sub-Saharan Africa | 35.61893862 | 56.46012052 | 20.8411819 | 5.404980106 | 11.86084651 | 3.575355279 | Prevalence | Prevalence | 0 | Aging/Structure (desc) | 39 |
| Eswatini | Southern Sub-Saharan Africa | 42.08594244 | 52.88713578 | 10.80119334 | 4.110256755 | 10.25408035 | -3.563143763 | Prevalence | Prevalence | 1.77636E-15 | Aging/Structure (desc) | 40 |
| Lesotho | Southern Sub-Saharan Africa | 67.34306534 | 93.31469079 | 25.97162544 | 3.018256127 | 10.1723041 | 12.78106522 | Prevalence | Prevalence | 0 | Aging/Structure (desc) | 41 |
| Central African Republic | Central Sub-Saharan Africa | 103.2135685 | 200.6864995 | 97.47293107 | 44.91872648 | 8.242453424 | 44.31175116 | Prevalence | Prevalence | 0 | Aging/Structure (desc) | 42 |
| Botswana | Southern Sub-Saharan Africa | 19.74827995 | 55.2078097 | 35.45952975 | 6.457536043 | 7.184458661 | 21.81753504 | Prevalence | Prevalence | 0 | Aging/Structure (desc) | 43 |
| Sao Tome and Principe | Central Sub-Saharan Africa | 24.28033251 | 49.91392209 | 25.63358958 | 7.973308543 | 6.950833931 | 10.70944711 | Prevalence | Prevalence | 0 | Aging/Structure (desc) | 44 |
| Equatorial Guinea | Central Sub-Saharan Africa | 61.85919044 | 136.954158 | 75.09496758 | 42.94538097 | 5.627702061 | 26.52188455 | Prevalence | Prevalence | 0 | Aging/Structure (desc) | 45 |
| Guinea-Bissau | Western Sub-Saharan Africa | 41.08124773 | 86.35486752 | 45.27361979 | 19.30161415 | 3.366160446 | 22.60584519 | Prevalence | Prevalence | -7.10543E-15 | Aging/Structure (desc) | 46 |
| Comoros | Eastern Sub-Saharan Africa | 25.11197218 | 39.67402235 | 14.56205017 | 8.781427113 | 3.315886986 | 2.464736075 | Prevalence | Prevalence | -1.77636E-15 | Aging/Structure (desc) | 47 |
| Gambia | Western Sub-Saharan Africa | 26.41271089 | 54.9472777 | 28.53456681 | 13.38026421 | 1.892926148 | 13.26137646 | Prevalence | Prevalence | 0 | Aging/Structure (desc) | 48 |
| Somalia | Eastern Sub-Saharan Africa | 291.3934488 | 556.1240344 | 264.7305856 | 178.0601312 | -3.115721942 | 89.78617628 | Prevalence | Prevalence | 0 | Aging/Structure (desc) | 49 |
| Niger | Western Sub-Saharan Africa | 448.7387461 | 859.2174514 | 410.4787053 | 307.2700666 | -9.547231236 | 112.7558699 | Prevalence | Prevalence | 5.68434E-14 | Aging/Structure (desc) | 50 |
| Sierra Leone | Western Sub-Saharan Africa | 335.6482897 | 591.3956481 | 255.7473584 | 110.3064008 | -14.64384089 | 160.0847985 | Prevalence | Prevalence | 0 | Aging/Structure (desc) | 51 |
| Mozambique | Eastern Sub-Saharan Africa | 1053.88574 | 1831.626069 | 777.7403286 | 512.7170748 | -26.78749106 | 291.8107449 | Prevalence | Prevalence | 0 | Aging/Structure (desc) | 52 |
| Guinea | Western Sub-Saharan Africa | 362.9310116 | 631.2875236 | 268.356512 | 169.2480621 | -35.76985642 | 134.8783063 | Prevalence | Prevalence | 0 | Aging/Structure (desc) | 53 |
| Chad | Central Sub-Saharan Africa | 392.3556224 | 719.8466153 | 327.4909929 | 263.9581837 | -41.86648581 | 105.399295 | Prevalence | Prevalence | 0 | Aging/Structure (desc) | 54 |
| Egypt | North Africa | 18131.70225 | 30309.92238 | 12178.22012 | 6180.145865 | 4120.540027 | 1877.534233 | Prevalence | Prevalence | 0 | Population (desc) | 1 |
| Ethiopia | Eastern Sub-Saharan Africa | 4091.952611 | 11057.83655 | 6965.883943 | 2035.577998 | 923.5517195 | 4006.754226 | Prevalence | Prevalence | 0 | Population (desc) | 2 |
| Nigeria | Western Sub-Saharan Africa | 3888.312397 | 7328.855907 | 3440.54351 | 2015.253952 | 187.1392311 | 1238.150327 | Prevalence | Prevalence | 0 | Population (desc) | 3 |
| South Africa | Southern Sub-Saharan Africa | 5672.50326 | 10745.56173 | 5073.058468 | 1820.063499 | 1390.559065 | 1862.435904 | Prevalence | Prevalence | 0 | Population (desc) | 4 |
| United Republic of Tanzania | Eastern Sub-Saharan Africa | 2861.443972 | 4781.909614 | 1920.465642 | 1412.430279 | 298.2672656 | 209.7680972 | Prevalence | Prevalence | 0 | Population (desc) | 5 |
| Democratic Republic of the Congo | Central Sub-Saharan Africa | 2353.946754 | 6031.710194 | 3677.76344 | 1381.197645 | 287.3001588 | 2009.265637 | Prevalence | Prevalence | -4.54747E-13 | Population (desc) | 6 |
| Algeria | North Africa | 3663.513961 | 7916.531295 | 4253.017335 | 1121.367658 | 1404.822457 | 1726.82722 | Prevalence | Prevalence | 0 | Population (desc) | 7 |
| Mali | Western Sub-Saharan Africa | 1806.806194 | 3544.942488 | 1738.136294 | 1059.959078 | 22.72765054 | 655.4495655 | Prevalence | Prevalence | -2.27374E-13 | Population (desc) | 8 |
| Malawi | Eastern Sub-Saharan Africa | 1825.619053 | 4227.892992 | 2402.273939 | 1009.978084 | 257.7704588 | 1134.525397 | Prevalence | Prevalence | 0 | Population (desc) | 9 |
| Sudan | North Africa | 2784.651339 | 5588.186126 | 2803.534786 | 835.1315619 | 770.7173216 | 1197.685903 | Prevalence | Prevalence | 0 | Population (desc) | 10 |
| Uganda | Eastern Sub-Saharan Africa | 1686.779275 | 3603.128312 | 1916.349038 | 829.8991809 | 316.6620252 | 769.7878315 | Prevalence | Prevalence | 0 | Population (desc) | 11 |
| Ghana | Western Sub-Saharan Africa | 1852.103618 | 4126.295632 | 2274.192014 | 770.9047288 | 287.7173396 | 1215.569946 | Prevalence | Prevalence | 0 | Population (desc) | 12 |
| Tunisia | North Africa | 4064.6822 | 10329.24983 | 6264.567629 | 688.3961635 | 2069.743407 | 3506.428059 | Prevalence | Prevalence | -9.09495E-13 | Population (desc) | 13 |
| Madagascar | Eastern Sub-Saharan Africa | 1288.919991 | 2777.959794 | 1489.039803 | 687.0350312 | 176.4754438 | 625.5293284 | Prevalence | Prevalence | -2.27374E-13 | Population (desc) | 14 |
| Cameroon | Central Sub-Saharan Africa | 1031.898508 | 2380.417958 | 1348.51945 | 685.3759414 | 90.84310287 | 572.3004056 | Prevalence | Prevalence | 0 | Population (desc) | 15 |
| Zambia | Eastern Sub-Saharan Africa | 886.47697 | 2069.07891 | 1182.60194 | 633.7106931 | 96.38182021 | 452.5094265 | Prevalence | Prevalence | 0 | Population (desc) | 16 |
| Angola | Central Sub-Saharan Africa | 874.0552735 | 1953.776216 | 1079.720943 | 614.6768403 | 122.3581391 | 342.6859633 | Prevalence | Prevalence | 0 | Population (desc) | 17 |
| Côte d'Ivoire | Western Sub-Saharan Africa | 632.9505346 | 1852.066479 | 1219.115944 | 541.57232 | 45.01838744 | 632.5252369 | Prevalence | Prevalence | 0 | Population (desc) | 18 |
| Mozambique | Eastern Sub-Saharan Africa | 1053.88574 | 1831.626069 | 777.7403286 | 512.7170748 | -26.78749106 | 291.8107449 | Prevalence | Prevalence | 0 | Population (desc) | 19 |
| Libya | North Africa | 2145.164159 | 3167.384262 | 1022.220104 | 445.7548247 | 938.1306753 | -361.6653964 | Prevalence | Prevalence | 0 | Population (desc) | 20 |
| Rwanda | Eastern Sub-Saharan Africa | 758.7153596 | 1962.007612 | 1203.292252 | 362.6359195 | 310.1294152 | 530.5269176 | Prevalence | Prevalence | 0 | Population (desc) | 21 |
| Kenya | Eastern Sub-Saharan Africa | 1000.90474 | 2064.755607 | 1063.850867 | 352.8344456 | 339.4823379 | 371.534084 | Prevalence | Prevalence | -2.27374E-13 | Population (desc) | 22 |
| Niger | Western Sub-Saharan Africa | 448.7387461 | 859.2174514 | 410.4787053 | 307.2700666 | -9.547231236 | 112.7558699 | Prevalence | Prevalence | 5.68434E-14 | Population (desc) | 23 |
| Senegal | Western Sub-Saharan Africa | 498.1639478 | 1003.177589 | 505.0136409 | 286.1965229 | 12.16114464 | 206.6559733 | Prevalence | Prevalence | 0 | Population (desc) | 24 |
| Morocco | North Africa | 1935.545323 | 4423.044883 | 2487.49956 | 284.4414412 | 1046.273094 | 1156.785024 | Prevalence | Prevalence | -4.54747E-13 | Population (desc) | 25 |
| Zimbabwe | Southern Sub-Saharan Africa | 1257.029785 | 1821.482165 | 564.4523797 | 276.8288687 | 269.2035122 | 18.41999878 | Prevalence | Prevalence | 0 | Population (desc) | 26 |
| Burkina Faso | Western Sub-Saharan Africa | 572.3023123 | 977.7519005 | 405.4495881 | 274.5266986 | 25.53619403 | 105.3866955 | Prevalence | Prevalence | -5.68434E-14 | Population (desc) | 27 |
| Chad | Central Sub-Saharan Africa | 392.3556224 | 719.8466153 | 327.4909929 | 263.9581837 | -41.86648581 | 105.399295 | Prevalence | Prevalence | 0 | Population (desc) | 28 |
| Burundi | Eastern Sub-Saharan Africa | 393.733043 | 885.008416 | 491.2753729 | 257.5393261 | 37.88227645 | 195.8537703 | Prevalence | Prevalence | 0 | Population (desc) | 29 |
| Somalia | Eastern Sub-Saharan Africa | 291.3934488 | 556.1240344 | 264.7305856 | 178.0601312 | -3.115721942 | 89.78617628 | Prevalence | Prevalence | 0 | Population (desc) | 30 |
| Guinea | Western Sub-Saharan Africa | 362.9310116 | 631.2875236 | 268.356512 | 169.2480621 | -35.76985642 | 134.8783063 | Prevalence | Prevalence | 0 | Population (desc) | 31 |
| Liberia | Western Sub-Saharan Africa | 205.7888975 | 455.9559469 | 250.1670494 | 113.0686668 | 45.02984492 | 92.0685376 | Prevalence | Prevalence | 0 | Population (desc) | 32 |
| Sierra Leone | Western Sub-Saharan Africa | 335.6482897 | 591.3956481 | 255.7473584 | 110.3064008 | -14.64384089 | 160.0847985 | Prevalence | Prevalence | 0 | Population (desc) | 33 |
| Congo | Central Sub-Saharan Africa | 205.9141105 | 416.5253702 | 210.6112597 | 106.987008 | 13.09212542 | 90.53212623 | Prevalence | Prevalence | -2.84217E-14 | Population (desc) | 34 |
| Eritrea | Eastern Sub-Saharan Africa | 285.615959 | 598.2009953 | 312.5850364 | 106.4974008 | 44.223946 | 161.8636896 | Prevalence | Prevalence | 0 | Population (desc) | 35 |
| Togo | Western Sub-Saharan Africa | 235.8029935 | 533.6127193 | 297.8097257 | 105.2980104 | 71.51150447 | 121.0002108 | Prevalence | Prevalence | 0 | Population (desc) | 36 |
| Benin | Western Sub-Saharan Africa | 182.6894548 | 351.3191798 | 168.629725 | 104.8020964 | 21.43897959 | 42.38864895 | Prevalence | Prevalence | 0 | Population (desc) | 37 |
| Mauritania | Western Sub-Saharan Africa | 184.5223599 | 382.2335469 | 197.711187 | 83.59914391 | 21.81229564 | 92.29974741 | Prevalence | Prevalence | 0 | Population (desc) | 38 |
| Central African Republic | Central Sub-Saharan Africa | 103.2135685 | 200.6864995 | 97.47293107 | 44.91872648 | 8.242453424 | 44.31175116 | Prevalence | Prevalence | 0 | Population (desc) | 39 |
| Equatorial Guinea | Central Sub-Saharan Africa | 61.85919044 | 136.954158 | 75.09496758 | 42.94538097 | 5.627702061 | 26.52188455 | Prevalence | Prevalence | 0 | Population (desc) | 40 |
| Namibia | Southern Sub-Saharan Africa | 88.5113806 | 166.5466388 | 78.03525822 | 42.28634765 | 12.62427163 | 23.12463893 | Prevalence | Prevalence | 0 | Population (desc) | 41 |
| South Sudan | Eastern Sub-Saharan Africa | 583.929808 | 842.9386523 | 259.0088443 | 41.66732877 | 150.0404775 | 67.30103804 | Prevalence | Prevalence | 0 | Population (desc) | 42 |
| Gabon | Central Sub-Saharan Africa | 109.5275381 | 240.1102564 | 130.5827183 | 40.40025855 | 19.17846835 | 71.00399141 | Prevalence | Prevalence | 0 | Population (desc) | 43 |
| Djibouti | Eastern Sub-Saharan Africa | 52.45486736 | 140.5183404 | 88.06347307 | 37.8502081 | 16.31675908 | 33.89650589 | Prevalence | Prevalence | 0 | Population (desc) | 44 |
| Guinea-Bissau | Western Sub-Saharan Africa | 41.08124773 | 86.35486752 | 45.27361979 | 19.30161415 | 3.366160446 | 22.60584519 | Prevalence | Prevalence | -7.10543E-15 | Population (desc) | 45 |
| Gambia | Western Sub-Saharan Africa | 26.41271089 | 54.9472777 | 28.53456681 | 13.38026421 | 1.892926148 | 13.26137646 | Prevalence | Prevalence | 0 | Population (desc) | 46 |
| Comoros | Eastern Sub-Saharan Africa | 25.11197218 | 39.67402235 | 14.56205017 | 8.781427113 | 3.315886986 | 2.464736075 | Prevalence | Prevalence | -1.77636E-15 | Population (desc) | 47 |
| Sao Tome and Principe | Central Sub-Saharan Africa | 24.28033251 | 49.91392209 | 25.63358958 | 7.973308543 | 6.950833931 | 10.70944711 | Prevalence | Prevalence | 0 | Population (desc) | 48 |
| Botswana | Southern Sub-Saharan Africa | 19.74827995 | 55.2078097 | 35.45952975 | 6.457536043 | 7.184458661 | 21.81753504 | Prevalence | Prevalence | 0 | Population (desc) | 49 |
| Seychelles | Eastern Sub-Saharan Africa | 35.61893862 | 56.46012052 | 20.8411819 | 5.404980106 | 11.86084651 | 3.575355279 | Prevalence | Prevalence | 0 | Population (desc) | 50 |
| Eswatini | Southern Sub-Saharan Africa | 42.08594244 | 52.88713578 | 10.80119334 | 4.110256755 | 10.25408035 | -3.563143763 | Prevalence | Prevalence | 1.77636E-15 | Population (desc) | 51 |
| Lesotho | Southern Sub-Saharan Africa | 67.34306534 | 93.31469079 | 25.97162544 | 3.018256127 | 10.1723041 | 12.78106522 | Prevalence | Prevalence | 0 | Population (desc) | 52 |
| Mauritius | Eastern Sub-Saharan Africa | 332.4755473 | 459.0004704 | 126.5249231 | 0.062069082 | 122.5300676 | 3.93278644 | Prevalence | Prevalence | 0 | Population (desc) | 53 |
| Cabo Verde | Western Sub-Saharan Africa | 47.52885394 | 66.59513082 | 19.06627687 | -2.157276063 | 14.70016248 | 6.523390459 | Prevalence | Prevalence | 0 | Population (desc) | 54 |
| Egypt | North Africa | 1604.010006 | 2670.431297 | 1066.421291 | 548.0600179 | 478.9910484 | 39.37022428 | Deaths | Deaths | 0 | Total change (desc) | 1 |
| Ethiopia | Eastern Sub-Saharan Africa | 663.9420159 | 1486.747509 | 822.8054935 | 395.6323247 | 32.67336535 | 394.4998034 | Deaths | Deaths | 0 | Total change (desc) | 2 |
| Democratic Republic of the Congo | Central Sub-Saharan Africa | 395.7489603 | 857.4262059 | 461.6772456 | 239.7238718 | 2.982111442 | 218.9712623 | Deaths | Deaths | 0 | Total change (desc) | 3 |
| Nigeria | Western Sub-Saharan Africa | 605.5797419 | 1004.897211 | 399.3174688 | 316.4181903 | -21.6736091 | 104.5728877 | Deaths | Deaths | -5.68434E-14 | Total change (desc) | 4 |
| South Africa | Southern Sub-Saharan Africa | 707.5486592 | 1086.836772 | 379.2881126 | 249.0200167 | 101.1155688 | 29.15252711 | Deaths | Deaths | 0 | Total change (desc) | 5 |
| Algeria | North Africa | 353.1393255 | 662.8779752 | 309.7386497 | 93.11635927 | 138.5780074 | 78.04428309 | Deaths | Deaths | 0 | Total change (desc) | 6 |
| Morocco | North Africa | 275.6103019 | 574.4151945 | 298.8048926 | 74.95482001 | 124.5823253 | 99.26774728 | Deaths | Deaths | 0 | Total change (desc) | 7 |
| Malawi | Eastern Sub-Saharan Africa | 270.6739904 | 522.6856891 | 252.0116986 | 175.7920392 | -21.39162952 | 97.61128897 | Deaths | Deaths | 0 | Total change (desc) | 8 |
| Mali | Western Sub-Saharan Africa | 276.0451347 | 515.7255284 | 239.6803937 | 169.4067023 | 2.100685145 | 68.17300619 | Deaths | Deaths | 0 | Total change (desc) | 9 |
| United Republic of Tanzania | Eastern Sub-Saharan Africa | 455.7458281 | 684.6217131 | 228.875885 | 241.6135377 | -0.541053895 | -12.19659879 | Deaths | Deaths | 2.84217E-14 | Total change (desc) | 10 |
| Tunisia | North Africa | 394.2318743 | 618.7295118 | 224.4976374 | 60.95966928 | 144.4292551 | 19.10871303 | Deaths | Deaths | 0 | Total change (desc) | 11 |
| Ghana | Western Sub-Saharan Africa | 213.767963 | 433.6986218 | 219.9306588 | 102.2572775 | 17.83945789 | 99.83392344 | Deaths | Deaths | 0 | Total change (desc) | 12 |
| Sudan | North Africa | 360.1915736 | 577.4688549 | 217.2772813 | 141.0364511 | 47.49170832 | 28.74912181 | Deaths | Deaths | -2.84217E-14 | Total change (desc) | 13 |
| Madagascar | Eastern Sub-Saharan Africa | 201.5654447 | 367.3474213 | 165.7819766 | 123.479298 | -15.05430979 | 57.35698839 | Deaths | Deaths | 0 | Total change (desc) | 14 |
| Uganda | Eastern Sub-Saharan Africa | 211.421916 | 375.2067242 | 163.7848082 | 118.876367 | 8.390704424 | 36.5177368 | Deaths | Deaths | 0 | Total change (desc) | 15 |
| Cameroon | Central Sub-Saharan Africa | 176.3110917 | 320.1833949 | 143.8723032 | 112.8353849 | -7.194737363 | 38.23165568 | Deaths | Deaths | 0 | Total change (desc) | 16 |
| Côte d'Ivoire | Western Sub-Saharan Africa | 94.59904622 | 229.5066155 | 134.9075693 | 79.74363641 | -6.517443775 | 61.68137667 | Deaths | Deaths | 0 | Total change (desc) | 17 |
| Rwanda | Eastern Sub-Saharan Africa | 111.5905961 | 245.9371291 | 134.346533 | 61.6031415 | 32.52658055 | 40.21681095 | Deaths | Deaths | 0 | Total change (desc) | 18 |
| Angola | Central Sub-Saharan Africa | 139.7620625 | 267.2352695 | 127.473207 | 97.27011338 | 13.07580814 | 17.12728548 | Deaths | Deaths | -1.42109E-14 | Total change (desc) | 19 |
| Zambia | Eastern Sub-Saharan Africa | 115.6226946 | 235.0729277 | 119.4502331 | 87.73099785 | -4.912323413 | 36.63155864 | Deaths | Deaths | 0 | Total change (desc) | 20 |
| Kenya | Eastern Sub-Saharan Africa | 137.6824076 | 251.4715164 | 113.7891088 | 67.86628927 | 27.4873498 | 18.43546974 | Deaths | Deaths | -1.42109E-14 | Total change (desc) | 21 |
| Libya | North Africa | 164.6956096 | 257.3953631 | 92.69975352 | 48.27471783 | 51.97900393 | -7.553968245 | Deaths | Deaths | 0 | Total change (desc) | 22 |
| Mozambique | Eastern Sub-Saharan Africa | 181.6659995 | 264.7393277 | 83.07332821 | 88.61154457 | -20.06846023 | 14.53024386 | Deaths | Deaths | 1.42109E-14 | Total change (desc) | 23 |
| Burkina Faso | Western Sub-Saharan Africa | 104.8475987 | 182.7620764 | 77.91447775 | 56.89495668 | -2.232892468 | 23.25241354 | Deaths | Deaths | 0 | Total change (desc) | 24 |
| Zimbabwe | Southern Sub-Saharan Africa | 173.2875841 | 250.3970836 | 77.10949947 | 45.92833623 | 30.64043003 | 0.540733199 | Deaths | Deaths | 0 | Total change (desc) | 25 |
| Niger | Western Sub-Saharan Africa | 74.87761713 | 143.7562413 | 68.87862419 | 53.67468084 | 0.978238183 | 14.22570517 | Deaths | Deaths | 0 | Total change (desc) | 26 |
| Senegal | Western Sub-Saharan Africa | 80.56879852 | 145.5374269 | 64.96862837 | 47.60969152 | -3.730761452 | 21.0896983 | Deaths | Deaths | 0 | Total change (desc) | 27 |
| Chad | Central Sub-Saharan Africa | 76.25536602 | 129.6464602 | 53.39109416 | 50.00188841 | -14.75444597 | 18.14365172 | Deaths | Deaths | 0 | Total change (desc) | 28 |
| Burundi | Eastern Sub-Saharan Africa | 57.26753013 | 107.4562451 | 50.18871495 | 36.11971184 | -2.462954529 | 16.53195764 | Deaths | Deaths | 0 | Total change (desc) | 29 |
| Somalia | Eastern Sub-Saharan Africa | 42.39147176 | 86.44942616 | 44.0579544 | 27.49409506 | 4.340777598 | 12.22308174 | Deaths | Deaths | 0 | Total change (desc) | 30 |
| South Sudan | Eastern Sub-Saharan Africa | 74.9716973 | 114.3239522 | 39.3522549 | 13.86410528 | 12.91730297 | 12.57084665 | Deaths | Deaths | 0 | Total change (desc) | 31 |
| Togo | Western Sub-Saharan Africa | 34.36761729 | 73.04211907 | 38.67450177 | 18.50813685 | 9.036869574 | 11.12949535 | Deaths | Deaths | 0 | Total change (desc) | 32 |
| Eritrea | Eastern Sub-Saharan Africa | 37.19520095 | 73.21883494 | 36.02363399 | 15.61878346 | 5.391434886 | 15.01341564 | Deaths | Deaths | 0 | Total change (desc) | 33 |
| Guinea | Western Sub-Saharan Africa | 74.15057462 | 109.2091465 | 35.05857189 | 33.89184398 | -18.30175405 | 19.46848196 | Deaths | Deaths | 0 | Total change (desc) | 34 |
| Congo | Central Sub-Saharan Africa | 34.00656067 | 65.42464495 | 31.41808428 | 18.03359684 | -0.689987904 | 14.07447535 | Deaths | Deaths | 0 | Total change (desc) | 35 |
| Sierra Leone | Western Sub-Saharan Africa | 53.4484448 | 80.65559964 | 27.20715484 | 23.24299982 | -14.12728686 | 18.09144188 | Deaths | Deaths | 0 | Total change (desc) | 36 |
| Mauritania | Western Sub-Saharan Africa | 31.91092179 | 55.14717871 | 23.23625692 | 15.29038526 | 0.7861046 | 7.159767062 | Deaths | Deaths | 0 | Total change (desc) | 37 |
| Liberia | Western Sub-Saharan Africa | 33.39817139 | 55.7914315 | 22.3932601 | 21.36484751 | -5.592517492 | 6.620930084 | Deaths | Deaths | 0 | Total change (desc) | 38 |
| Benin | Western Sub-Saharan Africa | 30.02094939 | 49.58943339 | 19.568484 | 18.39233551 | -0.257387797 | 1.433536286 | Deaths | Deaths | 0 | Total change (desc) | 39 |
| Gabon | Central Sub-Saharan Africa | 18.03156219 | 33.7517258 | 15.7201636 | 6.926693072 | 1.350942623 | 7.442527907 | Deaths | Deaths | 0 | Total change (desc) | 40 |
| Central African Republic | Central Sub-Saharan Africa | 19.50191876 | 35.04542584 | 15.54350708 | 7.714027206 | 2.031746745 | 5.797733128 | Deaths | Deaths | 0 | Total change (desc) | 41 |
| Mauritius | Eastern Sub-Saharan Africa | 26.21870696 | 37.0870244 | 10.86831744 | 2.180648445 | 11.09044705 | -2.402778055 | Deaths | Deaths | 1.77636E-15 | Total change (desc) | 42 |
| Equatorial Guinea | Central Sub-Saharan Africa | 9.216642747 | 18.25955334 | 9.042910595 | 7.664107409 | -1.614487931 | 2.993291117 | Deaths | Deaths | 0 | Total change (desc) | 43 |
| Djibouti | Eastern Sub-Saharan Africa | 6.179719751 | 14.80836593 | 8.628646178 | 4.380341413 | 2.175269307 | 2.073035458 | Deaths | Deaths | 1.77636E-15 | Total change (desc) | 44 |
| Lesotho | Southern Sub-Saharan Africa | 10.3910404 | 17.39284628 | 7.001805884 | 1.656192402 | 1.134612909 | 4.211000572 | Deaths | Deaths | 0 | Total change (desc) | 45 |
| Namibia | Southern Sub-Saharan Africa | 10.17785793 | 16.86448545 | 6.686627528 | 4.954712424 | 0.253305601 | 1.478609503 | Deaths | Deaths | 0 | Total change (desc) | 46 |
| Guinea-Bissau | Western Sub-Saharan Africa | 8.397210603 | 14.46644316 | 6.069232556 | 3.950671583 | -0.574618185 | 2.693179158 | Deaths | Deaths | 0 | Total change (desc) | 47 |
| Botswana | Southern Sub-Saharan Africa | 3.805531732 | 8.084482262 | 4.278950529 | 1.247420493 | 1.154782893 | 1.876747144 | Deaths | Deaths | -8.88178E-16 | Total change (desc) | 48 |
| Gambia | Western Sub-Saharan Africa | 4.338868405 | 8.412647276 | 4.073778871 | 2.522518549 | -0.221093876 | 1.772354199 | Deaths | Deaths | 0 | Total change (desc) | 49 |
| Comoros | Eastern Sub-Saharan Africa | 3.753267617 | 6.293208457 | 2.53994084 | 1.708816378 | 0.247986165 | 0.583138298 | Deaths | Deaths | 0 | Total change (desc) | 50 |
| Sao Tome and Principe | Central Sub-Saharan Africa | 3.722044871 | 5.65809076 | 1.936045889 | 1.682037235 | -0.173992201 | 0.428000855 | Deaths | Deaths | -2.22045E-16 | Total change (desc) | 51 |
| Cabo Verde | Western Sub-Saharan Africa | 5.943886662 | 7.573860943 | 1.629974281 | 0.44024901 | 0.881595201 | 0.30813007 | Deaths | Deaths | 0 | Total change (desc) | 52 |
| Eswatini | Southern Sub-Saharan Africa | 3.912653768 | 5.280661926 | 1.368008158 | 0.823597928 | 0.741554797 | -0.197144568 | Deaths | Deaths | 0 | Total change (desc) | 53 |
| Seychelles | Eastern Sub-Saharan Africa | 3.54949073 | 4.623414279 | 1.073923548 | 0.591035069 | 0.871006531 | -0.388118052 | Deaths | Deaths | 0 | Total change (desc) | 54 |
| Ethiopia | Eastern Sub-Saharan Africa | 663.9420159 | 1486.747509 | 822.8054935 | 395.6323247 | 32.67336535 | 394.4998034 | Deaths | Deaths | 0 | Epidemiology (desc) | 1 |
| Democratic Republic of the Congo | Central Sub-Saharan Africa | 395.7489603 | 857.4262059 | 461.6772456 | 239.7238718 | 2.982111442 | 218.9712623 | Deaths | Deaths | 0 | Epidemiology (desc) | 2 |
| Nigeria | Western Sub-Saharan Africa | 605.5797419 | 1004.897211 | 399.3174688 | 316.4181903 | -21.6736091 | 104.5728877 | Deaths | Deaths | -5.68434E-14 | Epidemiology (desc) | 3 |
| Ghana | Western Sub-Saharan Africa | 213.767963 | 433.6986218 | 219.9306588 | 102.2572775 | 17.83945789 | 99.83392344 | Deaths | Deaths | 0 | Epidemiology (desc) | 4 |
| Morocco | North Africa | 275.6103019 | 574.4151945 | 298.8048926 | 74.95482001 | 124.5823253 | 99.26774728 | Deaths | Deaths | 0 | Epidemiology (desc) | 5 |
| Malawi | Eastern Sub-Saharan Africa | 270.6739904 | 522.6856891 | 252.0116986 | 175.7920392 | -21.39162952 | 97.61128897 | Deaths | Deaths | 0 | Epidemiology (desc) | 6 |
| Algeria | North Africa | 353.1393255 | 662.8779752 | 309.7386497 | 93.11635927 | 138.5780074 | 78.04428309 | Deaths | Deaths | 0 | Epidemiology (desc) | 7 |
| Mali | Western Sub-Saharan Africa | 276.0451347 | 515.7255284 | 239.6803937 | 169.4067023 | 2.100685145 | 68.17300619 | Deaths | Deaths | 0 | Epidemiology (desc) | 8 |
| Côte d'Ivoire | Western Sub-Saharan Africa | 94.59904622 | 229.5066155 | 134.9075693 | 79.74363641 | -6.517443775 | 61.68137667 | Deaths | Deaths | 0 | Epidemiology (desc) | 9 |
| Madagascar | Eastern Sub-Saharan Africa | 201.5654447 | 367.3474213 | 165.7819766 | 123.479298 | -15.05430979 | 57.35698839 | Deaths | Deaths | 0 | Epidemiology (desc) | 10 |
| Rwanda | Eastern Sub-Saharan Africa | 111.5905961 | 245.9371291 | 134.346533 | 61.6031415 | 32.52658055 | 40.21681095 | Deaths | Deaths | 0 | Epidemiology (desc) | 11 |
| Egypt | North Africa | 1604.010006 | 2670.431297 | 1066.421291 | 548.0600179 | 478.9910484 | 39.37022428 | Deaths | Deaths | 0 | Epidemiology (desc) | 12 |
| Cameroon | Central Sub-Saharan Africa | 176.3110917 | 320.1833949 | 143.8723032 | 112.8353849 | -7.194737363 | 38.23165568 | Deaths | Deaths | 0 | Epidemiology (desc) | 13 |
| Zambia | Eastern Sub-Saharan Africa | 115.6226946 | 235.0729277 | 119.4502331 | 87.73099785 | -4.912323413 | 36.63155864 | Deaths | Deaths | 0 | Epidemiology (desc) | 14 |
| Uganda | Eastern Sub-Saharan Africa | 211.421916 | 375.2067242 | 163.7848082 | 118.876367 | 8.390704424 | 36.5177368 | Deaths | Deaths | 0 | Epidemiology (desc) | 15 |
| South Africa | Southern Sub-Saharan Africa | 707.5486592 | 1086.836772 | 379.2881126 | 249.0200167 | 101.1155688 | 29.15252711 | Deaths | Deaths | 0 | Epidemiology (desc) | 16 |
| Sudan | North Africa | 360.1915736 | 577.4688549 | 217.2772813 | 141.0364511 | 47.49170832 | 28.74912181 | Deaths | Deaths | -2.84217E-14 | Epidemiology (desc) | 17 |
| Burkina Faso | Western Sub-Saharan Africa | 104.8475987 | 182.7620764 | 77.91447775 | 56.89495668 | -2.232892468 | 23.25241354 | Deaths | Deaths | 0 | Epidemiology (desc) | 18 |
| Senegal | Western Sub-Saharan Africa | 80.56879852 | 145.5374269 | 64.96862837 | 47.60969152 | -3.730761452 | 21.0896983 | Deaths | Deaths | 0 | Epidemiology (desc) | 19 |
| Guinea | Western Sub-Saharan Africa | 74.15057462 | 109.2091465 | 35.05857189 | 33.89184398 | -18.30175405 | 19.46848196 | Deaths | Deaths | 0 | Epidemiology (desc) | 20 |
| Tunisia | North Africa | 394.2318743 | 618.7295118 | 224.4976374 | 60.95966928 | 144.4292551 | 19.10871303 | Deaths | Deaths | 0 | Epidemiology (desc) | 21 |
| Kenya | Eastern Sub-Saharan Africa | 137.6824076 | 251.4715164 | 113.7891088 | 67.86628927 | 27.4873498 | 18.43546974 | Deaths | Deaths | -1.42109E-14 | Epidemiology (desc) | 22 |
| Chad | Central Sub-Saharan Africa | 76.25536602 | 129.6464602 | 53.39109416 | 50.00188841 | -14.75444597 | 18.14365172 | Deaths | Deaths | 0 | Epidemiology (desc) | 23 |
| Sierra Leone | Western Sub-Saharan Africa | 53.4484448 | 80.65559964 | 27.20715484 | 23.24299982 | -14.12728686 | 18.09144188 | Deaths | Deaths | 0 | Epidemiology (desc) | 24 |
| Angola | Central Sub-Saharan Africa | 139.7620625 | 267.2352695 | 127.473207 | 97.27011338 | 13.07580814 | 17.12728548 | Deaths | Deaths | -1.42109E-14 | Epidemiology (desc) | 25 |
| Burundi | Eastern Sub-Saharan Africa | 57.26753013 | 107.4562451 | 50.18871495 | 36.11971184 | -2.462954529 | 16.53195764 | Deaths | Deaths | 0 | Epidemiology (desc) | 26 |
| Eritrea | Eastern Sub-Saharan Africa | 37.19520095 | 73.21883494 | 36.02363399 | 15.61878346 | 5.391434886 | 15.01341564 | Deaths | Deaths | 0 | Epidemiology (desc) | 27 |
| Mozambique | Eastern Sub-Saharan Africa | 181.6659995 | 264.7393277 | 83.07332821 | 88.61154457 | -20.06846023 | 14.53024386 | Deaths | Deaths | 1.42109E-14 | Epidemiology (desc) | 28 |
| Niger | Western Sub-Saharan Africa | 74.87761713 | 143.7562413 | 68.87862419 | 53.67468084 | 0.978238183 | 14.22570517 | Deaths | Deaths | 0 | Epidemiology (desc) | 29 |
| Congo | Central Sub-Saharan Africa | 34.00656067 | 65.42464495 | 31.41808428 | 18.03359684 | -0.689987904 | 14.07447535 | Deaths | Deaths | 0 | Epidemiology (desc) | 30 |
| South Sudan | Eastern Sub-Saharan Africa | 74.9716973 | 114.3239522 | 39.3522549 | 13.86410528 | 12.91730297 | 12.57084665 | Deaths | Deaths | 0 | Epidemiology (desc) | 31 |
| Somalia | Eastern Sub-Saharan Africa | 42.39147176 | 86.44942616 | 44.0579544 | 27.49409506 | 4.340777598 | 12.22308174 | Deaths | Deaths | 0 | Epidemiology (desc) | 32 |
| Togo | Western Sub-Saharan Africa | 34.36761729 | 73.04211907 | 38.67450177 | 18.50813685 | 9.036869574 | 11.12949535 | Deaths | Deaths | 0 | Epidemiology (desc) | 33 |
| Gabon | Central Sub-Saharan Africa | 18.03156219 | 33.7517258 | 15.7201636 | 6.926693072 | 1.350942623 | 7.442527907 | Deaths | Deaths | 0 | Epidemiology (desc) | 34 |
| Mauritania | Western Sub-Saharan Africa | 31.91092179 | 55.14717871 | 23.23625692 | 15.29038526 | 0.7861046 | 7.159767062 | Deaths | Deaths | 0 | Epidemiology (desc) | 35 |
| Liberia | Western Sub-Saharan Africa | 33.39817139 | 55.7914315 | 22.3932601 | 21.36484751 | -5.592517492 | 6.620930084 | Deaths | Deaths | 0 | Epidemiology (desc) | 36 |
| Central African Republic | Central Sub-Saharan Africa | 19.50191876 | 35.04542584 | 15.54350708 | 7.714027206 | 2.031746745 | 5.797733128 | Deaths | Deaths | 0 | Epidemiology (desc) | 37 |
| Lesotho | Southern Sub-Saharan Africa | 10.3910404 | 17.39284628 | 7.001805884 | 1.656192402 | 1.134612909 | 4.211000572 | Deaths | Deaths | 0 | Epidemiology (desc) | 38 |
| Equatorial Guinea | Central Sub-Saharan Africa | 9.216642747 | 18.25955334 | 9.042910595 | 7.664107409 | -1.614487931 | 2.993291117 | Deaths | Deaths | 0 | Epidemiology (desc) | 39 |
| Guinea-Bissau | Western Sub-Saharan Africa | 8.397210603 | 14.46644316 | 6.069232556 | 3.950671583 | -0.574618185 | 2.693179158 | Deaths | Deaths | 0 | Epidemiology (desc) | 40 |
| Djibouti | Eastern Sub-Saharan Africa | 6.179719751 | 14.80836593 | 8.628646178 | 4.380341413 | 2.175269307 | 2.073035458 | Deaths | Deaths | 1.77636E-15 | Epidemiology (desc) | 41 |
| Botswana | Southern Sub-Saharan Africa | 3.805531732 | 8.084482262 | 4.278950529 | 1.247420493 | 1.154782893 | 1.876747144 | Deaths | Deaths | -8.88178E-16 | Epidemiology (desc) | 42 |
| Gambia | Western Sub-Saharan Africa | 4.338868405 | 8.412647276 | 4.073778871 | 2.522518549 | -0.221093876 | 1.772354199 | Deaths | Deaths | 0 | Epidemiology (desc) | 43 |
| Namibia | Southern Sub-Saharan Africa | 10.17785793 | 16.86448545 | 6.686627528 | 4.954712424 | 0.253305601 | 1.478609503 | Deaths | Deaths | 0 | Epidemiology (desc) | 44 |
| Benin | Western Sub-Saharan Africa | 30.02094939 | 49.58943339 | 19.568484 | 18.39233551 | -0.257387797 | 1.433536286 | Deaths | Deaths | 0 | Epidemiology (desc) | 45 |
| Comoros | Eastern Sub-Saharan Africa | 3.753267617 | 6.293208457 | 2.53994084 | 1.708816378 | 0.247986165 | 0.583138298 | Deaths | Deaths | 0 | Epidemiology (desc) | 46 |
| Zimbabwe | Southern Sub-Saharan Africa | 173.2875841 | 250.3970836 | 77.10949947 | 45.92833623 | 30.64043003 | 0.540733199 | Deaths | Deaths | 0 | Epidemiology (desc) | 47 |
| Sao Tome and Principe | Central Sub-Saharan Africa | 3.722044871 | 5.65809076 | 1.936045889 | 1.682037235 | -0.173992201 | 0.428000855 | Deaths | Deaths | -2.22045E-16 | Epidemiology (desc) | 48 |
| Cabo Verde | Western Sub-Saharan Africa | 5.943886662 | 7.573860943 | 1.629974281 | 0.44024901 | 0.881595201 | 0.30813007 | Deaths | Deaths | 0 | Epidemiology (desc) | 49 |
| Eswatini | Southern Sub-Saharan Africa | 3.912653768 | 5.280661926 | 1.368008158 | 0.823597928 | 0.741554797 | -0.197144568 | Deaths | Deaths | 0 | Epidemiology (desc) | 50 |
| Seychelles | Eastern Sub-Saharan Africa | 3.54949073 | 4.623414279 | 1.073923548 | 0.591035069 | 0.871006531 | -0.388118052 | Deaths | Deaths | 0 | Epidemiology (desc) | 51 |
| Mauritius | Eastern Sub-Saharan Africa | 26.21870696 | 37.0870244 | 10.86831744 | 2.180648445 | 11.09044705 | -2.402778055 | Deaths | Deaths | 1.77636E-15 | Epidemiology (desc) | 52 |
| Libya | North Africa | 164.6956096 | 257.3953631 | 92.69975352 | 48.27471783 | 51.97900393 | -7.553968245 | Deaths | Deaths | 0 | Epidemiology (desc) | 53 |
| United Republic of Tanzania | Eastern Sub-Saharan Africa | 455.7458281 | 684.6217131 | 228.875885 | 241.6135377 | -0.541053895 | -12.19659879 | Deaths | Deaths | 2.84217E-14 | Epidemiology (desc) | 54 |
| Egypt | North Africa | 1604.010006 | 2670.431297 | 1066.421291 | 548.0600179 | 478.9910484 | 39.37022428 | Deaths | Deaths | 0 | Aging/Structure (desc) | 1 |
| Tunisia | North Africa | 394.2318743 | 618.7295118 | 224.4976374 | 60.95966928 | 144.4292551 | 19.10871303 | Deaths | Deaths | 0 | Aging/Structure (desc) | 2 |
| Algeria | North Africa | 353.1393255 | 662.8779752 | 309.7386497 | 93.11635927 | 138.5780074 | 78.04428309 | Deaths | Deaths | 0 | Aging/Structure (desc) | 3 |
| Morocco | North Africa | 275.6103019 | 574.4151945 | 298.8048926 | 74.95482001 | 124.5823253 | 99.26774728 | Deaths | Deaths | 0 | Aging/Structure (desc) | 4 |
| South Africa | Southern Sub-Saharan Africa | 707.5486592 | 1086.836772 | 379.2881126 | 249.0200167 | 101.1155688 | 29.15252711 | Deaths | Deaths | 0 | Aging/Structure (desc) | 5 |
| Libya | North Africa | 164.6956096 | 257.3953631 | 92.69975352 | 48.27471783 | 51.97900393 | -7.553968245 | Deaths | Deaths | 0 | Aging/Structure (desc) | 6 |
| Sudan | North Africa | 360.1915736 | 577.4688549 | 217.2772813 | 141.0364511 | 47.49170832 | 28.74912181 | Deaths | Deaths | -2.84217E-14 | Aging/Structure (desc) | 7 |
| Ethiopia | Eastern Sub-Saharan Africa | 663.9420159 | 1486.747509 | 822.8054935 | 395.6323247 | 32.67336535 | 394.4998034 | Deaths | Deaths | 0 | Aging/Structure (desc) | 8 |
| Rwanda | Eastern Sub-Saharan Africa | 111.5905961 | 245.9371291 | 134.346533 | 61.6031415 | 32.52658055 | 40.21681095 | Deaths | Deaths | 0 | Aging/Structure (desc) | 9 |
| Zimbabwe | Southern Sub-Saharan Africa | 173.2875841 | 250.3970836 | 77.10949947 | 45.92833623 | 30.64043003 | 0.540733199 | Deaths | Deaths | 0 | Aging/Structure (desc) | 10 |
| Kenya | Eastern Sub-Saharan Africa | 137.6824076 | 251.4715164 | 113.7891088 | 67.86628927 | 27.4873498 | 18.43546974 | Deaths | Deaths | -1.42109E-14 | Aging/Structure (desc) | 11 |
| Ghana | Western Sub-Saharan Africa | 213.767963 | 433.6986218 | 219.9306588 | 102.2572775 | 17.83945789 | 99.83392344 | Deaths | Deaths | 0 | Aging/Structure (desc) | 12 |
| Angola | Central Sub-Saharan Africa | 139.7620625 | 267.2352695 | 127.473207 | 97.27011338 | 13.07580814 | 17.12728548 | Deaths | Deaths | -1.42109E-14 | Aging/Structure (desc) | 13 |
| South Sudan | Eastern Sub-Saharan Africa | 74.9716973 | 114.3239522 | 39.3522549 | 13.86410528 | 12.91730297 | 12.57084665 | Deaths | Deaths | 0 | Aging/Structure (desc) | 14 |
| Mauritius | Eastern Sub-Saharan Africa | 26.21870696 | 37.0870244 | 10.86831744 | 2.180648445 | 11.09044705 | -2.402778055 | Deaths | Deaths | 1.77636E-15 | Aging/Structure (desc) | 15 |
| Togo | Western Sub-Saharan Africa | 34.36761729 | 73.04211907 | 38.67450177 | 18.50813685 | 9.036869574 | 11.12949535 | Deaths | Deaths | 0 | Aging/Structure (desc) | 16 |
| Uganda | Eastern Sub-Saharan Africa | 211.421916 | 375.2067242 | 163.7848082 | 118.876367 | 8.390704424 | 36.5177368 | Deaths | Deaths | 0 | Aging/Structure (desc) | 17 |
| Eritrea | Eastern Sub-Saharan Africa | 37.19520095 | 73.21883494 | 36.02363399 | 15.61878346 | 5.391434886 | 15.01341564 | Deaths | Deaths | 0 | Aging/Structure (desc) | 18 |
| Somalia | Eastern Sub-Saharan Africa | 42.39147176 | 86.44942616 | 44.0579544 | 27.49409506 | 4.340777598 | 12.22308174 | Deaths | Deaths | 0 | Aging/Structure (desc) | 19 |
| Democratic Republic of the Congo | Central Sub-Saharan Africa | 395.7489603 | 857.4262059 | 461.6772456 | 239.7238718 | 2.982111442 | 218.9712623 | Deaths | Deaths | 0 | Aging/Structure (desc) | 20 |
| Djibouti | Eastern Sub-Saharan Africa | 6.179719751 | 14.80836593 | 8.628646178 | 4.380341413 | 2.175269307 | 2.073035458 | Deaths | Deaths | 1.77636E-15 | Aging/Structure (desc) | 21 |
| Mali | Western Sub-Saharan Africa | 276.0451347 | 515.7255284 | 239.6803937 | 169.4067023 | 2.100685145 | 68.17300619 | Deaths | Deaths | 0 | Aging/Structure (desc) | 22 |
| Central African Republic | Central Sub-Saharan Africa | 19.50191876 | 35.04542584 | 15.54350708 | 7.714027206 | 2.031746745 | 5.797733128 | Deaths | Deaths | 0 | Aging/Structure (desc) | 23 |
| Gabon | Central Sub-Saharan Africa | 18.03156219 | 33.7517258 | 15.7201636 | 6.926693072 | 1.350942623 | 7.442527907 | Deaths | Deaths | 0 | Aging/Structure (desc) | 24 |
| Botswana | Southern Sub-Saharan Africa | 3.805531732 | 8.084482262 | 4.278950529 | 1.247420493 | 1.154782893 | 1.876747144 | Deaths | Deaths | -8.88178E-16 | Aging/Structure (desc) | 25 |
| Lesotho | Southern Sub-Saharan Africa | 10.3910404 | 17.39284628 | 7.001805884 | 1.656192402 | 1.134612909 | 4.211000572 | Deaths | Deaths | 0 | Aging/Structure (desc) | 26 |
| Niger | Western Sub-Saharan Africa | 74.87761713 | 143.7562413 | 68.87862419 | 53.67468084 | 0.978238183 | 14.22570517 | Deaths | Deaths | 0 | Aging/Structure (desc) | 27 |
| Cabo Verde | Western Sub-Saharan Africa | 5.943886662 | 7.573860943 | 1.629974281 | 0.44024901 | 0.881595201 | 0.30813007 | Deaths | Deaths | 0 | Aging/Structure (desc) | 28 |
| Seychelles | Eastern Sub-Saharan Africa | 3.54949073 | 4.623414279 | 1.073923548 | 0.591035069 | 0.871006531 | -0.388118052 | Deaths | Deaths | 0 | Aging/Structure (desc) | 29 |
| Mauritania | Western Sub-Saharan Africa | 31.91092179 | 55.14717871 | 23.23625692 | 15.29038526 | 0.7861046 | 7.159767062 | Deaths | Deaths | 0 | Aging/Structure (desc) | 30 |
| Eswatini | Southern Sub-Saharan Africa | 3.912653768 | 5.280661926 | 1.368008158 | 0.823597928 | 0.741554797 | -0.197144568 | Deaths | Deaths | 0 | Aging/Structure (desc) | 31 |
| Namibia | Southern Sub-Saharan Africa | 10.17785793 | 16.86448545 | 6.686627528 | 4.954712424 | 0.253305601 | 1.478609503 | Deaths | Deaths | 0 | Aging/Structure (desc) | 32 |
| Comoros | Eastern Sub-Saharan Africa | 3.753267617 | 6.293208457 | 2.53994084 | 1.708816378 | 0.247986165 | 0.583138298 | Deaths | Deaths | 0 | Aging/Structure (desc) | 33 |
| Sao Tome and Principe | Central Sub-Saharan Africa | 3.722044871 | 5.65809076 | 1.936045889 | 1.682037235 | -0.173992201 | 0.428000855 | Deaths | Deaths | -2.22045E-16 | Aging/Structure (desc) | 34 |
| Gambia | Western Sub-Saharan Africa | 4.338868405 | 8.412647276 | 4.073778871 | 2.522518549 | -0.221093876 | 1.772354199 | Deaths | Deaths | 0 | Aging/Structure (desc) | 35 |
| Benin | Western Sub-Saharan Africa | 30.02094939 | 49.58943339 | 19.568484 | 18.39233551 | -0.257387797 | 1.433536286 | Deaths | Deaths | 0 | Aging/Structure (desc) | 36 |
| United Republic of Tanzania | Eastern Sub-Saharan Africa | 455.7458281 | 684.6217131 | 228.875885 | 241.6135377 | -0.541053895 | -12.19659879 | Deaths | Deaths | 2.84217E-14 | Aging/Structure (desc) | 37 |
| Guinea-Bissau | Western Sub-Saharan Africa | 8.397210603 | 14.46644316 | 6.069232556 | 3.950671583 | -0.574618185 | 2.693179158 | Deaths | Deaths | 0 | Aging/Structure (desc) | 38 |
| Congo | Central Sub-Saharan Africa | 34.00656067 | 65.42464495 | 31.41808428 | 18.03359684 | -0.689987904 | 14.07447535 | Deaths | Deaths | 0 | Aging/Structure (desc) | 39 |
| Equatorial Guinea | Central Sub-Saharan Africa | 9.216642747 | 18.25955334 | 9.042910595 | 7.664107409 | -1.614487931 | 2.993291117 | Deaths | Deaths | 0 | Aging/Structure (desc) | 40 |
| Burkina Faso | Western Sub-Saharan Africa | 104.8475987 | 182.7620764 | 77.91447775 | 56.89495668 | -2.232892468 | 23.25241354 | Deaths | Deaths | 0 | Aging/Structure (desc) | 41 |
| Burundi | Eastern Sub-Saharan Africa | 57.26753013 | 107.4562451 | 50.18871495 | 36.11971184 | -2.462954529 | 16.53195764 | Deaths | Deaths | 0 | Aging/Structure (desc) | 42 |
| Senegal | Western Sub-Saharan Africa | 80.56879852 | 145.5374269 | 64.96862837 | 47.60969152 | -3.730761452 | 21.0896983 | Deaths | Deaths | 0 | Aging/Structure (desc) | 43 |
| Zambia | Eastern Sub-Saharan Africa | 115.6226946 | 235.0729277 | 119.4502331 | 87.73099785 | -4.912323413 | 36.63155864 | Deaths | Deaths | 0 | Aging/Structure (desc) | 44 |
| Liberia | Western Sub-Saharan Africa | 33.39817139 | 55.7914315 | 22.3932601 | 21.36484751 | -5.592517492 | 6.620930084 | Deaths | Deaths | 0 | Aging/Structure (desc) | 45 |
| Côte d'Ivoire | Western Sub-Saharan Africa | 94.59904622 | 229.5066155 | 134.9075693 | 79.74363641 | -6.517443775 | 61.68137667 | Deaths | Deaths | 0 | Aging/Structure (desc) | 46 |
| Cameroon | Central Sub-Saharan Africa | 176.3110917 | 320.1833949 | 143.8723032 | 112.8353849 | -7.194737363 | 38.23165568 | Deaths | Deaths | 0 | Aging/Structure (desc) | 47 |
| Sierra Leone | Western Sub-Saharan Africa | 53.4484448 | 80.65559964 | 27.20715484 | 23.24299982 | -14.12728686 | 18.09144188 | Deaths | Deaths | 0 | Aging/Structure (desc) | 48 |
| Chad | Central Sub-Saharan Africa | 76.25536602 | 129.6464602 | 53.39109416 | 50.00188841 | -14.75444597 | 18.14365172 | Deaths | Deaths | 0 | Aging/Structure (desc) | 49 |
| Madagascar | Eastern Sub-Saharan Africa | 201.5654447 | 367.3474213 | 165.7819766 | 123.479298 | -15.05430979 | 57.35698839 | Deaths | Deaths | 0 | Aging/Structure (desc) | 50 |
| Guinea | Western Sub-Saharan Africa | 74.15057462 | 109.2091465 | 35.05857189 | 33.89184398 | -18.30175405 | 19.46848196 | Deaths | Deaths | 0 | Aging/Structure (desc) | 51 |
| Mozambique | Eastern Sub-Saharan Africa | 181.6659995 | 264.7393277 | 83.07332821 | 88.61154457 | -20.06846023 | 14.53024386 | Deaths | Deaths | 1.42109E-14 | Aging/Structure (desc) | 52 |
| Malawi | Eastern Sub-Saharan Africa | 270.6739904 | 522.6856891 | 252.0116986 | 175.7920392 | -21.39162952 | 97.61128897 | Deaths | Deaths | 0 | Aging/Structure (desc) | 53 |
| Nigeria | Western Sub-Saharan Africa | 605.5797419 | 1004.897211 | 399.3174688 | 316.4181903 | -21.6736091 | 104.5728877 | Deaths | Deaths | -5.68434E-14 | Aging/Structure (desc) | 54 |
| Egypt | North Africa | 1604.010006 | 2670.431297 | 1066.421291 | 548.0600179 | 478.9910484 | 39.37022428 | Deaths | Deaths | 0 | Population (desc) | 1 |
| Ethiopia | Eastern Sub-Saharan Africa | 663.9420159 | 1486.747509 | 822.8054935 | 395.6323247 | 32.67336535 | 394.4998034 | Deaths | Deaths | 0 | Population (desc) | 2 |
| Nigeria | Western Sub-Saharan Africa | 605.5797419 | 1004.897211 | 399.3174688 | 316.4181903 | -21.6736091 | 104.5728877 | Deaths | Deaths | -5.68434E-14 | Population (desc) | 3 |
| South Africa | Southern Sub-Saharan Africa | 707.5486592 | 1086.836772 | 379.2881126 | 249.0200167 | 101.1155688 | 29.15252711 | Deaths | Deaths | 0 | Population (desc) | 4 |
| United Republic of Tanzania | Eastern Sub-Saharan Africa | 455.7458281 | 684.6217131 | 228.875885 | 241.6135377 | -0.541053895 | -12.19659879 | Deaths | Deaths | 2.84217E-14 | Population (desc) | 5 |
| Democratic Republic of the Congo | Central Sub-Saharan Africa | 395.7489603 | 857.4262059 | 461.6772456 | 239.7238718 | 2.982111442 | 218.9712623 | Deaths | Deaths | 0 | Population (desc) | 6 |
| Malawi | Eastern Sub-Saharan Africa | 270.6739904 | 522.6856891 | 252.0116986 | 175.7920392 | -21.39162952 | 97.61128897 | Deaths | Deaths | 0 | Population (desc) | 7 |
| Mali | Western Sub-Saharan Africa | 276.0451347 | 515.7255284 | 239.6803937 | 169.4067023 | 2.100685145 | 68.17300619 | Deaths | Deaths | 0 | Population (desc) | 8 |
| Sudan | North Africa | 360.1915736 | 577.4688549 | 217.2772813 | 141.0364511 | 47.49170832 | 28.74912181 | Deaths | Deaths | -2.84217E-14 | Population (desc) | 9 |
| Madagascar | Eastern Sub-Saharan Africa | 201.5654447 | 367.3474213 | 165.7819766 | 123.479298 | -15.05430979 | 57.35698839 | Deaths | Deaths | 0 | Population (desc) | 10 |
| Uganda | Eastern Sub-Saharan Africa | 211.421916 | 375.2067242 | 163.7848082 | 118.876367 | 8.390704424 | 36.5177368 | Deaths | Deaths | 0 | Population (desc) | 11 |
| Cameroon | Central Sub-Saharan Africa | 176.3110917 | 320.1833949 | 143.8723032 | 112.8353849 | -7.194737363 | 38.23165568 | Deaths | Deaths | 0 | Population (desc) | 12 |
| Ghana | Western Sub-Saharan Africa | 213.767963 | 433.6986218 | 219.9306588 | 102.2572775 | 17.83945789 | 99.83392344 | Deaths | Deaths | 0 | Population (desc) | 13 |
| Angola | Central Sub-Saharan Africa | 139.7620625 | 267.2352695 | 127.473207 | 97.27011338 | 13.07580814 | 17.12728548 | Deaths | Deaths | -1.42109E-14 | Population (desc) | 14 |
| Algeria | North Africa | 353.1393255 | 662.8779752 | 309.7386497 | 93.11635927 | 138.5780074 | 78.04428309 | Deaths | Deaths | 0 | Population (desc) | 15 |
| Mozambique | Eastern Sub-Saharan Africa | 181.6659995 | 264.7393277 | 83.07332821 | 88.61154457 | -20.06846023 | 14.53024386 | Deaths | Deaths | 1.42109E-14 | Population (desc) | 16 |
| Zambia | Eastern Sub-Saharan Africa | 115.6226946 | 235.0729277 | 119.4502331 | 87.73099785 | -4.912323413 | 36.63155864 | Deaths | Deaths | 0 | Population (desc) | 17 |
| Côte d'Ivoire | Western Sub-Saharan Africa | 94.59904622 | 229.5066155 | 134.9075693 | 79.74363641 | -6.517443775 | 61.68137667 | Deaths | Deaths | 0 | Population (desc) | 18 |
| Morocco | North Africa | 275.6103019 | 574.4151945 | 298.8048926 | 74.95482001 | 124.5823253 | 99.26774728 | Deaths | Deaths | 0 | Population (desc) | 19 |
| Kenya | Eastern Sub-Saharan Africa | 137.6824076 | 251.4715164 | 113.7891088 | 67.86628927 | 27.4873498 | 18.43546974 | Deaths | Deaths | -1.42109E-14 | Population (desc) | 20 |
| Rwanda | Eastern Sub-Saharan Africa | 111.5905961 | 245.9371291 | 134.346533 | 61.6031415 | 32.52658055 | 40.21681095 | Deaths | Deaths | 0 | Population (desc) | 21 |
| Tunisia | North Africa | 394.2318743 | 618.7295118 | 224.4976374 | 60.95966928 | 144.4292551 | 19.10871303 | Deaths | Deaths | 0 | Population (desc) | 22 |
| Burkina Faso | Western Sub-Saharan Africa | 104.8475987 | 182.7620764 | 77.91447775 | 56.89495668 | -2.232892468 | 23.25241354 | Deaths | Deaths | 0 | Population (desc) | 23 |
| Niger | Western Sub-Saharan Africa | 74.87761713 | 143.7562413 | 68.87862419 | 53.67468084 | 0.978238183 | 14.22570517 | Deaths | Deaths | 0 | Population (desc) | 24 |
| Chad | Central Sub-Saharan Africa | 76.25536602 | 129.6464602 | 53.39109416 | 50.00188841 | -14.75444597 | 18.14365172 | Deaths | Deaths | 0 | Population (desc) | 25 |
| Libya | North Africa | 164.6956096 | 257.3953631 | 92.69975352 | 48.27471783 | 51.97900393 | -7.553968245 | Deaths | Deaths | 0 | Population (desc) | 26 |
| Senegal | Western Sub-Saharan Africa | 80.56879852 | 145.5374269 | 64.96862837 | 47.60969152 | -3.730761452 | 21.0896983 | Deaths | Deaths | 0 | Population (desc) | 27 |
| Zimbabwe | Southern Sub-Saharan Africa | 173.2875841 | 250.3970836 | 77.10949947 | 45.92833623 | 30.64043003 | 0.540733199 | Deaths | Deaths | 0 | Population (desc) | 28 |
| Burundi | Eastern Sub-Saharan Africa | 57.26753013 | 107.4562451 | 50.18871495 | 36.11971184 | -2.462954529 | 16.53195764 | Deaths | Deaths | 0 | Population (desc) | 29 |
| Guinea | Western Sub-Saharan Africa | 74.15057462 | 109.2091465 | 35.05857189 | 33.89184398 | -18.30175405 | 19.46848196 | Deaths | Deaths | 0 | Population (desc) | 30 |
| Somalia | Eastern Sub-Saharan Africa | 42.39147176 | 86.44942616 | 44.0579544 | 27.49409506 | 4.340777598 | 12.22308174 | Deaths | Deaths | 0 | Population (desc) | 31 |
| Sierra Leone | Western Sub-Saharan Africa | 53.4484448 | 80.65559964 | 27.20715484 | 23.24299982 | -14.12728686 | 18.09144188 | Deaths | Deaths | 0 | Population (desc) | 32 |
| Liberia | Western Sub-Saharan Africa | 33.39817139 | 55.7914315 | 22.3932601 | 21.36484751 | -5.592517492 | 6.620930084 | Deaths | Deaths | 0 | Population (desc) | 33 |
| Togo | Western Sub-Saharan Africa | 34.36761729 | 73.04211907 | 38.67450177 | 18.50813685 | 9.036869574 | 11.12949535 | Deaths | Deaths | 0 | Population (desc) | 34 |
| Benin | Western Sub-Saharan Africa | 30.02094939 | 49.58943339 | 19.568484 | 18.39233551 | -0.257387797 | 1.433536286 | Deaths | Deaths | 0 | Population (desc) | 35 |
| Congo | Central Sub-Saharan Africa | 34.00656067 | 65.42464495 | 31.41808428 | 18.03359684 | -0.689987904 | 14.07447535 | Deaths | Deaths | 0 | Population (desc) | 36 |
| Eritrea | Eastern Sub-Saharan Africa | 37.19520095 | 73.21883494 | 36.02363399 | 15.61878346 | 5.391434886 | 15.01341564 | Deaths | Deaths | 0 | Population (desc) | 37 |
| Mauritania | Western Sub-Saharan Africa | 31.91092179 | 55.14717871 | 23.23625692 | 15.29038526 | 0.7861046 | 7.159767062 | Deaths | Deaths | 0 | Population (desc) | 38 |
| South Sudan | Eastern Sub-Saharan Africa | 74.9716973 | 114.3239522 | 39.3522549 | 13.86410528 | 12.91730297 | 12.57084665 | Deaths | Deaths | 0 | Population (desc) | 39 |
| Central African Republic | Central Sub-Saharan Africa | 19.50191876 | 35.04542584 | 15.54350708 | 7.714027206 | 2.031746745 | 5.797733128 | Deaths | Deaths | 0 | Population (desc) | 40 |
| Equatorial Guinea | Central Sub-Saharan Africa | 9.216642747 | 18.25955334 | 9.042910595 | 7.664107409 | -1.614487931 | 2.993291117 | Deaths | Deaths | 0 | Population (desc) | 41 |
| Gabon | Central Sub-Saharan Africa | 18.03156219 | 33.7517258 | 15.7201636 | 6.926693072 | 1.350942623 | 7.442527907 | Deaths | Deaths | 0 | Population (desc) | 42 |
| Namibia | Southern Sub-Saharan Africa | 10.17785793 | 16.86448545 | 6.686627528 | 4.954712424 | 0.253305601 | 1.478609503 | Deaths | Deaths | 0 | Population (desc) | 43 |
| Djibouti | Eastern Sub-Saharan Africa | 6.179719751 | 14.80836593 | 8.628646178 | 4.380341413 | 2.175269307 | 2.073035458 | Deaths | Deaths | 1.77636E-15 | Population (desc) | 44 |
| Guinea-Bissau | Western Sub-Saharan Africa | 8.397210603 | 14.46644316 | 6.069232556 | 3.950671583 | -0.574618185 | 2.693179158 | Deaths | Deaths | 0 | Population (desc) | 45 |
| Gambia | Western Sub-Saharan Africa | 4.338868405 | 8.412647276 | 4.073778871 | 2.522518549 | -0.221093876 | 1.772354199 | Deaths | Deaths | 0 | Population (desc) | 46 |
| Mauritius | Eastern Sub-Saharan Africa | 26.21870696 | 37.0870244 | 10.86831744 | 2.180648445 | 11.09044705 | -2.402778055 | Deaths | Deaths | 1.77636E-15 | Population (desc) | 47 |
| Comoros | Eastern Sub-Saharan Africa | 3.753267617 | 6.293208457 | 2.53994084 | 1.708816378 | 0.247986165 | 0.583138298 | Deaths | Deaths | 0 | Population (desc) | 48 |
| Sao Tome and Principe | Central Sub-Saharan Africa | 3.722044871 | 5.65809076 | 1.936045889 | 1.682037235 | -0.173992201 | 0.428000855 | Deaths | Deaths | -2.22045E-16 | Population (desc) | 49 |
| Lesotho | Southern Sub-Saharan Africa | 10.3910404 | 17.39284628 | 7.001805884 | 1.656192402 | 1.134612909 | 4.211000572 | Deaths | Deaths | 0 | Population (desc) | 50 |
| Botswana | Southern Sub-Saharan Africa | 3.805531732 | 8.084482262 | 4.278950529 | 1.247420493 | 1.154782893 | 1.876747144 | Deaths | Deaths | -8.88178E-16 | Population (desc) | 51 |
| Eswatini | Southern Sub-Saharan Africa | 3.912653768 | 5.280661926 | 1.368008158 | 0.823597928 | 0.741554797 | -0.197144568 | Deaths | Deaths | 0 | Population (desc) | 52 |
| Seychelles | Eastern Sub-Saharan Africa | 3.54949073 | 4.623414279 | 1.073923548 | 0.591035069 | 0.871006531 | -0.388118052 | Deaths | Deaths | 0 | Population (desc) | 53 |
| Cabo Verde | Western Sub-Saharan Africa | 5.943886662 | 7.573860943 | 1.629974281 | 0.44024901 | 0.881595201 | 0.30813007 | Deaths | Deaths | 0 | Population (desc) | 54 |
| Egypt | North Africa | 43026.32619 | 66393.86757 | 23367.54138 | 14067.67307 | 10626.47184 | -1326.603531 | DALYs | DALYs (Disability-Adjusted Life Years) | 0 | Total change (desc) | 1 |
| Ethiopia | Eastern Sub-Saharan Africa | 16468.6494 | 38553.59226 | 22084.94286 | 7479.634204 | 3406.583381 | 11198.72528 | DALYs | DALYs (Disability-Adjusted Life Years) | 0 | Total change (desc) | 2 |
| Democratic Republic of the Congo | Central Sub-Saharan Africa | 9982.211882 | 22415.98452 | 12433.77264 | 5401.259885 | 1031.717578 | 6000.795172 | DALYs | DALYs (Disability-Adjusted Life Years) | -1.81899E-12 | Total change (desc) | 3 |
| Nigeria | Western Sub-Saharan Africa | 14463.239 | 24391.81334 | 9928.574342 | 7054.925951 | 463.7195619 | 2409.928828 | DALYs | DALYs (Disability-Adjusted Life Years) | 0 | Total change (desc) | 4 |
| South Africa | Southern Sub-Saharan Africa | 17808.40739 | 27424.53015 | 9616.122751 | 5100.922262 | 3942.775619 | 572.4248698 | DALYs | DALYs (Disability-Adjusted Life Years) | 0 | Total change (desc) | 5 |
| Malawi | Eastern Sub-Saharan Africa | 7415.207737 | 15338.10604 | 7922.898308 | 3835.705021 | 888.7298494 | 3198.463438 | DALYs | DALYs (Disability-Adjusted Life Years) | 0 | Total change (desc) | 6 |
| Algeria | North Africa | 7861.496312 | 14835.42164 | 6973.925329 | 2225.242362 | 2893.756512 | 1854.926454 | DALYs | DALYs (Disability-Adjusted Life Years) | 0 | Total change (desc) | 7 |
| Morocco | North Africa | 5954.883097 | 12413.81661 | 6458.933518 | 827.8527404 | 3259.518177 | 2371.5626 | DALYs | DALYs (Disability-Adjusted Life Years) | 0 | Total change (desc) | 8 |
| Ghana | Western Sub-Saharan Africa | 6123.092433 | 12463.68958 | 6340.597146 | 2413.472269 | 931.2279969 | 2995.896881 | DALYs | DALYs (Disability-Adjusted Life Years) | 0 | Total change (desc) | 9 |
| Mali | Western Sub-Saharan Africa | 7216.496164 | 13382.1519 | 6165.655735 | 4103.094345 | 140.8849969 | 1921.676394 | DALYs | DALYs (Disability-Adjusted Life Years) | 0 | Total change (desc) | 10 |
| Tunisia | North Africa | 8455.38744 | 14088.82927 | 5633.441831 | 1111.740689 | 3396.842566 | 1124.858575 | DALYs | DALYs (Disability-Adjusted Life Years) | 0 | Total change (desc) | 11 |
| Sudan | North Africa | 8380.966471 | 13549.35553 | 5168.389055 | 2225.859312 | 2134.979566 | 807.5501774 | DALYs | DALYs (Disability-Adjusted Life Years) | 0 | Total change (desc) | 12 |
| United Republic of Tanzania | Eastern Sub-Saharan Africa | 11037.80565 | 16162.3224 | 5124.516747 | 5096.211073 | 947.2228104 | -918.917137 | DALYs | DALYs (Disability-Adjusted Life Years) | 9.09495E-13 | Total change (desc) | 13 |
| Madagascar | Eastern Sub-Saharan Africa | 5301.291837 | 10228.28459 | 4926.992757 | 2652.074581 | 549.1577272 | 1725.760448 | DALYs | DALYs (Disability-Adjusted Life Years) | 0 | Total change (desc) | 14 |
| Uganda | Eastern Sub-Saharan Africa | 5612.168992 | 10240.16815 | 4627.999163 | 2525.174844 | 948.6554191 | 1154.168899 | DALYs | DALYs (Disability-Adjusted Life Years) | 0 | Total change (desc) | 15 |
| Cameroon | Central Sub-Saharan Africa | 4301.222783 | 8254.932131 | 3953.709349 | 2572.964132 | 233.1063527 | 1147.638864 | DALYs | DALYs (Disability-Adjusted Life Years) | 0 | Total change (desc) | 16 |
| Côte d'Ivoire | Western Sub-Saharan Africa | 2312.361298 | 6163.142699 | 3850.781401 | 1866.46353 | 108.8594736 | 1875.458398 | DALYs | DALYs (Disability-Adjusted Life Years) | 0 | Total change (desc) | 17 |
| Rwanda | Eastern Sub-Saharan Africa | 2911.496978 | 6726.404487 | 3814.907509 | 1298.907247 | 1182.242739 | 1333.757523 | DALYs | DALYs (Disability-Adjusted Life Years) | 0 | Total change (desc) | 18 |
| Zambia | Eastern Sub-Saharan Africa | 3399.003624 | 7004.547015 | 3605.543392 | 2262.428124 | 317.2085369 | 1025.906731 | DALYs | DALYs (Disability-Adjusted Life Years) | 0 | Total change (desc) | 19 |
| Angola | Central Sub-Saharan Africa | 3618.224072 | 7066.055622 | 3447.83155 | 2361.349449 | 512.1471917 | 574.3349087 | DALYs | DALYs (Disability-Adjusted Life Years) | 0 | Total change (desc) | 20 |
| Kenya | Eastern Sub-Saharan Africa | 3610.470243 | 6551.855393 | 2941.38515 | 1184.52625 | 1235.365899 | 521.4930007 | DALYs | DALYs (Disability-Adjusted Life Years) | 0 | Total change (desc) | 21 |
| Libya | North Africa | 4020.257513 | 6332.613919 | 2312.356406 | 863.0277573 | 1809.254918 | -359.9262701 | DALYs | DALYs (Disability-Adjusted Life Years) | 0 | Total change (desc) | 22 |
| Mozambique | Eastern Sub-Saharan Africa | 4403.573833 | 6260.51078 | 1856.936947 | 1928.913936 | -180.3201093 | 108.3431199 | DALYs | DALYs (Disability-Adjusted Life Years) | -2.27374E-13 | Total change (desc) | 23 |
| Zimbabwe | Southern Sub-Saharan Africa | 4647.927977 | 6428.346121 | 1780.418144 | 1000.03252 | 1060.606998 | -280.2213738 | DALYs | DALYs (Disability-Adjusted Life Years) | 0 | Total change (desc) | 24 |
| Burundi | Eastern Sub-Saharan Africa | 1543.613458 | 3203.068115 | 1659.454657 | 964.8890823 | 96.34714611 | 598.218429 | DALYs | DALYs (Disability-Adjusted Life Years) | -2.27374E-13 | Total change (desc) | 25 |
| Burkina Faso | Western Sub-Saharan Africa | 2315.610136 | 3967.622079 | 1652.011943 | 1112.458116 | 89.91178474 | 449.642043 | DALYs | DALYs (Disability-Adjusted Life Years) | 0 | Total change (desc) | 26 |
| Niger | Western Sub-Saharan Africa | 1835.355376 | 3487.270286 | 1651.91491 | 1251.22093 | 5.282946164 | 395.4110333 | DALYs | DALYs (Disability-Adjusted Life Years) | 0 | Total change (desc) | 27 |
| Senegal | Western Sub-Saharan Africa | 1866.492117 | 3440.282665 | 1573.790548 | 1020.556534 | 24.92628407 | 528.3077294 | DALYs | DALYs (Disability-Adjusted Life Years) | 0 | Total change (desc) | 28 |
| Chad | Central Sub-Saharan Africa | 1804.794537 | 3166.772409 | 1361.977872 | 1186.475185 | -255.5169748 | 431.0196621 | DALYs | DALYs (Disability-Adjusted Life Years) | 0 | Total change (desc) | 29 |
| South Sudan | Eastern Sub-Saharan Africa | 2149.588803 | 3371.529412 | 1221.940609 | 160.5873586 | 636.5702262 | 424.7830248 | DALYs | DALYs (Disability-Adjusted Life Years) | 0 | Total change (desc) | 30 |
| Somalia | Eastern Sub-Saharan Africa | 1155.237536 | 2243.810143 | 1088.572607 | 711.8802814 | 42.48837073 | 334.2039551 | DALYs | DALYs (Disability-Adjusted Life Years) | 0 | Total change (desc) | 31 |
| Eritrea | Eastern Sub-Saharan Africa | 1081.794227 | 2120.207098 | 1038.412871 | 387.6228846 | 186.1744332 | 464.6155532 | DALYs | DALYs (Disability-Adjusted Life Years) | 0 | Total change (desc) | 32 |
| Togo | Western Sub-Saharan Africa | 899.5612754 | 1901.975588 | 1002.414313 | 386.1035389 | 295.6184836 | 320.69229 | DALYs | DALYs (Disability-Adjusted Life Years) | -1.13687E-13 | Total change (desc) | 33 |
| Guinea | Western Sub-Saharan Africa | 1633.266789 | 2520.655897 | 887.3891084 | 714.9902239 | -249.6901568 | 422.0890413 | DALYs | DALYs (Disability-Adjusted Life Years) | 0 | Total change (desc) | 34 |
| Congo | Central Sub-Saharan Africa | 838.6280976 | 1647.981082 | 809.352984 | 428.6957982 | 37.80982387 | 342.847362 | DALYs | DALYs (Disability-Adjusted Life Years) | 0 | Total change (desc) | 35 |
| Sierra Leone | Western Sub-Saharan Africa | 1320.099687 | 2095.555156 | 775.4554686 | 409.2169846 | -116.9105543 | 483.1490383 | DALYs | DALYs (Disability-Adjusted Life Years) | 0 | Total change (desc) | 36 |
| Liberia | Western Sub-Saharan Africa | 791.0722924 | 1526.661424 | 735.5891311 | 401.6563463 | 106.3826895 | 227.5500953 | DALYs | DALYs (Disability-Adjusted Life Years) | 0 | Total change (desc) | 37 |
| Mauritania | Western Sub-Saharan Africa | 732.9054138 | 1288.359354 | 555.4539405 | 302.3866188 | 76.72943449 | 176.3378872 | DALYs | DALYs (Disability-Adjusted Life Years) | 0 | Total change (desc) | 38 |
| Benin | Western Sub-Saharan Africa | 731.4276219 | 1234.822162 | 503.3945398 | 391.4741832 | 71.78004386 | 40.1403127 | DALYs | DALYs (Disability-Adjusted Life Years) | 0 | Total change (desc) | 39 |
| Central African Republic | Central Sub-Saharan Africa | 502.697471 | 917.0736606 | 414.3761896 | 210.874571 | 44.31773882 | 159.1838798 | DALYs | DALYs (Disability-Adjusted Life Years) | 5.68434E-14 | Total change (desc) | 40 |
| Gabon | Central Sub-Saharan Africa | 416.240702 | 792.5210261 | 376.2803241 | 141.2064007 | 61.78795912 | 173.2859643 | DALYs | DALYs (Disability-Adjusted Life Years) | 5.68434E-14 | Total change (desc) | 41 |
| Equatorial Guinea | Central Sub-Saharan Africa | 235.18568 | 497.1123977 | 261.9267177 | 158.8483501 | 14.7773736 | 88.30099396 | DALYs | DALYs (Disability-Adjusted Life Years) | 0 | Total change (desc) | 42 |
| Djibouti | Eastern Sub-Saharan Africa | 181.097073 | 438.2540294 | 257.1569564 | 123.0068633 | 61.02499954 | 73.12509357 | DALYs | DALYs (Disability-Adjusted Life Years) | 0 | Total change (desc) | 43 |
| Namibia | Southern Sub-Saharan Africa | 272.6902326 | 476.3574813 | 203.6672487 | 125.0235597 | 33.3717731 | 45.2719159 | DALYs | DALYs (Disability-Adjusted Life Years) | 0 | Total change (desc) | 44 |
| Mauritius | Eastern Sub-Saharan Africa | 617.9256364 | 821.4724712 | 203.5468348 | 0.113582672 | 262.6084163 | -59.17516416 | DALYs | DALYs (Disability-Adjusted Life Years) | -2.84217E-14 | Total change (desc) | 45 |
| Guinea-Bissau | Western Sub-Saharan Africa | 197.6309353 | 366.7547914 | 169.1238561 | 86.44693821 | 7.730788943 | 74.94612897 | DALYs | DALYs (Disability-Adjusted Life Years) | 2.84217E-14 | Total change (desc) | 46 |
| Lesotho | Southern Sub-Saharan Africa | 265.1076864 | 426.4472556 | 161.3395692 | 12.93966843 | 48.03774145 | 100.3621593 | DALYs | DALYs (Disability-Adjusted Life Years) | 0 | Total change (desc) | 47 |
| Botswana | Southern Sub-Saharan Africa | 92.12720203 | 199.2178454 | 107.0906434 | 25.50990604 | 32.40795857 | 49.1727788 | DALYs | DALYs (Disability-Adjusted Life Years) | 0 | Total change (desc) | 48 |
| Gambia | Western Sub-Saharan Africa | 99.98738577 | 198.3342637 | 98.34687796 | 49.28541583 | 5.581222349 | 43.48023979 | DALYs | DALYs (Disability-Adjusted Life Years) | -1.42109E-14 | Total change (desc) | 49 |
| Sao Tome and Principe | Central Sub-Saharan Africa | 86.10837118 | 145.4735132 | 59.36514203 | 25.27344737 | 18.7536657 | 15.33802896 | DALYs | DALYs (Disability-Adjusted Life Years) | 0 | Total change (desc) | 50 |
| Cabo Verde | Western Sub-Saharan Africa | 125.5374576 | 184.3120081 | 58.77455047 | -5.851683175 | 37.94323608 | 26.68299757 | DALYs | DALYs (Disability-Adjusted Life Years) | 0 | Total change (desc) | 51 |
| Comoros | Eastern Sub-Saharan Africa | 85.50163114 | 138.4765583 | 52.9749272 | 30.30214344 | 12.22671891 | 10.44606485 | DALYs | DALYs (Disability-Adjusted Life Years) | 0 | Total change (desc) | 52 |
| Seychelles | Eastern Sub-Saharan Africa | 78.42420746 | 105.2029145 | 26.778707 | 10.90429137 | 24.19157409 | -8.317158461 | DALYs | DALYs (Disability-Adjusted Life Years) | 0 | Total change (desc) | 53 |
| Eswatini | Southern Sub-Saharan Africa | 125.8868409 | 152.0740192 | 26.18717826 | 12.06315769 | 32.48519318 | -18.3611726 | DALYs | DALYs (Disability-Adjusted Life Years) | 0 | Total change (desc) | 54 |
| Ethiopia | Eastern Sub-Saharan Africa | 16468.6494 | 38553.59226 | 22084.94286 | 7479.634204 | 3406.583381 | 11198.72528 | DALYs | DALYs (Disability-Adjusted Life Years) | 0 | Epidemiology (desc) | 1 |
| Democratic Republic of the Congo | Central Sub-Saharan Africa | 9982.211882 | 22415.98452 | 12433.77264 | 5401.259885 | 1031.717578 | 6000.795172 | DALYs | DALYs (Disability-Adjusted Life Years) | -1.81899E-12 | Epidemiology (desc) | 2 |
| Malawi | Eastern Sub-Saharan Africa | 7415.207737 | 15338.10604 | 7922.898308 | 3835.705021 | 888.7298494 | 3198.463438 | DALYs | DALYs (Disability-Adjusted Life Years) | 0 | Epidemiology (desc) | 3 |
| Ghana | Western Sub-Saharan Africa | 6123.092433 | 12463.68958 | 6340.597146 | 2413.472269 | 931.2279969 | 2995.896881 | DALYs | DALYs (Disability-Adjusted Life Years) | 0 | Epidemiology (desc) | 4 |
| Nigeria | Western Sub-Saharan Africa | 14463.239 | 24391.81334 | 9928.574342 | 7054.925951 | 463.7195619 | 2409.928828 | DALYs | DALYs (Disability-Adjusted Life Years) | 0 | Epidemiology (desc) | 5 |
| Morocco | North Africa | 5954.883097 | 12413.81661 | 6458.933518 | 827.8527404 | 3259.518177 | 2371.5626 | DALYs | DALYs (Disability-Adjusted Life Years) | 0 | Epidemiology (desc) | 6 |
| Mali | Western Sub-Saharan Africa | 7216.496164 | 13382.1519 | 6165.655735 | 4103.094345 | 140.8849969 | 1921.676394 | DALYs | DALYs (Disability-Adjusted Life Years) | 0 | Epidemiology (desc) | 7 |
| Côte d'Ivoire | Western Sub-Saharan Africa | 2312.361298 | 6163.142699 | 3850.781401 | 1866.46353 | 108.8594736 | 1875.458398 | DALYs | DALYs (Disability-Adjusted Life Years) | 0 | Epidemiology (desc) | 8 |
| Algeria | North Africa | 7861.496312 | 14835.42164 | 6973.925329 | 2225.242362 | 2893.756512 | 1854.926454 | DALYs | DALYs (Disability-Adjusted Life Years) | 0 | Epidemiology (desc) | 9 |
| Madagascar | Eastern Sub-Saharan Africa | 5301.291837 | 10228.28459 | 4926.992757 | 2652.074581 | 549.1577272 | 1725.760448 | DALYs | DALYs (Disability-Adjusted Life Years) | 0 | Epidemiology (desc) | 10 |
| Rwanda | Eastern Sub-Saharan Africa | 2911.496978 | 6726.404487 | 3814.907509 | 1298.907247 | 1182.242739 | 1333.757523 | DALYs | DALYs (Disability-Adjusted Life Years) | 0 | Epidemiology (desc) | 11 |
| Uganda | Eastern Sub-Saharan Africa | 5612.168992 | 10240.16815 | 4627.999163 | 2525.174844 | 948.6554191 | 1154.168899 | DALYs | DALYs (Disability-Adjusted Life Years) | 0 | Epidemiology (desc) | 12 |
| Cameroon | Central Sub-Saharan Africa | 4301.222783 | 8254.932131 | 3953.709349 | 2572.964132 | 233.1063527 | 1147.638864 | DALYs | DALYs (Disability-Adjusted Life Years) | 0 | Epidemiology (desc) | 13 |
| Tunisia | North Africa | 8455.38744 | 14088.82927 | 5633.441831 | 1111.740689 | 3396.842566 | 1124.858575 | DALYs | DALYs (Disability-Adjusted Life Years) | 0 | Epidemiology (desc) | 14 |
| Zambia | Eastern Sub-Saharan Africa | 3399.003624 | 7004.547015 | 3605.543392 | 2262.428124 | 317.2085369 | 1025.906731 | DALYs | DALYs (Disability-Adjusted Life Years) | 0 | Epidemiology (desc) | 15 |
| Sudan | North Africa | 8380.966471 | 13549.35553 | 5168.389055 | 2225.859312 | 2134.979566 | 807.5501774 | DALYs | DALYs (Disability-Adjusted Life Years) | 0 | Epidemiology (desc) | 16 |
| Burundi | Eastern Sub-Saharan Africa | 1543.613458 | 3203.068115 | 1659.454657 | 964.8890823 | 96.34714611 | 598.218429 | DALYs | DALYs (Disability-Adjusted Life Years) | -2.27374E-13 | Epidemiology (desc) | 17 |
| Angola | Central Sub-Saharan Africa | 3618.224072 | 7066.055622 | 3447.83155 | 2361.349449 | 512.1471917 | 574.3349087 | DALYs | DALYs (Disability-Adjusted Life Years) | 0 | Epidemiology (desc) | 18 |
| South Africa | Southern Sub-Saharan Africa | 17808.40739 | 27424.53015 | 9616.122751 | 5100.922262 | 3942.775619 | 572.4248698 | DALYs | DALYs (Disability-Adjusted Life Years) | 0 | Epidemiology (desc) | 19 |
| Senegal | Western Sub-Saharan Africa | 1866.492117 | 3440.282665 | 1573.790548 | 1020.556534 | 24.92628407 | 528.3077294 | DALYs | DALYs (Disability-Adjusted Life Years) | 0 | Epidemiology (desc) | 20 |
| Kenya | Eastern Sub-Saharan Africa | 3610.470243 | 6551.855393 | 2941.38515 | 1184.52625 | 1235.365899 | 521.4930007 | DALYs | DALYs (Disability-Adjusted Life Years) | 0 | Epidemiology (desc) | 21 |
| Sierra Leone | Western Sub-Saharan Africa | 1320.099687 | 2095.555156 | 775.4554686 | 409.2169846 | -116.9105543 | 483.1490383 | DALYs | DALYs (Disability-Adjusted Life Years) | 0 | Epidemiology (desc) | 22 |
| Eritrea | Eastern Sub-Saharan Africa | 1081.794227 | 2120.207098 | 1038.412871 | 387.6228846 | 186.1744332 | 464.6155532 | DALYs | DALYs (Disability-Adjusted Life Years) | 0 | Epidemiology (desc) | 23 |
| Burkina Faso | Western Sub-Saharan Africa | 2315.610136 | 3967.622079 | 1652.011943 | 1112.458116 | 89.91178474 | 449.642043 | DALYs | DALYs (Disability-Adjusted Life Years) | 0 | Epidemiology (desc) | 24 |
| Chad | Central Sub-Saharan Africa | 1804.794537 | 3166.772409 | 1361.977872 | 1186.475185 | -255.5169748 | 431.0196621 | DALYs | DALYs (Disability-Adjusted Life Years) | 0 | Epidemiology (desc) | 25 |
| South Sudan | Eastern Sub-Saharan Africa | 2149.588803 | 3371.529412 | 1221.940609 | 160.5873586 | 636.5702262 | 424.7830248 | DALYs | DALYs (Disability-Adjusted Life Years) | 0 | Epidemiology (desc) | 26 |
| Guinea | Western Sub-Saharan Africa | 1633.266789 | 2520.655897 | 887.3891084 | 714.9902239 | -249.6901568 | 422.0890413 | DALYs | DALYs (Disability-Adjusted Life Years) | 0 | Epidemiology (desc) | 27 |
| Niger | Western Sub-Saharan Africa | 1835.355376 | 3487.270286 | 1651.91491 | 1251.22093 | 5.282946164 | 395.4110333 | DALYs | DALYs (Disability-Adjusted Life Years) | 0 | Epidemiology (desc) | 28 |
| Congo | Central Sub-Saharan Africa | 838.6280976 | 1647.981082 | 809.352984 | 428.6957982 | 37.80982387 | 342.847362 | DALYs | DALYs (Disability-Adjusted Life Years) | 0 | Epidemiology (desc) | 29 |
| Somalia | Eastern Sub-Saharan Africa | 1155.237536 | 2243.810143 | 1088.572607 | 711.8802814 | 42.48837073 | 334.2039551 | DALYs | DALYs (Disability-Adjusted Life Years) | 0 | Epidemiology (desc) | 30 |
| Togo | Western Sub-Saharan Africa | 899.5612754 | 1901.975588 | 1002.414313 | 386.1035389 | 295.6184836 | 320.69229 | DALYs | DALYs (Disability-Adjusted Life Years) | -1.13687E-13 | Epidemiology (desc) | 31 |
| Liberia | Western Sub-Saharan Africa | 791.0722924 | 1526.661424 | 735.5891311 | 401.6563463 | 106.3826895 | 227.5500953 | DALYs | DALYs (Disability-Adjusted Life Years) | 0 | Epidemiology (desc) | 32 |
| Mauritania | Western Sub-Saharan Africa | 732.9054138 | 1288.359354 | 555.4539405 | 302.3866188 | 76.72943449 | 176.3378872 | DALYs | DALYs (Disability-Adjusted Life Years) | 0 | Epidemiology (desc) | 33 |
| Gabon | Central Sub-Saharan Africa | 416.240702 | 792.5210261 | 376.2803241 | 141.2064007 | 61.78795912 | 173.2859643 | DALYs | DALYs (Disability-Adjusted Life Years) | 5.68434E-14 | Epidemiology (desc) | 34 |
| Central African Republic | Central Sub-Saharan Africa | 502.697471 | 917.0736606 | 414.3761896 | 210.874571 | 44.31773882 | 159.1838798 | DALYs | DALYs (Disability-Adjusted Life Years) | 5.68434E-14 | Epidemiology (desc) | 35 |
| Mozambique | Eastern Sub-Saharan Africa | 4403.573833 | 6260.51078 | 1856.936947 | 1928.913936 | -180.3201093 | 108.3431199 | DALYs | DALYs (Disability-Adjusted Life Years) | -2.27374E-13 | Epidemiology (desc) | 36 |
| Lesotho | Southern Sub-Saharan Africa | 265.1076864 | 426.4472556 | 161.3395692 | 12.93966843 | 48.03774145 | 100.3621593 | DALYs | DALYs (Disability-Adjusted Life Years) | 0 | Epidemiology (desc) | 37 |
| Equatorial Guinea | Central Sub-Saharan Africa | 235.18568 | 497.1123977 | 261.9267177 | 158.8483501 | 14.7773736 | 88.30099396 | DALYs | DALYs (Disability-Adjusted Life Years) | 0 | Epidemiology (desc) | 38 |
| Guinea-Bissau | Western Sub-Saharan Africa | 197.6309353 | 366.7547914 | 169.1238561 | 86.44693821 | 7.730788943 | 74.94612897 | DALYs | DALYs (Disability-Adjusted Life Years) | 2.84217E-14 | Epidemiology (desc) | 39 |
| Djibouti | Eastern Sub-Saharan Africa | 181.097073 | 438.2540294 | 257.1569564 | 123.0068633 | 61.02499954 | 73.12509357 | DALYs | DALYs (Disability-Adjusted Life Years) | 0 | Epidemiology (desc) | 40 |
| Botswana | Southern Sub-Saharan Africa | 92.12720203 | 199.2178454 | 107.0906434 | 25.50990604 | 32.40795857 | 49.1727788 | DALYs | DALYs (Disability-Adjusted Life Years) | 0 | Epidemiology (desc) | 41 |
| Namibia | Southern Sub-Saharan Africa | 272.6902326 | 476.3574813 | 203.6672487 | 125.0235597 | 33.3717731 | 45.2719159 | DALYs | DALYs (Disability-Adjusted Life Years) | 0 | Epidemiology (desc) | 42 |
| Gambia | Western Sub-Saharan Africa | 99.98738577 | 198.3342637 | 98.34687796 | 49.28541583 | 5.581222349 | 43.48023979 | DALYs | DALYs (Disability-Adjusted Life Years) | -1.42109E-14 | Epidemiology (desc) | 43 |
| Benin | Western Sub-Saharan Africa | 731.4276219 | 1234.822162 | 503.3945398 | 391.4741832 | 71.78004386 | 40.1403127 | DALYs | DALYs (Disability-Adjusted Life Years) | 0 | Epidemiology (desc) | 44 |
| Cabo Verde | Western Sub-Saharan Africa | 125.5374576 | 184.3120081 | 58.77455047 | -5.851683175 | 37.94323608 | 26.68299757 | DALYs | DALYs (Disability-Adjusted Life Years) | 0 | Epidemiology (desc) | 45 |
| Sao Tome and Principe | Central Sub-Saharan Africa | 86.10837118 | 145.4735132 | 59.36514203 | 25.27344737 | 18.7536657 | 15.33802896 | DALYs | DALYs (Disability-Adjusted Life Years) | 0 | Epidemiology (desc) | 46 |
| Comoros | Eastern Sub-Saharan Africa | 85.50163114 | 138.4765583 | 52.9749272 | 30.30214344 | 12.22671891 | 10.44606485 | DALYs | DALYs (Disability-Adjusted Life Years) | 0 | Epidemiology (desc) | 47 |
| Seychelles | Eastern Sub-Saharan Africa | 78.42420746 | 105.2029145 | 26.778707 | 10.90429137 | 24.19157409 | -8.317158461 | DALYs | DALYs (Disability-Adjusted Life Years) | 0 | Epidemiology (desc) | 48 |
| Eswatini | Southern Sub-Saharan Africa | 125.8868409 | 152.0740192 | 26.18717826 | 12.06315769 | 32.48519318 | -18.3611726 | DALYs | DALYs (Disability-Adjusted Life Years) | 0 | Epidemiology (desc) | 49 |
| Mauritius | Eastern Sub-Saharan Africa | 617.9256364 | 821.4724712 | 203.5468348 | 0.113582672 | 262.6084163 | -59.17516416 | DALYs | DALYs (Disability-Adjusted Life Years) | -2.84217E-14 | Epidemiology (desc) | 50 |
| Zimbabwe | Southern Sub-Saharan Africa | 4647.927977 | 6428.346121 | 1780.418144 | 1000.03252 | 1060.606998 | -280.2213738 | DALYs | DALYs (Disability-Adjusted Life Years) | 0 | Epidemiology (desc) | 51 |
| Libya | North Africa | 4020.257513 | 6332.613919 | 2312.356406 | 863.0277573 | 1809.254918 | -359.9262701 | DALYs | DALYs (Disability-Adjusted Life Years) | 0 | Epidemiology (desc) | 52 |
| United Republic of Tanzania | Eastern Sub-Saharan Africa | 11037.80565 | 16162.3224 | 5124.516747 | 5096.211073 | 947.2228104 | -918.917137 | DALYs | DALYs (Disability-Adjusted Life Years) | 9.09495E-13 | Epidemiology (desc) | 53 |
| Egypt | North Africa | 43026.32619 | 66393.86757 | 23367.54138 | 14067.67307 | 10626.47184 | -1326.603531 | DALYs | DALYs (Disability-Adjusted Life Years) | 0 | Epidemiology (desc) | 54 |
| Egypt | North Africa | 43026.32619 | 66393.86757 | 23367.54138 | 14067.67307 | 10626.47184 | -1326.603531 | DALYs | DALYs (Disability-Adjusted Life Years) | 0 | Aging/Structure (desc) | 1 |
| South Africa | Southern Sub-Saharan Africa | 17808.40739 | 27424.53015 | 9616.122751 | 5100.922262 | 3942.775619 | 572.4248698 | DALYs | DALYs (Disability-Adjusted Life Years) | 0 | Aging/Structure (desc) | 2 |
| Ethiopia | Eastern Sub-Saharan Africa | 16468.6494 | 38553.59226 | 22084.94286 | 7479.634204 | 3406.583381 | 11198.72528 | DALYs | DALYs (Disability-Adjusted Life Years) | 0 | Aging/Structure (desc) | 3 |
| Tunisia | North Africa | 8455.38744 | 14088.82927 | 5633.441831 | 1111.740689 | 3396.842566 | 1124.858575 | DALYs | DALYs (Disability-Adjusted Life Years) | 0 | Aging/Structure (desc) | 4 |
| Morocco | North Africa | 5954.883097 | 12413.81661 | 6458.933518 | 827.8527404 | 3259.518177 | 2371.5626 | DALYs | DALYs (Disability-Adjusted Life Years) | 0 | Aging/Structure (desc) | 5 |
| Algeria | North Africa | 7861.496312 | 14835.42164 | 6973.925329 | 2225.242362 | 2893.756512 | 1854.926454 | DALYs | DALYs (Disability-Adjusted Life Years) | 0 | Aging/Structure (desc) | 6 |
| Sudan | North Africa | 8380.966471 | 13549.35553 | 5168.389055 | 2225.859312 | 2134.979566 | 807.5501774 | DALYs | DALYs (Disability-Adjusted Life Years) | 0 | Aging/Structure (desc) | 7 |
| Libya | North Africa | 4020.257513 | 6332.613919 | 2312.356406 | 863.0277573 | 1809.254918 | -359.9262701 | DALYs | DALYs (Disability-Adjusted Life Years) | 0 | Aging/Structure (desc) | 8 |
| Kenya | Eastern Sub-Saharan Africa | 3610.470243 | 6551.855393 | 2941.38515 | 1184.52625 | 1235.365899 | 521.4930007 | DALYs | DALYs (Disability-Adjusted Life Years) | 0 | Aging/Structure (desc) | 9 |
| Rwanda | Eastern Sub-Saharan Africa | 2911.496978 | 6726.404487 | 3814.907509 | 1298.907247 | 1182.242739 | 1333.757523 | DALYs | DALYs (Disability-Adjusted Life Years) | 0 | Aging/Structure (desc) | 10 |
| Zimbabwe | Southern Sub-Saharan Africa | 4647.927977 | 6428.346121 | 1780.418144 | 1000.03252 | 1060.606998 | -280.2213738 | DALYs | DALYs (Disability-Adjusted Life Years) | 0 | Aging/Structure (desc) | 11 |
| Democratic Republic of the Congo | Central Sub-Saharan Africa | 9982.211882 | 22415.98452 | 12433.77264 | 5401.259885 | 1031.717578 | 6000.795172 | DALYs | DALYs (Disability-Adjusted Life Years) | -1.81899E-12 | Aging/Structure (desc) | 12 |
| Uganda | Eastern Sub-Saharan Africa | 5612.168992 | 10240.16815 | 4627.999163 | 2525.174844 | 948.6554191 | 1154.168899 | DALYs | DALYs (Disability-Adjusted Life Years) | 0 | Aging/Structure (desc) | 13 |
| United Republic of Tanzania | Eastern Sub-Saharan Africa | 11037.80565 | 16162.3224 | 5124.516747 | 5096.211073 | 947.2228104 | -918.917137 | DALYs | DALYs (Disability-Adjusted Life Years) | 9.09495E-13 | Aging/Structure (desc) | 14 |
| Ghana | Western Sub-Saharan Africa | 6123.092433 | 12463.68958 | 6340.597146 | 2413.472269 | 931.2279969 | 2995.896881 | DALYs | DALYs (Disability-Adjusted Life Years) | 0 | Aging/Structure (desc) | 15 |
| Malawi | Eastern Sub-Saharan Africa | 7415.207737 | 15338.10604 | 7922.898308 | 3835.705021 | 888.7298494 | 3198.463438 | DALYs | DALYs (Disability-Adjusted Life Years) | 0 | Aging/Structure (desc) | 16 |
| South Sudan | Eastern Sub-Saharan Africa | 2149.588803 | 3371.529412 | 1221.940609 | 160.5873586 | 636.5702262 | 424.7830248 | DALYs | DALYs (Disability-Adjusted Life Years) | 0 | Aging/Structure (desc) | 17 |
| Madagascar | Eastern Sub-Saharan Africa | 5301.291837 | 10228.28459 | 4926.992757 | 2652.074581 | 549.1577272 | 1725.760448 | DALYs | DALYs (Disability-Adjusted Life Years) | 0 | Aging/Structure (desc) | 18 |
| Angola | Central Sub-Saharan Africa | 3618.224072 | 7066.055622 | 3447.83155 | 2361.349449 | 512.1471917 | 574.3349087 | DALYs | DALYs (Disability-Adjusted Life Years) | 0 | Aging/Structure (desc) | 19 |
| Nigeria | Western Sub-Saharan Africa | 14463.239 | 24391.81334 | 9928.574342 | 7054.925951 | 463.7195619 | 2409.928828 | DALYs | DALYs (Disability-Adjusted Life Years) | 0 | Aging/Structure (desc) | 20 |
| Zambia | Eastern Sub-Saharan Africa | 3399.003624 | 7004.547015 | 3605.543392 | 2262.428124 | 317.2085369 | 1025.906731 | DALYs | DALYs (Disability-Adjusted Life Years) | 0 | Aging/Structure (desc) | 21 |
| Togo | Western Sub-Saharan Africa | 899.5612754 | 1901.975588 | 1002.414313 | 386.1035389 | 295.6184836 | 320.69229 | DALYs | DALYs (Disability-Adjusted Life Years) | -1.13687E-13 | Aging/Structure (desc) | 22 |
| Mauritius | Eastern Sub-Saharan Africa | 617.9256364 | 821.4724712 | 203.5468348 | 0.113582672 | 262.6084163 | -59.17516416 | DALYs | DALYs (Disability-Adjusted Life Years) | -2.84217E-14 | Aging/Structure (desc) | 23 |
| Cameroon | Central Sub-Saharan Africa | 4301.222783 | 8254.932131 | 3953.709349 | 2572.964132 | 233.1063527 | 1147.638864 | DALYs | DALYs (Disability-Adjusted Life Years) | 0 | Aging/Structure (desc) | 24 |
| Eritrea | Eastern Sub-Saharan Africa | 1081.794227 | 2120.207098 | 1038.412871 | 387.6228846 | 186.1744332 | 464.6155532 | DALYs | DALYs (Disability-Adjusted Life Years) | 0 | Aging/Structure (desc) | 25 |
| Mali | Western Sub-Saharan Africa | 7216.496164 | 13382.1519 | 6165.655735 | 4103.094345 | 140.8849969 | 1921.676394 | DALYs | DALYs (Disability-Adjusted Life Years) | 0 | Aging/Structure (desc) | 26 |
| Côte d'Ivoire | Western Sub-Saharan Africa | 2312.361298 | 6163.142699 | 3850.781401 | 1866.46353 | 108.8594736 | 1875.458398 | DALYs | DALYs (Disability-Adjusted Life Years) | 0 | Aging/Structure (desc) | 27 |
| Liberia | Western Sub-Saharan Africa | 791.0722924 | 1526.661424 | 735.5891311 | 401.6563463 | 106.3826895 | 227.5500953 | DALYs | DALYs (Disability-Adjusted Life Years) | 0 | Aging/Structure (desc) | 28 |
| Burundi | Eastern Sub-Saharan Africa | 1543.613458 | 3203.068115 | 1659.454657 | 964.8890823 | 96.34714611 | 598.218429 | DALYs | DALYs (Disability-Adjusted Life Years) | -2.27374E-13 | Aging/Structure (desc) | 29 |
| Burkina Faso | Western Sub-Saharan Africa | 2315.610136 | 3967.622079 | 1652.011943 | 1112.458116 | 89.91178474 | 449.642043 | DALYs | DALYs (Disability-Adjusted Life Years) | 0 | Aging/Structure (desc) | 30 |
| Mauritania | Western Sub-Saharan Africa | 732.9054138 | 1288.359354 | 555.4539405 | 302.3866188 | 76.72943449 | 176.3378872 | DALYs | DALYs (Disability-Adjusted Life Years) | 0 | Aging/Structure (desc) | 31 |
| Benin | Western Sub-Saharan Africa | 731.4276219 | 1234.822162 | 503.3945398 | 391.4741832 | 71.78004386 | 40.1403127 | DALYs | DALYs (Disability-Adjusted Life Years) | 0 | Aging/Structure (desc) | 32 |
| Gabon | Central Sub-Saharan Africa | 416.240702 | 792.5210261 | 376.2803241 | 141.2064007 | 61.78795912 | 173.2859643 | DALYs | DALYs (Disability-Adjusted Life Years) | 5.68434E-14 | Aging/Structure (desc) | 33 |
| Djibouti | Eastern Sub-Saharan Africa | 181.097073 | 438.2540294 | 257.1569564 | 123.0068633 | 61.02499954 | 73.12509357 | DALYs | DALYs (Disability-Adjusted Life Years) | 0 | Aging/Structure (desc) | 34 |
| Lesotho | Southern Sub-Saharan Africa | 265.1076864 | 426.4472556 | 161.3395692 | 12.93966843 | 48.03774145 | 100.3621593 | DALYs | DALYs (Disability-Adjusted Life Years) | 0 | Aging/Structure (desc) | 35 |
| Central African Republic | Central Sub-Saharan Africa | 502.697471 | 917.0736606 | 414.3761896 | 210.874571 | 44.31773882 | 159.1838798 | DALYs | DALYs (Disability-Adjusted Life Years) | 5.68434E-14 | Aging/Structure (desc) | 36 |
| Somalia | Eastern Sub-Saharan Africa | 1155.237536 | 2243.810143 | 1088.572607 | 711.8802814 | 42.48837073 | 334.2039551 | DALYs | DALYs (Disability-Adjusted Life Years) | 0 | Aging/Structure (desc) | 37 |
| Cabo Verde | Western Sub-Saharan Africa | 125.5374576 | 184.3120081 | 58.77455047 | -5.851683175 | 37.94323608 | 26.68299757 | DALYs | DALYs (Disability-Adjusted Life Years) | 0 | Aging/Structure (desc) | 38 |
| Congo | Central Sub-Saharan Africa | 838.6280976 | 1647.981082 | 809.352984 | 428.6957982 | 37.80982387 | 342.847362 | DALYs | DALYs (Disability-Adjusted Life Years) | 0 | Aging/Structure (desc) | 39 |
| Namibia | Southern Sub-Saharan Africa | 272.6902326 | 476.3574813 | 203.6672487 | 125.0235597 | 33.3717731 | 45.2719159 | DALYs | DALYs (Disability-Adjusted Life Years) | 0 | Aging/Structure (desc) | 40 |
| Eswatini | Southern Sub-Saharan Africa | 125.8868409 | 152.0740192 | 26.18717826 | 12.06315769 | 32.48519318 | -18.3611726 | DALYs | DALYs (Disability-Adjusted Life Years) | 0 | Aging/Structure (desc) | 41 |
| Botswana | Southern Sub-Saharan Africa | 92.12720203 | 199.2178454 | 107.0906434 | 25.50990604 | 32.40795857 | 49.1727788 | DALYs | DALYs (Disability-Adjusted Life Years) | 0 | Aging/Structure (desc) | 42 |
| Senegal | Western Sub-Saharan Africa | 1866.492117 | 3440.282665 | 1573.790548 | 1020.556534 | 24.92628407 | 528.3077294 | DALYs | DALYs (Disability-Adjusted Life Years) | 0 | Aging/Structure (desc) | 43 |
| Seychelles | Eastern Sub-Saharan Africa | 78.42420746 | 105.2029145 | 26.778707 | 10.90429137 | 24.19157409 | -8.317158461 | DALYs | DALYs (Disability-Adjusted Life Years) | 0 | Aging/Structure (desc) | 44 |
| Sao Tome and Principe | Central Sub-Saharan Africa | 86.10837118 | 145.4735132 | 59.36514203 | 25.27344737 | 18.7536657 | 15.33802896 | DALYs | DALYs (Disability-Adjusted Life Years) | 0 | Aging/Structure (desc) | 45 |
| Equatorial Guinea | Central Sub-Saharan Africa | 235.18568 | 497.1123977 | 261.9267177 | 158.8483501 | 14.7773736 | 88.30099396 | DALYs | DALYs (Disability-Adjusted Life Years) | 0 | Aging/Structure (desc) | 46 |
| Comoros | Eastern Sub-Saharan Africa | 85.50163114 | 138.4765583 | 52.9749272 | 30.30214344 | 12.22671891 | 10.44606485 | DALYs | DALYs (Disability-Adjusted Life Years) | 0 | Aging/Structure (desc) | 47 |
| Guinea-Bissau | Western Sub-Saharan Africa | 197.6309353 | 366.7547914 | 169.1238561 | 86.44693821 | 7.730788943 | 74.94612897 | DALYs | DALYs (Disability-Adjusted Life Years) | 2.84217E-14 | Aging/Structure (desc) | 48 |
| Gambia | Western Sub-Saharan Africa | 99.98738577 | 198.3342637 | 98.34687796 | 49.28541583 | 5.581222349 | 43.48023979 | DALYs | DALYs (Disability-Adjusted Life Years) | -1.42109E-14 | Aging/Structure (desc) | 49 |
| Niger | Western Sub-Saharan Africa | 1835.355376 | 3487.270286 | 1651.91491 | 1251.22093 | 5.282946164 | 395.4110333 | DALYs | DALYs (Disability-Adjusted Life Years) | 0 | Aging/Structure (desc) | 50 |
| Sierra Leone | Western Sub-Saharan Africa | 1320.099687 | 2095.555156 | 775.4554686 | 409.2169846 | -116.9105543 | 483.1490383 | DALYs | DALYs (Disability-Adjusted Life Years) | 0 | Aging/Structure (desc) | 51 |
| Mozambique | Eastern Sub-Saharan Africa | 4403.573833 | 6260.51078 | 1856.936947 | 1928.913936 | -180.3201093 | 108.3431199 | DALYs | DALYs (Disability-Adjusted Life Years) | -2.27374E-13 | Aging/Structure (desc) | 52 |
| Guinea | Western Sub-Saharan Africa | 1633.266789 | 2520.655897 | 887.3891084 | 714.9902239 | -249.6901568 | 422.0890413 | DALYs | DALYs (Disability-Adjusted Life Years) | 0 | Aging/Structure (desc) | 53 |
| Chad | Central Sub-Saharan Africa | 1804.794537 | 3166.772409 | 1361.977872 | 1186.475185 | -255.5169748 | 431.0196621 | DALYs | DALYs (Disability-Adjusted Life Years) | 0 | Aging/Structure (desc) | 54 |
| Egypt | North Africa | 43026.32619 | 66393.86757 | 23367.54138 | 14067.67307 | 10626.47184 | -1326.603531 | DALYs | DALYs (Disability-Adjusted Life Years) | 0 | Population (desc) | 1 |
| Ethiopia | Eastern Sub-Saharan Africa | 16468.6494 | 38553.59226 | 22084.94286 | 7479.634204 | 3406.583381 | 11198.72528 | DALYs | DALYs (Disability-Adjusted Life Years) | 0 | Population (desc) | 2 |
| Nigeria | Western Sub-Saharan Africa | 14463.239 | 24391.81334 | 9928.574342 | 7054.925951 | 463.7195619 | 2409.928828 | DALYs | DALYs (Disability-Adjusted Life Years) | 0 | Population (desc) | 3 |
| Democratic Republic of the Congo | Central Sub-Saharan Africa | 9982.211882 | 22415.98452 | 12433.77264 | 5401.259885 | 1031.717578 | 6000.795172 | DALYs | DALYs (Disability-Adjusted Life Years) | -1.81899E-12 | Population (desc) | 4 |
| South Africa | Southern Sub-Saharan Africa | 17808.40739 | 27424.53015 | 9616.122751 | 5100.922262 | 3942.775619 | 572.4248698 | DALYs | DALYs (Disability-Adjusted Life Years) | 0 | Population (desc) | 5 |
| United Republic of Tanzania | Eastern Sub-Saharan Africa | 11037.80565 | 16162.3224 | 5124.516747 | 5096.211073 | 947.2228104 | -918.917137 | DALYs | DALYs (Disability-Adjusted Life Years) | 9.09495E-13 | Population (desc) | 6 |
| Mali | Western Sub-Saharan Africa | 7216.496164 | 13382.1519 | 6165.655735 | 4103.094345 | 140.8849969 | 1921.676394 | DALYs | DALYs (Disability-Adjusted Life Years) | 0 | Population (desc) | 7 |
| Malawi | Eastern Sub-Saharan Africa | 7415.207737 | 15338.10604 | 7922.898308 | 3835.705021 | 888.7298494 | 3198.463438 | DALYs | DALYs (Disability-Adjusted Life Years) | 0 | Population (desc) | 8 |
| Madagascar | Eastern Sub-Saharan Africa | 5301.291837 | 10228.28459 | 4926.992757 | 2652.074581 | 549.1577272 | 1725.760448 | DALYs | DALYs (Disability-Adjusted Life Years) | 0 | Population (desc) | 9 |
| Cameroon | Central Sub-Saharan Africa | 4301.222783 | 8254.932131 | 3953.709349 | 2572.964132 | 233.1063527 | 1147.638864 | DALYs | DALYs (Disability-Adjusted Life Years) | 0 | Population (desc) | 10 |
| Uganda | Eastern Sub-Saharan Africa | 5612.168992 | 10240.16815 | 4627.999163 | 2525.174844 | 948.6554191 | 1154.168899 | DALYs | DALYs (Disability-Adjusted Life Years) | 0 | Population (desc) | 11 |
| Ghana | Western Sub-Saharan Africa | 6123.092433 | 12463.68958 | 6340.597146 | 2413.472269 | 931.2279969 | 2995.896881 | DALYs | DALYs (Disability-Adjusted Life Years) | 0 | Population (desc) | 12 |
| Angola | Central Sub-Saharan Africa | 3618.224072 | 7066.055622 | 3447.83155 | 2361.349449 | 512.1471917 | 574.3349087 | DALYs | DALYs (Disability-Adjusted Life Years) | 0 | Population (desc) | 13 |
| Zambia | Eastern Sub-Saharan Africa | 3399.003624 | 7004.547015 | 3605.543392 | 2262.428124 | 317.2085369 | 1025.906731 | DALYs | DALYs (Disability-Adjusted Life Years) | 0 | Population (desc) | 14 |
| Sudan | North Africa | 8380.966471 | 13549.35553 | 5168.389055 | 2225.859312 | 2134.979566 | 807.5501774 | DALYs | DALYs (Disability-Adjusted Life Years) | 0 | Population (desc) | 15 |
| Algeria | North Africa | 7861.496312 | 14835.42164 | 6973.925329 | 2225.242362 | 2893.756512 | 1854.926454 | DALYs | DALYs (Disability-Adjusted Life Years) | 0 | Population (desc) | 16 |
| Mozambique | Eastern Sub-Saharan Africa | 4403.573833 | 6260.51078 | 1856.936947 | 1928.913936 | -180.3201093 | 108.3431199 | DALYs | DALYs (Disability-Adjusted Life Years) | -2.27374E-13 | Population (desc) | 17 |
| Côte d'Ivoire | Western Sub-Saharan Africa | 2312.361298 | 6163.142699 | 3850.781401 | 1866.46353 | 108.8594736 | 1875.458398 | DALYs | DALYs (Disability-Adjusted Life Years) | 0 | Population (desc) | 18 |
| Rwanda | Eastern Sub-Saharan Africa | 2911.496978 | 6726.404487 | 3814.907509 | 1298.907247 | 1182.242739 | 1333.757523 | DALYs | DALYs (Disability-Adjusted Life Years) | 0 | Population (desc) | 19 |
| Niger | Western Sub-Saharan Africa | 1835.355376 | 3487.270286 | 1651.91491 | 1251.22093 | 5.282946164 | 395.4110333 | DALYs | DALYs (Disability-Adjusted Life Years) | 0 | Population (desc) | 20 |
| Chad | Central Sub-Saharan Africa | 1804.794537 | 3166.772409 | 1361.977872 | 1186.475185 | -255.5169748 | 431.0196621 | DALYs | DALYs (Disability-Adjusted Life Years) | 0 | Population (desc) | 21 |
| Kenya | Eastern Sub-Saharan Africa | 3610.470243 | 6551.855393 | 2941.38515 | 1184.52625 | 1235.365899 | 521.4930007 | DALYs | DALYs (Disability-Adjusted Life Years) | 0 | Population (desc) | 22 |
| Burkina Faso | Western Sub-Saharan Africa | 2315.610136 | 3967.622079 | 1652.011943 | 1112.458116 | 89.91178474 | 449.642043 | DALYs | DALYs (Disability-Adjusted Life Years) | 0 | Population (desc) | 23 |
| Tunisia | North Africa | 8455.38744 | 14088.82927 | 5633.441831 | 1111.740689 | 3396.842566 | 1124.858575 | DALYs | DALYs (Disability-Adjusted Life Years) | 0 | Population (desc) | 24 |
| Senegal | Western Sub-Saharan Africa | 1866.492117 | 3440.282665 | 1573.790548 | 1020.556534 | 24.92628407 | 528.3077294 | DALYs | DALYs (Disability-Adjusted Life Years) | 0 | Population (desc) | 25 |
| Zimbabwe | Southern Sub-Saharan Africa | 4647.927977 | 6428.346121 | 1780.418144 | 1000.03252 | 1060.606998 | -280.2213738 | DALYs | DALYs (Disability-Adjusted Life Years) | 0 | Population (desc) | 26 |
| Burundi | Eastern Sub-Saharan Africa | 1543.613458 | 3203.068115 | 1659.454657 | 964.8890823 | 96.34714611 | 598.218429 | DALYs | DALYs (Disability-Adjusted Life Years) | -2.27374E-13 | Population (desc) | 27 |
| Libya | North Africa | 4020.257513 | 6332.613919 | 2312.356406 | 863.0277573 | 1809.254918 | -359.9262701 | DALYs | DALYs (Disability-Adjusted Life Years) | 0 | Population (desc) | 28 |
| Morocco | North Africa | 5954.883097 | 12413.81661 | 6458.933518 | 827.8527404 | 3259.518177 | 2371.5626 | DALYs | DALYs (Disability-Adjusted Life Years) | 0 | Population (desc) | 29 |
| Guinea | Western Sub-Saharan Africa | 1633.266789 | 2520.655897 | 887.3891084 | 714.9902239 | -249.6901568 | 422.0890413 | DALYs | DALYs (Disability-Adjusted Life Years) | 0 | Population (desc) | 30 |
| Somalia | Eastern Sub-Saharan Africa | 1155.237536 | 2243.810143 | 1088.572607 | 711.8802814 | 42.48837073 | 334.2039551 | DALYs | DALYs (Disability-Adjusted Life Years) | 0 | Population (desc) | 31 |
| Congo | Central Sub-Saharan Africa | 838.6280976 | 1647.981082 | 809.352984 | 428.6957982 | 37.80982387 | 342.847362 | DALYs | DALYs (Disability-Adjusted Life Years) | 0 | Population (desc) | 32 |
| Sierra Leone | Western Sub-Saharan Africa | 1320.099687 | 2095.555156 | 775.4554686 | 409.2169846 | -116.9105543 | 483.1490383 | DALYs | DALYs (Disability-Adjusted Life Years) | 0 | Population (desc) | 33 |
| Liberia | Western Sub-Saharan Africa | 791.0722924 | 1526.661424 | 735.5891311 | 401.6563463 | 106.3826895 | 227.5500953 | DALYs | DALYs (Disability-Adjusted Life Years) | 0 | Population (desc) | 34 |
| Benin | Western Sub-Saharan Africa | 731.4276219 | 1234.822162 | 503.3945398 | 391.4741832 | 71.78004386 | 40.1403127 | DALYs | DALYs (Disability-Adjusted Life Years) | 0 | Population (desc) | 35 |
| Eritrea | Eastern Sub-Saharan Africa | 1081.794227 | 2120.207098 | 1038.412871 | 387.6228846 | 186.1744332 | 464.6155532 | DALYs | DALYs (Disability-Adjusted Life Years) | 0 | Population (desc) | 36 |
| Togo | Western Sub-Saharan Africa | 899.5612754 | 1901.975588 | 1002.414313 | 386.1035389 | 295.6184836 | 320.69229 | DALYs | DALYs (Disability-Adjusted Life Years) | -1.13687E-13 | Population (desc) | 37 |
| Mauritania | Western Sub-Saharan Africa | 732.9054138 | 1288.359354 | 555.4539405 | 302.3866188 | 76.72943449 | 176.3378872 | DALYs | DALYs (Disability-Adjusted Life Years) | 0 | Population (desc) | 38 |
| Central African Republic | Central Sub-Saharan Africa | 502.697471 | 917.0736606 | 414.3761896 | 210.874571 | 44.31773882 | 159.1838798 | DALYs | DALYs (Disability-Adjusted Life Years) | 5.68434E-14 | Population (desc) | 39 |
| South Sudan | Eastern Sub-Saharan Africa | 2149.588803 | 3371.529412 | 1221.940609 | 160.5873586 | 636.5702262 | 424.7830248 | DALYs | DALYs (Disability-Adjusted Life Years) | 0 | Population (desc) | 40 |
| Equatorial Guinea | Central Sub-Saharan Africa | 235.18568 | 497.1123977 | 261.9267177 | 158.8483501 | 14.7773736 | 88.30099396 | DALYs | DALYs (Disability-Adjusted Life Years) | 0 | Population (desc) | 41 |
| Gabon | Central Sub-Saharan Africa | 416.240702 | 792.5210261 | 376.2803241 | 141.2064007 | 61.78795912 | 173.2859643 | DALYs | DALYs (Disability-Adjusted Life Years) | 5.68434E-14 | Population (desc) | 42 |
| Namibia | Southern Sub-Saharan Africa | 272.6902326 | 476.3574813 | 203.6672487 | 125.0235597 | 33.3717731 | 45.2719159 | DALYs | DALYs (Disability-Adjusted Life Years) | 0 | Population (desc) | 43 |
| Djibouti | Eastern Sub-Saharan Africa | 181.097073 | 438.2540294 | 257.1569564 | 123.0068633 | 61.02499954 | 73.12509357 | DALYs | DALYs (Disability-Adjusted Life Years) | 0 | Population (desc) | 44 |
| Guinea-Bissau | Western Sub-Saharan Africa | 197.6309353 | 366.7547914 | 169.1238561 | 86.44693821 | 7.730788943 | 74.94612897 | DALYs | DALYs (Disability-Adjusted Life Years) | 2.84217E-14 | Population (desc) | 45 |
| Gambia | Western Sub-Saharan Africa | 99.98738577 | 198.3342637 | 98.34687796 | 49.28541583 | 5.581222349 | 43.48023979 | DALYs | DALYs (Disability-Adjusted Life Years) | -1.42109E-14 | Population (desc) | 46 |
| Comoros | Eastern Sub-Saharan Africa | 85.50163114 | 138.4765583 | 52.9749272 | 30.30214344 | 12.22671891 | 10.44606485 | DALYs | DALYs (Disability-Adjusted Life Years) | 0 | Population (desc) | 47 |
| Botswana | Southern Sub-Saharan Africa | 92.12720203 | 199.2178454 | 107.0906434 | 25.50990604 | 32.40795857 | 49.1727788 | DALYs | DALYs (Disability-Adjusted Life Years) | 0 | Population (desc) | 48 |
| Sao Tome and Principe | Central Sub-Saharan Africa | 86.10837118 | 145.4735132 | 59.36514203 | 25.27344737 | 18.7536657 | 15.33802896 | DALYs | DALYs (Disability-Adjusted Life Years) | 0 | Population (desc) | 49 |
| Lesotho | Southern Sub-Saharan Africa | 265.1076864 | 426.4472556 | 161.3395692 | 12.93966843 | 48.03774145 | 100.3621593 | DALYs | DALYs (Disability-Adjusted Life Years) | 0 | Population (desc) | 50 |
| Eswatini | Southern Sub-Saharan Africa | 125.8868409 | 152.0740192 | 26.18717826 | 12.06315769 | 32.48519318 | -18.3611726 | DALYs | DALYs (Disability-Adjusted Life Years) | 0 | Population (desc) | 51 |
| Seychelles | Eastern Sub-Saharan Africa | 78.42420746 | 105.2029145 | 26.778707 | 10.90429137 | 24.19157409 | -8.317158461 | DALYs | DALYs (Disability-Adjusted Life Years) | 0 | Population (desc) | 52 |
| Mauritius | Eastern Sub-Saharan Africa | 617.9256364 | 821.4724712 | 203.5468348 | 0.113582672 | 262.6084163 | -59.17516416 | DALYs | DALYs (Disability-Adjusted Life Years) | -2.84217E-14 | Population (desc) | 53 |
| Cabo Verde | Western Sub-Saharan Africa | 125.5374576 | 184.3120081 | 58.77455047 | -5.851683175 | 37.94323608 | 26.68299757 | DALYs | DALYs (Disability-Adjusted Life Years) | 0 | Population (desc) | 54 |
