# Supplementary material for: Bladder cancer in Africa, 2010–2023: burden, geographic heterogeneity, demographic drivers, pandemic-era temporal changes, and projections to 2030
Source: Infect Agent Cancer. 2026 Jul 23;21:69. doi: 10.1186/s13027-026-00782-3 (PMC13681883; doi:10.1186/s13027-026-00782-3)
Supplement: Supplementary file 5 [file 13027_2026_782_MOESM5_ESM.docx]

Data_Deaths

| **year** | **val** | **Expected** | **Lower_95** | **Upper_95** | **Period** | **Gap** | **ymin_gap** | **ymax_gap** | **gap_type** |
| --- | --- | --- | --- | --- | --- | --- | --- | --- | --- |
| 2010 | 2.21 | 2.21 |  |  | Pre-pandemic (fit) | 0 | 2.207352238 | 2.208134351 |  |
| 2011 | 2.24 | 2.24 |  |  | Pre-pandemic (fit) | 0 | 2.240036655 | 2.242346652 |  |
| 2012 | 2.26 | 2.27 |  |  | Pre-pandemic (fit) | -0.01 | 2.262512862 | 2.272399871 |  |
| 2013 | 2.26 | 2.29 |  |  | Pre-pandemic (fit) | -0.02 | 2.262694316 | 2.285214592 |  |
| 2014 | 2.28 | 2.26 |  |  | Pre-pandemic (fit) | 0.01 | 2.262875784 | 2.276823396 |  |
| 2015 | 2.3 | 2.29 |  |  | Pre-pandemic (fit) | 0.01 | 2.291040703 | 2.303912917 |  |
| 2016 | 2.3 | 2.33 |  |  | Pre-pandemic (fit) | -0.03 | 2.304407698 | 2.331324747 |  |
| 2017 | 2.29 | 2.3 |  |  | Pre-pandemic (fit) | -0.01 | 2.293257546 | 2.304902586 |  |
| 2018 | 2.28 | 2.28 |  |  | Pre-pandemic (fit) | 0 | 2.282161346 | 2.284920083 |  |
| 2019 | 2.28 | 2.28 |  |  | Pre-pandemic (fit) | 0 | 2.276612931 | 2.277932172 |  |
| 2020 | 2.31 | 2.27 | 2.24 | 2.3 | Pandemic period | 0.04 | 2.270965631 | 2.307974296 | Excess burden |
| 2021 | 2.44 | 2.26 | 2.2 | 2.33 | Pandemic period | 0.18 | 2.264020396 | 2.444752928 | Excess burden |
| 2022 | 2.51 | 2.26 | 2.15 | 2.37 | Pandemic period | 0.25 | 2.257096402 | 2.510258086 | Excess burden |
| 2023 | 2.57 | 2.25 | 2.1 | 2.42 | Pandemic period | 0.32 | 2.250193583 | 2.570897972 | Excess burden |

Data_DALYs

| **year** | **val** | **Expected** | **Lower_95** | **Upper_95** | **Period** | **Gap** | **ymin_gap** | **ymax_gap** | **gap_type** |
| --- | --- | --- | --- | --- | --- | --- | --- | --- | --- |
| 2010 | 48.11 | 48.03 |  |  | Pre-pandemic (fit) | 0.08 | 48.0296885 | 48.11296266 |  |
| 2011 | 48.79 | 49.05 |  |  | Pre-pandemic (fit) | -0.25 | 48.79311828 | 49.04581177 |  |
| 2012 | 49.24 | 49.48 |  |  | Pre-pandemic (fit) | -0.24 | 49.238799 | 49.48288901 |  |
| 2013 | 49.18 | 49.69 |  |  | Pre-pandemic (fit) | -0.51 | 49.1828903 | 49.68855061 |  |
| 2014 | 49.53 | 49.13 |  |  | Pre-pandemic (fit) | 0.41 | 49.12704509 | 49.53262804 |  |
| 2015 | 50.08 | 49.88 |  |  | Pre-pandemic (fit) | 0.19 | 49.88485275 | 50.07797106 |  |
| 2016 | 50.04 | 50.63 |  |  | Pre-pandemic (fit) | -0.59 | 50.04329953 | 50.62931818 |  |
| 2017 | 49.75 | 50.01 |  |  | Pre-pandemic (fit) | -0.26 | 49.75102888 | 50.008652 |  |
| 2018 | 49.59 | 49.46 |  |  | Pre-pandemic (fit) | 0.13 | 49.4604652 | 49.58947573 |  |
| 2019 | 49.43 | 49.43 |  |  | Pre-pandemic (fit) | 0 | 49.42640568 | 49.42844718 |  |
| 2020 | 49.55 | 49.26 | 48.58 | 49.96 | Pandemic period | 0.28 | 49.26387187 | 49.54783089 | Excess burden |
| 2021 | 52.38 | 49.1 | 47.58 | 50.67 | Pandemic period | 3.28 | 49.10187254 | 52.38493046 | Excess burden |
| 2022 | 54.57 | 48.94 | 46.44 | 51.58 | Pandemic period | 5.63 | 48.94040592 | 54.57116866 | Excess burden |
| 2023 | 56.11 | 48.78 | 45.17 | 52.68 | Pandemic period | 7.33 | 48.77947027 | 56.11021837 | Excess burden |

Data_Incidence

| **year** | **val** | **Expected** | **Lower_95** | **Upper_95** | **Period** | **Gap** | **ymin_gap** | **ymax_gap** | **gap_type** |
| --- | --- | --- | --- | --- | --- | --- | --- | --- | --- |
| 2010 | 3.48 | 3.48 |  |  | Pre-pandemic (fit) | 0 | 3.479964304 | 3.484283516 |  |
| 2011 | 3.54 | 3.51 |  |  | Pre-pandemic (fit) | 0.03 | 3.511798474 | 3.544108368 |  |
| 2012 | 3.59 | 3.57 |  |  | Pre-pandemic (fit) | 0.02 | 3.572095755 | 3.590843347 |  |
| 2013 | 3.59 | 3.62 |  |  | Pre-pandemic (fit) | -0.02 | 3.59453672 | 3.619199795 |  |
| 2014 | 3.63 | 3.62 |  |  | Pre-pandemic (fit) | 0.01 | 3.622922334 | 3.634480564 |  |
| 2015 | 3.7 | 3.66 |  |  | Pre-pandemic (fit) | 0.03 | 3.66318161 | 3.696044369 |  |
| 2016 | 3.72 | 3.73 |  |  | Pre-pandemic (fit) | -0.01 | 3.71699801 | 3.725231576 |  |
| 2017 | 3.72 | 3.75 |  |  | Pre-pandemic (fit) | -0.03 | 3.719760179 | 3.746350685 |  |
| 2018 | 3.73 | 3.75 |  |  | Pre-pandemic (fit) | -0.02 | 3.728647293 | 3.749134667 |  |
| 2019 | 3.74 | 3.76 |  |  | Pre-pandemic (fit) | -0.02 | 3.739886149 | 3.758091961 |  |
| 2020 | 3.78 | 3.77 | 3.72 | 3.82 | Pandemic period | 0.01 | 3.769419569 | 3.782152153 | Excess burden |
| 2021 | 4.02 | 3.8 | 3.73 | 3.87 | Pandemic period | 0.22 | 3.799186211 | 4.016370542 | Excess burden |
| 2022 | 4.22 | 3.83 | 3.74 | 3.92 | Pandemic period | 0.39 | 3.829187917 | 4.224032474 | Excess burden |
| 2023 | 4.38 | 3.86 | 3.76 | 3.96 | Pandemic period | 0.52 | 3.859426542 | 4.377133991 | Excess burden |

Data_Prevalence

| **year** | **val** | **Expected** | **Lower_95** | **Upper_95** | **Period** | **Gap** | **ymin_gap** | **ymax_gap** | **gap_type** |
| --- | --- | --- | --- | --- | --- | --- | --- | --- | --- |
| 2010 | 13.91 | 13.87 |  |  | Pre-pandemic (fit) | 0.04 | 13.87108561 | 13.90747828 |  |
| 2011 | 14.16 | 14.08 |  |  | Pre-pandemic (fit) | 0.09 | 14.07854653 | 14.16443027 |  |
| 2012 | 14.4 | 14.34 |  |  | Pre-pandemic (fit) | 0.06 | 14.33865914 | 14.39704347 |  |
| 2013 | 14.47 | 14.57 |  |  | Pre-pandemic (fit) | -0.11 | 14.46686668 | 14.5741336 |  |
| 2014 | 14.7 | 14.64 |  |  | Pre-pandemic (fit) | 0.06 | 14.64481566 | 14.70485838 |  |
| 2015 | 15 | 14.89 |  |  | Pre-pandemic (fit) | 0.11 | 14.88573477 | 14.99704342 |  |
| 2016 | 15.18 | 15.18 |  |  | Pre-pandemic (fit) | 0 | 15.17763485 | 15.18151382 |  |
| 2017 | 15.3 | 15.36 |  |  | Pre-pandemic (fit) | -0.06 | 15.30162917 | 15.3643266 |  |
| 2018 | 15.41 | 15.49 |  |  | Pre-pandemic (fit) | -0.08 | 15.41334127 | 15.4898461 |  |
| 2019 | 15.53 | 15.6 |  |  | Pre-pandemic (fit) | -0.08 | 15.52505911 | 15.60293231 |  |
| 2020 | 15.08 | 15.72 | 15.54 | 15.89 | Pandemic period | -0.63 | 15.08310473 | 15.71602433 | Observed below expected |
| 2021 | 15.27 | 15.91 | 15.66 | 16.16 | Pandemic period | -0.64 | 15.27018772 | 15.90933851 | Observed below expected |
| 2022 | 17.38 | 16.11 | 15.8 | 16.42 | Pandemic period | 1.28 | 16.10503055 | 17.3801465 | Excess burden |
| 2023 | 18.26 | 16.3 | 15.95 | 16.67 | Pandemic period | 1.96 | 16.30312968 | 18.26090758 | Excess burden |
