# Supplementary material for: Bladder cancer in Africa, 2010–2023: burden, geographic heterogeneity, demographic drivers, pandemic-era temporal changes, and projections to 2030
Source: Infect Agent Cancer. 2026 Jul 23;21:69. doi: 10.1186/s13027-026-00782-3 (PMC13681883; doi:10.1186/s13027-026-00782-3)
Supplement: Supplementary file 6 [file 13027_2026_782_MOESM6_ESM.docx]

| **Measure** | **Region** | **Country** | **Baseline (2023)** | **Projected (2030)** | **Absolute Change** | **Relative Change (%)** | **Trend** |
| --- | --- | --- | --- | --- | --- | --- | --- |
| DALYs (Disability-Adjusted Life Years) | 1 | Libya | 108.97 | 104.82 | -4.15 | -3.81 | Decrease |
| DALYs (Disability-Adjusted Life Years) | 1 | Tunisia | 90.09 | 87.43 | -2.66 | -2.95 | Decrease |
| DALYs (Disability-Adjusted Life Years) | 1 | Egypt | 88.21 | 84.31 | -3.9 | -4.42 | Decrease |
| DALYs (Disability-Adjusted Life Years) | 1 | Sudan | 59.08 | 61.76 | 2.68 | 4.54 | Increase |
| DALYs (Disability-Adjusted Life Years) | 1 | Algeria | 38.81 | 42.08 | 3.26 | 8.41 | Increase |
| DALYs (Disability-Adjusted Life Years) | 1 | Morocco | 34.35 | 39.5 | 5.15 | 15.01 | Increase |
| DALYs (Disability-Adjusted Life Years) | 2 | Sao Tome and Principe | 115.69 | 126.84 | 11.16 | 9.64 | Increase |
| DALYs (Disability-Adjusted Life Years) | 2 | Equatorial Guinea | 78.94 | 86.81 | 7.87 | 9.97 | Increase |
| DALYs (Disability-Adjusted Life Years) | 2 | Gabon | 59.75 | 67.69 | 7.94 | 13.29 | Increase |
| DALYs (Disability-Adjusted Life Years) | 2 | Congo | 58.31 | 66.95 | 8.64 | 14.82 | Increase |
| DALYs (Disability-Adjusted Life Years) | 2 | Democratic Republic of the Congo | 51.77 | 62.5 | 10.72 | 20.71 | Increase |
| DALYs (Disability-Adjusted Life Years) | 2 | Cameroon | 57.03 | 60.33 | 3.3 | 5.79 | Increase |
| DALYs (Disability-Adjusted Life Years) | 2 | Chad | 48.2 | 50.9 | 2.7 | 5.6 | Increase |
| DALYs (Disability-Adjusted Life Years) | 2 | Angola | 47.32 | 47.72 | 0.4 | 0.86 | Increase |
| DALYs (Disability-Adjusted Life Years) | 2 | Central African Republic | 31.32 | 33.8 | 2.48 | 7.91 | Increase |
| DALYs (Disability-Adjusted Life Years) | 3 | Malawi | 162.12 | 189.44 | 27.33 | 16.86 | Increase |
| DALYs (Disability-Adjusted Life Years) | 3 | Zambia | 87.85 | 98.55 | 10.71 | 12.19 | Increase |
| DALYs (Disability-Adjusted Life Years) | 3 | Rwanda | 78.89 | 86.84 | 7.95 | 10.07 | Increase |
| DALYs (Disability-Adjusted Life Years) | 3 | Ethiopia | 70.7 | 85.2 | 14.5 | 20.51 | Increase |
| DALYs (Disability-Adjusted Life Years) | 3 | Madagascar | 71.26 | 77.42 | 6.16 | 8.64 | Increase |
| DALYs (Disability-Adjusted Life Years) | 3 | South Sudan | 65.45 | 67.69 | 2.24 | 3.42 | Increase |
| DALYs (Disability-Adjusted Life Years) | 3 | Seychelles | 73.64 | 65.63 | -8.02 | -10.89 | Decrease |
| DALYs (Disability-Adjusted Life Years) | 3 | Burundi | 52.29 | 57.64 | 5.35 | 10.24 | Increase |
| DALYs (Disability-Adjusted Life Years) | 3 | Eritrea | 49.42 | 56.71 | 7.29 | 14.75 | Increase |
| DALYs (Disability-Adjusted Life Years) | 3 | Djibouti | 50.42 | 55.46 | 5.04 | 10 | Increase |
| DALYs (Disability-Adjusted Life Years) | 3 | Uganda | 52.51 | 54.8 | 2.28 | 4.35 | Increase |
| DALYs (Disability-Adjusted Life Years) | 3 | United Republic of Tanzania | 53.13 | 51.36 | -1.77 | -3.33 | Decrease |
| DALYs (Disability-Adjusted Life Years) | 3 | Mozambique | 45.35 | 46.57 | 1.22 | 2.69 | Increase |
| DALYs (Disability-Adjusted Life Years) | 3 | Mauritius | 41.43 | 37.77 | -3.66 | -8.84 | Decrease |
| DALYs (Disability-Adjusted Life Years) | 3 | Somalia | 23.74 | 27 | 3.27 | 13.76 | Increase |
| DALYs (Disability-Adjusted Life Years) | 3 | Comoros | 23.84 | 25.72 | 1.88 | 7.88 | Increase |
| DALYs (Disability-Adjusted Life Years) | 3 | Kenya | 23.23 | 25.31 | 2.08 | 8.97 | Increase |
| DALYs (Disability-Adjusted Life Years) | 4 | Zimbabwe | 73.85 | 74.71 | 0.86 | 1.17 | Increase |
| DALYs (Disability-Adjusted Life Years) | 4 | South Africa | 49.01 | 46.6 | -2.41 | -4.93 | Decrease |
| DALYs (Disability-Adjusted Life Years) | 4 | Lesotho | 29.85 | 32.8 | 2.94 | 9.86 | Increase |
| DALYs (Disability-Adjusted Life Years) | 4 | Namibia | 25.07 | 26.8 | 1.74 | 6.92 | Increase |
| DALYs (Disability-Adjusted Life Years) | 4 | Eswatini | 17.23 | 16.56 | -0.67 | -3.91 | Decrease |
| DALYs (Disability-Adjusted Life Years) | 4 | Botswana | 10.96 | 13.43 | 2.47 | 22.51 | Increase |
| DALYs (Disability-Adjusted Life Years) | 5 | Mali | 124.52 | 136 | 11.48 | 9.22 | Increase |
| DALYs (Disability-Adjusted Life Years) | 5 | Ghana | 59.6 | 69.78 | 10.19 | 17.09 | Increase |
| DALYs (Disability-Adjusted Life Years) | 5 | Sierra Leone | 53.57 | 60.33 | 6.76 | 12.61 | Increase |
| DALYs (Disability-Adjusted Life Years) | 5 | Liberia | 54.62 | 58.62 | 4.01 | 7.33 | Increase |
| DALYs (Disability-Adjusted Life Years) | 5 | Mauritania | 51.67 | 55.5 | 3.83 | 7.42 | Increase |
| DALYs (Disability-Adjusted Life Years) | 5 | Côte d'Ivoire | 42.45 | 50.52 | 8.07 | 19.01 | Increase |
| DALYs (Disability-Adjusted Life Years) | 5 | Guinea-Bissau | 40.84 | 45.63 | 4.79 | 11.73 | Increase |
| DALYs (Disability-Adjusted Life Years) | 5 | Togo | 39.45 | 44.03 | 4.58 | 11.62 | Increase |
| DALYs (Disability-Adjusted Life Years) | 5 | Niger | 41.08 | 43.81 | 2.73 | 6.64 | Increase |
| DALYs (Disability-Adjusted Life Years) | 5 | Cabo Verde | 39.27 | 42.04 | 2.77 | 7.06 | Increase |
| DALYs (Disability-Adjusted Life Years) | 5 | Guinea | 37.56 | 40.68 | 3.12 | 8.3 | Increase |
| DALYs (Disability-Adjusted Life Years) | 5 | Burkina Faso | 35.48 | 36.84 | 1.36 | 3.84 | Increase |
| DALYs (Disability-Adjusted Life Years) | 5 | Senegal | 33.74 | 35.9 | 2.17 | 6.42 | Increase |
| DALYs (Disability-Adjusted Life Years) | 5 | Nigeria | 23.85 | 24.79 | 0.94 | 3.92 | Increase |
| DALYs (Disability-Adjusted Life Years) | 5 | Gambia | 17.35 | 21.69 | 4.34 | 25 | Increase |
| DALYs (Disability-Adjusted Life Years) | 5 | Benin | 20.16 | 20.02 | -0.14 | -0.7 | Decrease |
| Deaths | 1 | Libya | 5.22 | 5.15 | -0.07 | -1.38 | Decrease |
| Deaths | 1 | Egypt | 4.3 | 4.24 | -0.06 | -1.32 | Decrease |
| Deaths | 1 | Tunisia | 4.39 | 4.16 | -0.24 | -5.4 | Decrease |
| Deaths | 1 | Sudan | 2.95 | 3.07 | 0.12 | 4.12 | Increase |
| Deaths | 1 | Algeria | 1.94 | 2.08 | 0.14 | 7.36 | Increase |
| Deaths | 1 | Morocco | 1.81 | 2.05 | 0.24 | 13.26 | Increase |
| Deaths | 2 | Sao Tome and Principe | 5.29 | 5.7 | 0.42 | 7.91 | Increase |
| Deaths | 2 | Equatorial Guinea | 3.67 | 4 | 0.33 | 8.91 | Increase |
| Deaths | 2 | Congo | 2.81 | 3.23 | 0.42 | 15.06 | Increase |
| Deaths | 2 | Gabon | 2.79 | 3.17 | 0.38 | 13.67 | Increase |
| Deaths | 2 | Democratic Republic of the Congo | 2.43 | 2.91 | 0.48 | 19.76 | Increase |
| Deaths | 2 | Cameroon | 2.74 | 2.87 | 0.13 | 4.66 | Increase |
| Deaths | 2 | Chad | 2.38 | 2.51 | 0.14 | 5.83 | Increase |
| Deaths | 2 | Angola | 2.23 | 2.24 | 0.01 | 0.36 | Increase |
| Deaths | 2 | Central African Republic | 1.5 | 1.61 | 0.11 | 7.27 | Increase |
| Deaths | 3 | Malawi | 6.62 | 7.66 | 1.05 | 15.79 | Increase |
| Deaths | 3 | Zambia | 3.75 | 4.23 | 0.48 | 12.83 | Increase |
| Deaths | 3 | Ethiopia | 3.21 | 3.82 | 0.61 | 18.95 | Increase |
| Deaths | 3 | Rwanda | 3.42 | 3.68 | 0.26 | 7.5 | Increase |
| Deaths | 3 | Madagascar | 3.37 | 3.66 | 0.29 | 8.52 | Increase |
| Deaths | 3 | Seychelles | 3.47 | 3.1 | -0.37 | -10.61 | Decrease |
| Deaths | 3 | South Sudan | 2.79 | 2.87 | 0.08 | 2.92 | Increase |
| Deaths | 3 | United Republic of Tanzania | 2.49 | 2.49 | -0.01 | -0.25 | Decrease |
| Deaths | 3 | Burundi | 2.22 | 2.4 | 0.18 | 8.19 | Increase |
| Deaths | 3 | Uganda | 2.3 | 2.4 | 0.1 | 4.33 | Increase |
| Deaths | 3 | Eritrea | 2.03 | 2.31 | 0.29 | 14.08 | Increase |
| Deaths | 3 | Djibouti | 2.13 | 2.3 | 0.17 | 7.93 | Increase |
| Deaths | 3 | Mozambique | 2.15 | 2.25 | 0.1 | 4.56 | Increase |
| Deaths | 3 | Mauritius | 1.92 | 1.75 | -0.17 | -9.01 | Decrease |
| Deaths | 3 | Somalia | 1.06 | 1.2 | 0.15 | 13.72 | Increase |
| Deaths | 3 | Comoros | 1.07 | 1.18 | 0.11 | 10 | Increase |
| Deaths | 3 | Kenya | 1.03 | 1.13 | 0.09 | 9.08 | Increase |
| Deaths | 4 | Zimbabwe | 3.08 | 3.2 | 0.11 | 3.69 | Increase |
| Deaths | 4 | South Africa | 2.18 | 2.09 | -0.09 | -4.05 | Decrease |
| Deaths | 4 | Lesotho | 1.29 | 1.44 | 0.14 | 11.1 | Increase |
| Deaths | 4 | Namibia | 1.02 | 1.08 | 0.07 | 6.65 | Increase |
| Deaths | 4 | Eswatini | 0.67 | 0.68 | 0.01 | 1 | Increase |
| Deaths | 4 | Botswana | 0.48 | 0.58 | 0.1 | 21.81 | Increase |
| Deaths | 5 | Mali | 5.6 | 6.03 | 0.43 | 7.74 | Increase |
| Deaths | 5 | Ghana | 2.5 | 2.91 | 0.41 | 16.26 | Increase |
| Deaths | 5 | Sierra Leone | 2.43 | 2.71 | 0.28 | 11.32 | Increase |
| Deaths | 5 | Mauritania | 2.49 | 2.67 | 0.19 | 7.5 | Increase |
| Deaths | 5 | Liberia | 2.45 | 2.56 | 0.11 | 4.42 | Increase |
| Deaths | 5 | Côte d'Ivoire | 1.97 | 2.27 | 0.29 | 14.79 | Increase |
| Deaths | 5 | Guinea-Bissau | 1.95 | 2.15 | 0.2 | 10.12 | Increase |
| Deaths | 5 | Niger | 2.02 | 2.14 | 0.12 | 5.85 | Increase |
| Deaths | 5 | Togo | 1.81 | 1.98 | 0.17 | 9.51 | Increase |
| Deaths | 5 | Guinea | 1.8 | 1.96 | 0.16 | 8.77 | Increase |
| Deaths | 5 | Burkina Faso | 1.84 | 1.92 | 0.08 | 4.62 | Increase |
| Deaths | 5 | Senegal | 1.6 | 1.7 | 0.1 | 6.03 | Increase |
| Deaths | 5 | Cabo Verde | 1.65 | 1.64 | -0.01 | -0.31 | Decrease |
| Deaths | 5 | Nigeria | 1.17 | 1.23 | 0.06 | 5.24 | Increase |
| Deaths | 5 | Gambia | 0.8 | 1 | 0.2 | 24.6 | Increase |
| Deaths | 5 | Benin | 0.9 | 0.89 | -0.01 | -1.18 | Decrease |
| Incidence | 1 | Tunisia | 9.84 | 10.15 | 0.31 | 3.13 | Increase |
| Incidence | 1 | Libya | 10.27 | 9.89 | -0.38 | -3.71 | Decrease |
| Incidence | 1 | Egypt | 8.08 | 8.32 | 0.23 | 2.88 | Increase |
| Incidence | 1 | Sudan | 5.05 | 5.61 | 0.56 | 11.09 | Increase |
| Incidence | 1 | Algeria | 3.98 | 4.6 | 0.62 | 15.53 | Increase |
| Incidence | 1 | Morocco | 3.03 | 3.66 | 0.64 | 21.08 | Increase |
| Incidence | 2 | Sao Tome and Principe | 8.93 | 10.24 | 1.31 | 14.65 | Increase |
| Incidence | 2 | Equatorial Guinea | 5.97 | 6.79 | 0.82 | 13.64 | Increase |
| Incidence | 2 | Gabon | 4.43 | 5.25 | 0.82 | 18.4 | Increase |
| Incidence | 2 | Congo | 4.12 | 4.89 | 0.77 | 18.72 | Increase |
| Incidence | 2 | Cameroon | 4.05 | 4.45 | 0.4 | 9.75 | Increase |
| Incidence | 2 | Democratic Republic of the Congo | 3.54 | 4.41 | 0.88 | 24.76 | Increase |
| Incidence | 2 | Angola | 3.26 | 3.4 | 0.14 | 4.3 | Increase |
| Incidence | 2 | Chad | 3.17 | 3.4 | 0.23 | 7.21 | Increase |
| Incidence | 2 | Central African Republic | 1.94 | 2.12 | 0.18 | 9.39 | Increase |
| Incidence | 3 | Malawi | 10.55 | 12.68 | 2.13 | 20.16 | Increase |
| Incidence | 3 | Zambia | 5.97 | 7.01 | 1.05 | 17.54 | Increase |
| Incidence | 3 | Seychelles | 7.13 | 6.63 | -0.5 | -7.01 | Decrease |
| Incidence | 3 | Ethiopia | 4.99 | 6.28 | 1.29 | 25.83 | Increase |
| Incidence | 3 | Rwanda | 5.42 | 6.13 | 0.71 | 13.15 | Increase |
| Incidence | 3 | Madagascar | 4.79 | 5.34 | 0.55 | 11.53 | Increase |
| Incidence | 3 | South Sudan | 4.06 | 4.17 | 0.11 | 2.79 | Increase |
| Incidence | 3 | Uganda | 3.71 | 4.01 | 0.3 | 8.19 | Increase |
| Incidence | 3 | Mauritius | 4.23 | 3.94 | -0.29 | -6.8 | Decrease |
| Incidence | 3 | Djibouti | 3.44 | 3.9 | 0.47 | 13.61 | Increase |
| Incidence | 3 | United Republic of Tanzania | 3.75 | 3.78 | 0.02 | 0.59 | Increase |
| Incidence | 3 | Burundi | 3.36 | 3.75 | 0.39 | 11.58 | Increase |
| Incidence | 3 | Eritrea | 3.16 | 3.72 | 0.57 | 17.89 | Increase |
| Incidence | 3 | Mozambique | 2.99 | 3.19 | 0.19 | 6.48 | Increase |
| Incidence | 3 | Kenya | 1.68 | 1.91 | 0.23 | 13.62 | Increase |
| Incidence | 3 | Comoros | 1.62 | 1.78 | 0.15 | 9.37 | Increase |
| Incidence | 3 | Somalia | 1.43 | 1.65 | 0.21 | 14.87 | Increase |
| Incidence | 4 | Zimbabwe | 4.81 | 5.01 | 0.2 | 4.18 | Increase |
| Incidence | 4 | South Africa | 3.74 | 3.68 | -0.06 | -1.62 | Decrease |
| Incidence | 4 | Lesotho | 1.9 | 2.15 | 0.25 | 12.87 | Increase |
| Incidence | 4 | Namibia | 1.78 | 1.99 | 0.21 | 11.63 | Increase |
| Incidence | 4 | Eswatini | 1.12 | 1.14 | 0.02 | 1.56 | Increase |
| Incidence | 4 | Botswana | 0.83 | 1.09 | 0.26 | 30.74 | Increase |
| Incidence | 5 | Mali | 8.34 | 9.25 | 0.92 | 11 | Increase |
| Incidence | 5 | Ghana | 4.2 | 5.1 | 0.9 | 21.43 | Increase |
| Incidence | 5 | Mauritania | 3.98 | 4.45 | 0.48 | 11.97 | Increase |
| Incidence | 5 | Sierra Leone | 3.66 | 4.25 | 0.59 | 16 | Increase |
| Incidence | 5 | Liberia | 3.81 | 4.17 | 0.36 | 9.44 | Increase |
| Incidence | 5 | Cabo Verde | 3.32 | 3.64 | 0.32 | 9.73 | Increase |
| Incidence | 5 | Côte d'Ivoire | 2.95 | 3.59 | 0.64 | 21.54 | Increase |
| Incidence | 5 | Togo | 2.71 | 3.11 | 0.4 | 14.74 | Increase |
| Incidence | 5 | Niger | 2.8 | 3 | 0.2 | 7.12 | Increase |
| Incidence | 5 | Guinea-Bissau | 2.63 | 2.99 | 0.36 | 13.66 | Increase |
| Incidence | 5 | Guinea | 2.51 | 2.79 | 0.28 | 11.17 | Increase |
| Incidence | 5 | Burkina Faso | 2.47 | 2.6 | 0.12 | 4.96 | Increase |
| Incidence | 5 | Senegal | 2.38 | 2.6 | 0.22 | 9.34 | Increase |
| Incidence | 5 | Nigeria | 1.76 | 1.9 | 0.14 | 8.06 | Increase |
| Incidence | 5 | Gambia | 1.2 | 1.51 | 0.31 | 26.32 | Increase |
| Incidence | 5 | Benin | 1.38 | 1.42 | 0.03 | 2.49 | Increase |
| Prevalence | 1 | Tunisia | 58.04 | 68.17 | 10.13 | 17.45 | Increase |
| Prevalence | 1 | Libya | 46.94 | 42.29 | -4.65 | -9.91 | Decrease |
| Prevalence | 1 | Egypt | 35.79 | 36 | 0.21 | 0.58 | Increase |
| Prevalence | 1 | Sudan | 21.33 | 24.83 | 3.5 | 16.42 | Increase |
| Prevalence | 1 | Algeria | 19.24 | 22.66 | 3.43 | 17.82 | Increase |
| Prevalence | 1 | Morocco | 10.85 | 13.08 | 2.23 | 20.56 | Increase |
| Prevalence | 2 | Sao Tome and Principe | 33.98 | 40.04 | 6.06 | 17.83 | Increase |
| Prevalence | 2 | Equatorial Guinea | 19.72 | 21.83 | 2.11 | 10.7 | Increase |
| Prevalence | 2 | Gabon | 16.73 | 20.21 | 3.48 | 20.82 | Increase |
| Prevalence | 2 | Cameroon | 13.45 | 15.51 | 2.06 | 15.34 | Increase |
| Prevalence | 2 | Congo | 13.12 | 15.41 | 2.3 | 17.53 | Increase |
| Prevalence | 2 | Democratic Republic of the Congo | 11.88 | 15.3 | 3.43 | 28.84 | Increase |
| Prevalence | 2 | Angola | 11.15 | 12.14 | 0.99 | 8.89 | Increase |
| Prevalence | 2 | Chad | 9.36 | 9.99 | 0.64 | 6.79 | Increase |
| Prevalence | 2 | Central African Republic | 5.81 | 6.57 | 0.76 | 13.09 | Increase |
| Prevalence | 3 | Malawi | 38.45 | 45.35 | 6.9 | 17.95 | Increase |
| Prevalence | 3 | Seychelles | 37.68 | 37.02 | -0.66 | -1.75 | Decrease |
| Prevalence | 3 | Zambia | 21.45 | 25.07 | 3.62 | 16.87 | Increase |
| Prevalence | 3 | Rwanda | 20.45 | 23.98 | 3.52 | 17.23 | Increase |
| Prevalence | 3 | Ethiopia | 17.77 | 22.78 | 5.01 | 28.18 | Increase |
| Prevalence | 3 | Mauritius | 23.1 | 22.06 | -1.04 | -4.48 | Decrease |
| Prevalence | 3 | Madagascar | 15.66 | 17.72 | 2.06 | 13.13 | Increase |
| Prevalence | 3 | Uganda | 15.48 | 17.38 | 1.91 | 12.33 | Increase |
| Prevalence | 3 | Djibouti | 14.28 | 17.01 | 2.72 | 19.07 | Increase |
| Prevalence | 3 | Eritrea | 12.61 | 15.32 | 2.71 | 21.5 | Increase |
| Prevalence | 3 | South Sudan | 14.2 | 14.16 | -0.04 | -0.31 | Decrease |
| Prevalence | 3 | United Republic of Tanzania | 13.93 | 14.08 | 0.15 | 1.06 | Increase |
| Prevalence | 3 | Burundi | 12.3 | 14 | 1.7 | 13.81 | Increase |
| Prevalence | 3 | Mozambique | 11.97 | 13.44 | 1.48 | 12.35 | Increase |
| Prevalence | 3 | Kenya | 6.37 | 7.43 | 1.06 | 16.68 | Increase |
| Prevalence | 3 | Comoros | 6.58 | 7.05 | 0.47 | 7.13 | Increase |
| Prevalence | 3 | Somalia | 5.36 | 6.2 | 0.84 | 15.66 | Increase |
| Prevalence | 4 | Zimbabwe | 18.73 | 19.02 | 0.28 | 1.52 | Increase |
| Prevalence | 4 | South Africa | 17.1 | 17.14 | 0.04 | 0.25 | Increase |
| Prevalence | 4 | Namibia | 8.13 | 8.94 | 0.82 | 10.04 | Increase |
| Prevalence | 4 | Lesotho | 6.43 | 6.77 | 0.35 | 5.4 | Increase |
| Prevalence | 4 | Eswatini | 5.59 | 5.61 | 0.02 | 0.4 | Increase |
| Prevalence | 4 | Botswana | 2.91 | 4.1 | 1.19 | 40.68 | Increase |
| Prevalence | 5 | Mali | 29.33 | 33.18 | 3.84 | 13.1 | Increase |
| Prevalence | 5 | Ghana | 16.9 | 20.67 | 3.77 | 22.33 | Increase |
| Prevalence | 5 | Sierra Leone | 13.16 | 15.52 | 2.37 | 17.98 | Increase |
| Prevalence | 5 | Mauritania | 13.57 | 15.44 | 1.87 | 13.77 | Increase |
| Prevalence | 5 | Liberia | 13.13 | 14.35 | 1.21 | 9.23 | Increase |
| Prevalence | 5 | Cabo Verde | 13.86 | 14.31 | 0.45 | 3.25 | Increase |
| Prevalence | 5 | Côte d'Ivoire | 10.65 | 13.65 | 2.99 | 28.08 | Increase |
| Prevalence | 5 | Togo | 9.72 | 11.45 | 1.73 | 17.78 | Increase |
| Prevalence | 5 | Senegal | 8.79 | 9.69 | 0.9 | 10.23 | Increase |
| Prevalence | 5 | Guinea | 8.44 | 9.55 | 1.11 | 13.18 | Increase |
| Prevalence | 5 | Guinea-Bissau | 8.09 | 9.53 | 1.45 | 17.91 | Increase |
| Prevalence | 5 | Niger | 8.48 | 9.09 | 0.61 | 7.2 | Increase |
| Prevalence | 5 | Burkina Faso | 7.74 | 8.09 | 0.34 | 4.42 | Increase |
| Prevalence | 5 | Nigeria | 6.21 | 6.81 | 0.59 | 9.55 | Increase |
| Prevalence | 5 | Benin | 5.13 | 5.49 | 0.36 | 7.05 | Increase |
| Prevalence | 5 | Gambia | 4.3 | 5.31 | 1 | 23.35 | Increase |
